# High endemism at cave entrances: a case study of spiders of the genus *Uthina*

**DOI:** 10.1038/srep35757

**Published:** 2016-10-24

**Authors:** Zhiyuan Yao, Tingting Dong, Guo Zheng, Jinzhong Fu, Shuqiang Li

**Affiliations:** 1 Key Laboratory of Zoological Systematics and Evolution, Institute of Zoology, Chinese Academy of Sciences, Beijing 100101, China; 2 Southeast Asia Biodiversity Research Institute, Chinese Academy of Sciences, Menglun, Mengla, Yunnan 666303, China; 3 Department of Integrative Biology, College of Biological Sciences, University of Guelph, Guelph N1G2W1, Canada; 4 College of Life Sciences, Shenyang Normal University, Shenyang 110034, China

## Abstract

Endemism, which is typically high on islands and in caves, has rarely been studied in the cave entrance ecotone. We investigated the endemism of the spider genus *Uthina* at cave entrances. Totally 212 spiders were sampled from 46 localities, from Seychelles across Southeast Asia to Fiji. They mostly occur at cave entrances but occasionally appear at various epigean environments. Phylogenetic analysis of DNA sequence data from COI and 28S genes suggested that *Uthina* was grouped into 13 well-supported clades. We used three methods, the Bayesian Poisson Tree Processes (bPTP) model, the Bayesian Phylogenetics and Phylogeography (BPP) method, and the general mixed Yule coalescent (GMYC) model, to investigate species boundaries. Both bPTP and BPP identified the 13 clades as 13 separate species, while GMYC identified 19 species. Furthermore, our results revealed high endemism at cave entrances. Of the 13 provisional species, twelve (one known and eleven new) are endemic to one or a cluster of caves, and all of them occurred only at cave entrances except for one population of one species. The only widely distributed species, *U. luzonica*, mostly occurred in epigean environments while three populations were found at cave entrances. Additionally, eleven new species of the genus are described.

The global distribution of endemism is very uneven. Certain areas or habitats possess particularly high endemism, and these areas are often designated as high priority for conservation^1^. Such areas also provide excellent study systems for understanding ecosystem function and evolutionary processes^2^,^3^. Islands are often cited as examples for high endemism, and geographic isolation and limited interchange with neighboring mainland or island biota are the primary causes^4^,^5^. Moreover, neoendemic taxa are generated from recent and rapid adaptive radiations in volcanic archipelagos like the Canary Islands or Hawaii^6^,^7^,^8^, and distinct paleoendemic lineages are harbored in some ancient continental fragments like Madagascar or New Zealand^8^.

Caves, functioning as biological islands, are no exception. The typical cave environment in the deep interior is usually considered climatically stable, possessing constant temperature and humidity, with complete darkness and scarce energy sources^9^. These extreme environmental conditions and the limited dispersal ability of cave-dwellers have been regarded as the primary factors of maintaining endemism within caves^10^,^11^. Many studies have documented the high levels of species endemism within various cave systems^12^,^13^,^14^,^15^. Nevertheless, as part of the cave system, the importance of cave entrance area has largely been ignored.

Cave entrances possess several unique traits that make them potentially areas of high endemism. They represent the transition zones between epigean and hypogean environments. Similar to deep cave zones, they are more climatically stable than the epigean environments, although they do subject to climatic variations in the external environment, undergoing daily and annual variations in temperature, humidity and light^16^,^17^. Furthermore, the direct or even reflected light at the entrances allows the growth of photosynthetic organisms that result in higher food resources than inside of caves^18^,^19^,^20^. Prous *et al*.^17^ characterized cave entrance as a separate ecotone and further divided cave entrances into para-hypogean and para-epigean zones. Organisms often specialize in an ecotone because in these places they can find suitable supporting conditions unavailable in other environments or they depend on two or more structurally different and contiguous habitats^17^,^21^,^22^,^23^,^24^. With these intermediate characteristics of cave entrances, we expect that many sedentary organisms would be specialized to the cave entrance environment. Therefore, we predict that, similar to caves, the unique habitat conditions of cave entrances and the potentially reduced interchange between populations from geographically separate cave entrances likely promote the development of high endemism.

Pholcid spiders of the genus *Uthina* (family Pholcidae) primarily occur in cave entrances, although they can be found in other well-covered microhabitats, such as under rocks and leaf litter, in bamboo internodes and tree holes, and occasionally in rather open areas. It is a group of haplogyne spiders with a medium sized body, a cylindrical abdomen, and very long legs. They are widely distributed from the Seychelles and Sri Lanka, across Southeast Asia, to north Australia and Fiji (Fig. 1). Currently, the genus includes only three described species, *U. luzonica*, *U. ratchaburi*, and *U. khaosokensis*^25^,^26^, for which morphology characters are very similar and provide little diagnostic information, and according to Huber’s^25^ observations, the variation mainly occurs in the uncus and the appendix, females were essentially indistinguishable. Similar to many other morphologically conservative and grossly under-studied spider groups, we expect that there are many more undescribed species in the genus^15^,^27^,^28^.

In this study, we examined the species diversity and endemism of *Uthina* spiders around the entrance areas of caves. We extensively sampled the group throughout their distribution ranges, including cave entrances and various epigean habitats. Primarily based DNA sequence data from both nuclear and mitochondrial genes, we defined their species boundaries and evaluated their levels of endemism at the cave entrance ecotone. Furthermore, we provided morphology-based species descriptions of the 11 new species in the genus.

## Results

### Sequence data characteristics

A total of 212 sequences of COI and 212 sequences of 28S from 212 ingroup members, and three sequences of COI and two sequences of 28S from three outgroup members were successfully generated. The alignment of COI did not introduce any gaps, and after trimming the ends, 1209 base pairs (bp) were resolved. Among them, 374 sites were variable and 359 sites were parsimony informative (excluding outgroups), and 64 haplotypes were identified. For the 28S gene fragment, several gap regions were introduced after alignment, and none of the regions were hyper-variable. Therefore, all sites were retained, which produced a total of 1111 bp. Among them, 35 sites were variable and 32 sites were parsimony informative (excluding outgroups), and 22 haplotypes were identified. The numbers of individuals that share the same haplotype for COI, 28S, and concatenated sequences are presented in Table S3.

### Phylogenetic relationships

Separated analyses of individual genes and concatenated data analysis found largely compatible topologies; disagreements were primarily among clades with low supporting values. The BI and ML analyses of the concatenated data supported the same topology and Fig. 2 presents the tree from the BI analysis. The genus *Uthina* was clearly divided into 13 well-supported major clades (Fig. 2; Fig. S1). Most of these clades consisted of samples from the entrance area of a single cave or cave cluster. Nevertheless, the clade *U. huifengi* included samples from entrance areas of one cave cluster (sites 35–42) and an epigean site (site 43). The clade *U. wongpromi* included samples from a cave cluster (sites 18–19) and another cave (site 20) that was geographically far away (approximately 750 km) from the cave cluster. All samples from the widespread species, *U. luzonica*, formed one major clade, and most of the samples were from epigean environments with three populations from cave entrances (sites 5, 12, 17). While these major clades were well-defined and mostly well-supported, relationships among these clades were not well resolved, especially among the more basal branches. For the COI gene tree, the major clade memberships were consistent with these of concatenated gene tree, but the clade *U. huifengi* was further divided into two clades. The Bayesian tree and likelihood tree divided the clade differently (Fig. S2). Not surprisingly, the 28S gene tree was much less resolved (Fig. S2), likely due to the limited number of informative characters of the gene.

Average genetic distances among the 13 major clades and the maximum distances within the major clades estimated from the COI data are presented in Table 1. Both uncorrected p-distances and the K2P distances were estimated. The pairwise p-distances ranged from 2.7–14.3% among the major clades and within these clades, the distances ranged from 0–2.5%. The ranked pairwise distances are presented in Fig. 3. A gap was apparent with the distance values ranging 3.6–8.3%.

### Species delimitation

The bPTP analysis of the COI gene data based on its ML tree topology identified 13 provisional species. Since its BI tree topology was slightly different from its ML tree, we also conducted the same analysis based on the BI tree topology, and the analysis identified 14 provisional species. The clade *U. huifengi* was further divided into two species *U. huifengi*_a and *U. huifengi*_b (Fig. 2; Fig. S3). The bPTP analysis of the 28S gene data identified 16 and 20 species based on its BI and ML tree topologies respectively; however, the BS values of most species were very low (Fig. S4).

The GMYC analysis of COI gene identified 19 species. Compared to the results of bPTP, *U. huifengi* was recognized as four separate species, *U. huahinensis* was divided into three species, and *U. luzonica* was delimited to two species (Fig. S5). For 28S gene, the GMYC analysis identified only one species (result not shown).

The BPP analysis requires pre-defined species. We adopted a conservative approach and used BPP to validate the 13 and 14 species identified by the bPTP analysis of COI gene. The BPP analyses found very high probabilities of speciation events for most of the nodes tested using all four prior combinations (Fig. 2). In particular, prior combinations of (ii) and (iv) produced speciation probabilities of one for most nodes (Fig. 2). These results were also consistent across multiple runs.

### Morphological variation

Variation at the morphological level was limited. Most variations occur in the uncus (shape, as description in Huber^25^), the appendix (shape, as description in Huber^25^), the angular apophysis on the procursus (presence or absence, size, position, distally bifurcated or not), the knob of female external genitalia (distally bifurcated or not, distally swollen or not), and the vulval pore plates (shape, size, distance between two pore plates). More detailed descriptions and illustrations are provided in the Texts S1–S11 and the Figs S6–S29.

## Discussion

### How many species are in the genus *Uthina*?

Considering all evidence, we conclude that there are 13 provisional species in the genus *Uthina*. The phylogenetic tree derived from the concatenated data clearly divided the samples into 13 deeply divergent clades. Moreover, the BPP analysis strongly supports the speciation events among those 13 clades. The bPTP analysis of COI gene tree (ML) also supports 13 species, although the BS values of *U. huifengi* clade and *U. luzonica* clade are relatively low (0.50, 0.54). Finally, we detected a clear gap in pairwise COI p-distances distribution (Fig. 3), and distance values between the majority of the 13 provisional species are above the gap (>8.3%). Only distance between *U. yunchuni* and *U. zhigangi* and distance between *U. wongpromi* and *U. luzonica* are below the gap (<3.6%). Nevertheless, they (2.7–3.6%) are still higher than the often-cited barcoding 2% cut-off value for species-level divergence^29^. The smallest p-distance among species is between *U. yunchuni* and *U. zhigangi* (2.7%; Table 1), however, the speciation event is well supported by bPTP, GMYC and BPP analyses. In the case of *U. wongpromi* and *U. luzonica*, the BPP analysis unambiguously supports their species status (BPP = 1), although the bPTP analysis only provides a low confidence support (BS value = 0.54) for *U. luzonica*. The low BS value is likely a consequence of relatively high-level of divergence within this species and relative short divergence time between *U. wongpromi* and *U. luzonica*. It is worth noting that bPTP analysis of COI gene data and the BPP analysis also suggested 14 species, in which *U. huifengi* is further divided into two species. Nevertheless, the BS values of both species are relatively low (0.61, 0.86), and neither of them form monophyletic group on the preferred phylogenetic tree (Fig. 2). Therefore, we take a more conservative approach and treat the *U. huifengi* clade as one single species.

Accurate and rapid assessment of diversity is essential to conservation. Several molecular-based methods of species delimitation have been proposed and often applied^30^,^31^,^32^. We employed three commonly-used methods for the genus *Uthina*, and produced quite different results. The results from COI-based bPTP analysis and BPP analysis were highly consistent (13–14 species), but the GMYC analysis for COI gene identified many more species (19 species). Furthermore, analysis of the 28S gene produced the most different results. The bPTP analysis suggested 16 to 20 species (with low BS values) while the GMYC analysis concluded one species. This drastic difference is probably caused by weak signal, since the gene exhibited only 35 variable sites. Clearly, data with adequate information is essential to produce reliable results. Various methods may produce difference conclusions, and strengths and weakness of each method need to be further explored and evaluated.

### High endemism at cave entrance

Our study revealed extremely high endemism at the cave entrance ecotone. Of the 13 provisional species identified in this study, 12 are endemic to one cave or cave cluster. It is noteworthy that six species are endemic to one single cave and six species are from several caves that are in geographic proximity (cave cluster). *U. wongpromi* is the only exception, of which one of its populations (site 20) is approximately 750 km away from the other two populations (sites 18–19). Furthermore, all these 12 species are only found at cave entrances except one population. Individuals of *U. huifengi* were found from epigean environment at site 43, while all other eight populations of the same species are from cave entrance. Another endemic species, *U. khaosokensis*^26^, which is not included in this study due to the lack of DNA sample, also occurs at one single cave entrance of Thailand (Huber, personal communication).

*U. luzonica* is the only widespread species in the genus. It is known from the Seychelles, Sri Lanka, across Southeast Asia, reaching northern Australia and Fiji. While three of its populations (sites 5, 12, 17) are found from cave entrance ecotone, the majority of its 17 populations are collected from epigean environments.

The *Uthina* spiders may have been specialized to the cave entrance ecotone, although we have not detected any particular adaptive or physiological traits. Such specialization may result in restricted dispersal and interchange among populations between different cave entrances, and consequently cause geographic isolation and speciation^33^,^34^. The neoendemic species inhabit respective cave entrances, in the similar fashion as on islands^6^,^7^,^8^. Conversely, species with expanded ecological niches, such as *U. luzonica* that occurs not only at cave entrances but also at various epigenan environments, may reach a much wider distribution. The species *U. wongpromi* is also distributed in a fairly large area, with one population (site 20) approximately 750 km away from the other two populations (sites 18–19). However, all of its populations were found from cave entrances. A probable explanation is that the population from site 20 is the result of a recent human-assisted dispersal, since all the three populations share the same one haplotype. There have been several similar cases known in its closely-related genus *Pholcus*^25^, although we do not have direct evidences. Caves in Thailand are often accessed by humans for religious purposes, which may also facilitate human-assisted dispersal.

Prous *et al*.^35^ viewed studies of cave entrances paramount for any action toward their conservation and management, and our work clearly supports their opinion. Several previous studies revealed high endemism^13^,^14^ and high cryptic species diversity^15^,^36^,^37^ within cave systems. Studies for cave entrances, however, are primarily focused on species diversity and richness^17^,^35^,^38^. Our study demonstrated that the cave entrance ecotone has not only high species diversity but also high endemism. Therefore, a cave entrance is not an insignificant attachment of the cave system; rather, it is a unique ecotone, has high diversity and endemism, and deserves high conservation priority.

### Taxonomic implications

The taxonomy of the genus *Uthina* is challenging because of its morphological conservatism. Currently, the genus includes only three described species, *U. luzonica*, *U. ratchaburi*, and *U. khaosokensis*^25^,^26^. The morphology of the genus is extremely conservative. Huber^25^ treated most *Uthina* specimens as a single widespread species *U. luzonica* and described five different morph-types based on male genitalia. In this study, sites 16 and 23 are the type localities of *U. luzonica* and *U. ratchaburi*, respectively and both represent valid species. Although the third species *U. khaosokensis* is not included in this study due to the lack of DNA samples, it can be easily distinguished from other species morphologically^26^. Therefore, of the 13 molecular provisional species, eleven are new species.

Among the 11 new species, several species are similar to three of the five morph-types of ‘*U. luzonica*’ in Huber^25^. For example, the uncus, the appendix, and the female genitalia of *U. huifengi* are very similar to those of the specimens from Sumatra in Huber^25^. The uncus of *U. huahinensis* and *U. wongpromi* are similar to those of the specimens from Sumatra and Fiji, respectively, but the appendix of *U. huahinensis* and *U. wongpromi* have a subdistal pointed projection to make it appear bifid and the vulval pore plates of *U. huahinensis* wide anteriorly and narrow posteriorly. The uncus of *U. sulawesiensis* is also very similar to those of the specimens from North Sulawesi, but the appendix of *U. sulawesiensis* is not bifurcated and the vulval pore plates obviously wide. Among the remaining species, the uncus of *U. muangensis*, *U. potharamens*, and *U. saiyokensis* are not matched with any types of Huber^25^ and are different from each other. Four species, *U. javaensis*, *U. sarikaensis*, *U. yunchuni*, and *U. zhigangi*, do not have male specimens, and we cannot compare their uncus and appendix. Therefore, their species status are based on the molecular data and the morphology of female genitalia. Furthermore, according to the illustrations and the locality information in Huber^25^, the specimens from Sumatra and Fiji in the five morph-types of ‘*U. luzonica*’ likely are *U. huifengi* and true *U. luzonica*, respectively and the specimens from the other three localities (Australia, Palau, and North Sulawesi) likely represent additional species of *Uthina*. Due to the lack of DNA samples and illustrations of the other morphological characters (e.g., procursus), we cannot assign them to any species at present time.

## Methods

### Sample collection

**Figure 1:**
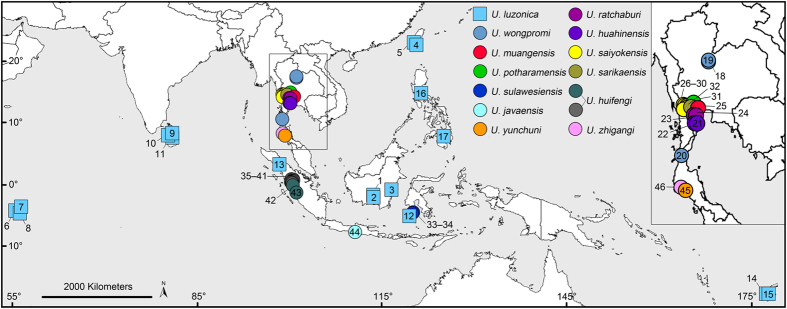
Sampling sites of *Uthina* spiders. Each species is indicated by a different color, which is consistent with the colored branches on the phylogenetic tree in Fig. 2. The insert is a magnified section of the map. Detailed location information is provided in Table S1. Distribution map was generated with ArcView 3.2 (ESRI Inc., Redlands, CA, USA, http://www.esri.com/software/arcgis/arcview).

**Figure 2:**
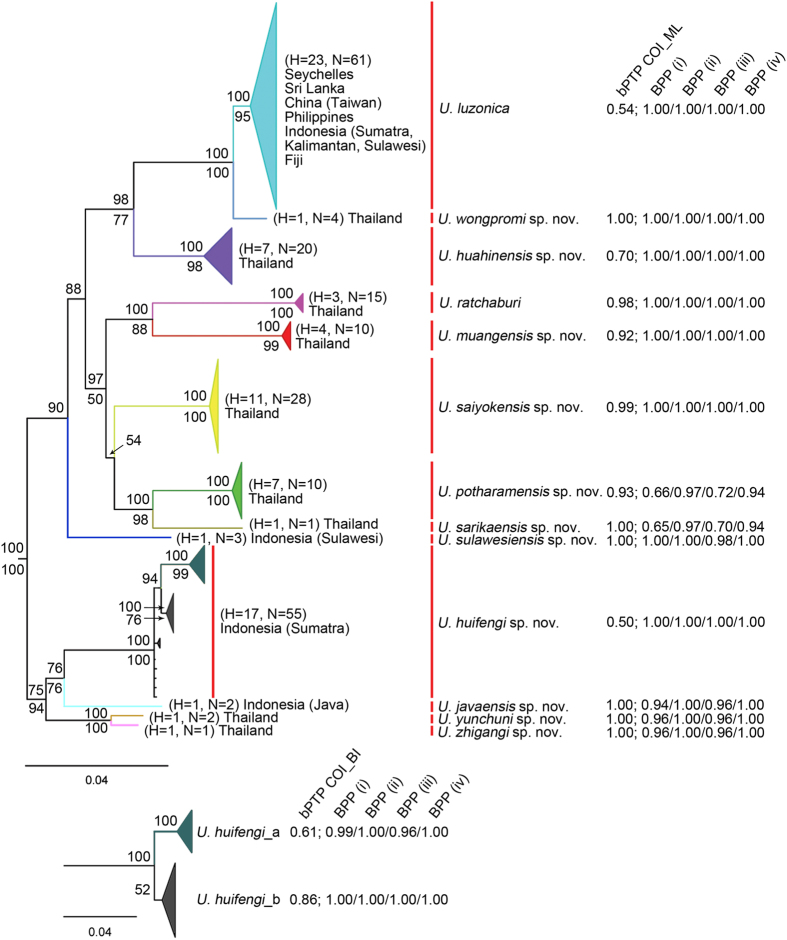
A simplified phylogenetic tree and the results of species delimitation analyses. The tree is derived from a Bayesian analysis of the concatenated data. Bayesian posterior probabilities and bootstrap values from the ML analysis are provided above and below branches, respectively. Bootstrap values below 50 are not shown. For each major clade, H is the number of haplotype in the clade and N is the number of samples in the clade. The full tree is presented in Fig. S1. The Bayesian support values from bPTP (ML tree) and posterior probabilities from BPP are presented on the right. The alternative topology for clade *U. huifengi* derived from the Bayesian analysis of COI and the related species delimitation results are presented at the bottom.

**Figure 3:**
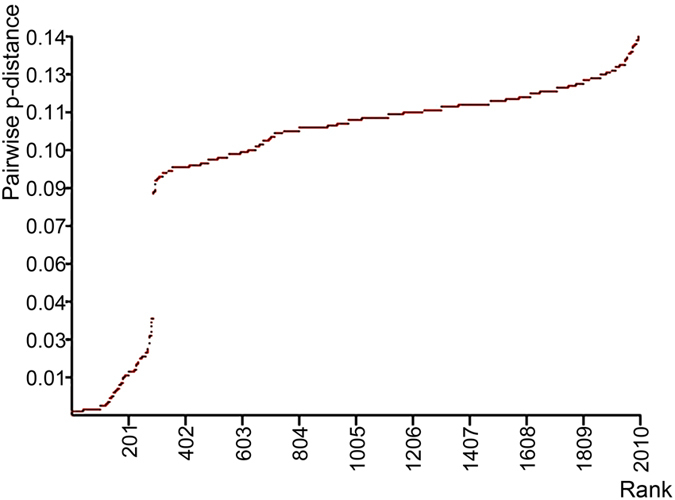
Plot of ranked COI pairwise p-distances. A gap is clearly visable between 3.6–8.3%.

A total of 212 *Uthina* spiders from 46 localities were collected throughout their known distribution ranges including Seychelles, Sri Lanka, Southeast Asia, and Fiji (Fig. 1). Sampling efforts were concentrated at cave entrances (rock walls of the opening) and surrounding epigean environments. To confirm their absence from many epigean habitats and minimize false negative, we searched two opposite sides of each cave entrance extensively, particularly around exposed rocks. We also searched the epigean areas directly opposite to the cave openings. Three species, *Pholcus jiuwei*, *P. phoenixus* and *Leptopholcus podophthalmus*, were selected as outgroups based on previous published phylogenetic tree^25^. Locality information is provided in Table S1. Specimens were preserved in 95% alcohol and stored at −20 °C. All specimens examined are deposited in the Institute of Zoology, Chinese Academy of Sciences (IZCAS) in Beijing, China.

### Laboratory protocols

Genomic DNA was extracted from legs using Ezup Column Animal Genomic DNA Purification Kit by Sangon Biotech (Shanghai, China). After extraction, all DNA samples were preserved in TE buffer and stored in −20 °C.

We targeted two DNA fragments for sequencing, a part of cytochrome oxidase I gene (COI) from the mitochondrial genome and a part of 28S rRNA gene from the nuclear genome. The 28S fragment includes a highly variable region of the gene. Two pairs of primers were used for COI (COI1490 5′-GGTCAACAAATCATAAAGATATTGG-3′, COI2198 5′-TAAACTTCAGGGTGACCAAAAAATCA-3′, COI1718 5′-GGAGGATTTGGAAATTGATTAGTTCC-3′, and COI2776 5′-GGATAATCAGAATATCGTCGAGG-3′^39^,^40^,^41^) and one pair of primers were used for 28S (28SZX1 5′-ACCCGCTGAATTTAAGCATAT-3′ and 28SC 5′-GGTTCGATTAGTCTTTCGCC-3′^41^,^42^). DNA fragments were amplified using PCR on an Eppendorf thermal cycler (Hamburg, Germany) with the following parameters: an initial denaturation at 94 °C for 5 min followed by 35 cycles of denaturation at 94 °C for 30 s, annealing at 45 °C (five cycles) and 50 °C (30 cycles) for 30 s, extension at 72 °C for 30 s, and a final extension at 72 °C for 5 min. PCR products were purified with QIAquick PCR Purification Kit (Qiagen, Hilden, Germany) and then sequenced using BigDye chemistry on an ABI 3730 automated sequencer (Applied Biosystems, Foster City, CA, USA).

### Phylogenetic analysis

DNA sequences were checked and edited with SeqMan II software^43^ and BioEdit 7.2.2^44^. Sequence alignment was completed using CLUSTAL W^45^. Visual inspection, translation, and minor alteration were conducted to minimize alignment errors. Haplotype identification was conducted in DNASP 5.10.1^46^. Genetic distances were computed with MEGA 5^47^. The online version of Automatic Barcode Gap Discovery (ABGD) tool^48^ (http://wwwabi.snv.jussieu.fr/public/abgd/) was used to detect gaps within ranked pairwise distances.

Bayesian inference (BI) and maximum likelihood (ML) methods were used to reconstruct phylogenetic trees. Each haplotype was treated as a taxon and each nucleotide site was treated as a character. The two fragments were analyzed both individually and combined. The best-fit DNA substitution models were selected based on the Akaike information criterion (AIC) using MrModeltest 2.3^49^. Bayesian inference was performed with Mrbayes 3.2.4^50^. For individual gene analysis, the best-fit models GTR + G + I for COI and GTR + G for 28S were selected. For combined gene analysis, the data were separated into gene partitions with each partition following its best-fit model. Four Monte Carlo Markov chains (MCMCs) with default heating parameters were performed for 10 million generations to ensure that the average standard deviation of split frequencies was less than 0.01. Trees were sampled every 1000 generations with the first 50% of sampled trees discarded as burn-in. Bayesian posterior probabilities were estimated on a 50% majority rule consensus tree of the remaining sampled trees. Maximum likelihood analyses were conducted using RAxML 7.0.3^51^, with 1000 rapid bootstrap replicates followed by a thorough maximum-likelihood tree search. For individual gene analysis, the models GTR + G + I for COI and GTR + G for 28S were used. For combined gene analysis, the best-fit model GTR + G + I was selected and used.

### Species delimitation

We applied three methods for species delimitation. A Bayesian implementation of the Poisson Tree Processes (bPTP) model tests species boundaries based on phylogenetic tree of individual gene^52^. The second method, Bayesian Phylogenetics and Phylogeography (BPP), is a multilocus coalescent species delimitation analysis, which requires data from multiple genes and pre-defined candidate species^53^,^54^. We used BPP to test and validate results of the bPTP analysis. We also use the general mixed Yule coalescent (GMYC) model to delimit species from ultrametric tree of individual gene without prior definition of groups^55^,^56^.

The bPTP method uses nucleotide substitution information and implements a model assuming phylogenetic tree branch lengths generated by two classes of Poisson processes (intra and inter-specific branching events). The method produces Bayesian support (BS) values to delimit species on the input tree. A high BS value on a node indicates that all descendants from this node are likely to be from one species. The analyses were conducted on a web server (http://species.h-its.org/ptp/) using our inferred phylogenetic topologies of individual genes. The MCMC was run for 200,000 generations, with a thinning of 100 and burn-in of 0.2.

The BPP method accommodates the species phylogeny as well as lineage sorting due to ancestral polymorphism and estimates the posterior distribution for different species delimitation models. Similar to Leache and Fujita^57^, we conducted four different sets of analyses with different values of *α* and *β*: (i) *Gθ*(1, 10) and *Gτ*(1, 10), assuming large ancestral population sizes and deep divergences between species, (ii) *Gθ*(2, 2000) and *Gτ*(2, 2000), assuming small ancestral populations and shallow divergences, (iii) *Gθ*(1, 10) and *Gτ*(2, 2000), assuming large ancestral populations and shallow divergences, (iv) *Gθ*(2, 2000) and *Gτ*(1, 10), assuming small ancestral populations and deep divergences. The analyses were performed with the following settings: species delimitation = 1, algorithm = 0, finetune = 5. The reversible-jump MCMC analyses were run for 100,000 generations and sampled every two generations, with 25,000 samples being discarded as burn-in. Each set of *α* and *β* was run at least twice to confirm consistency.

The GMYC method identifies a time point on a tree with the highest likelihood where the branching rate shifts from speciation to population coalescent process. The phylogenetic tree was first converted to an ultrametric format using a penalized likelihood method in r8s^58^ with a cross-validation to choose the optimal smoothing parameter of 1. The GMYC analysis was performed under the single-threshold model, using the R 3.2.0 package SPLITS (Species Limits by Threshold Statistics)^59^.

### Endemism definition

A species or other taxon is considered endemic to a particular area if it occurs only in that area^60^. A crucial variable in this definition is the size of the area. In general, the area should be ‘small’; however, it is a relative term, and therefore, depends on the geographic scale of interests. We adopted this definition and defined a species as endemic when it occurs only at the cave entrance area of a single cave or cave cluster. A cave cluster is defined as two or more caves geographically close to each other (Table S2).

### Morphological observation

Specimens for morphological observation were first transferred to 75% alcohol. Male and female genitalia were examined and illustrated after dissection with a Leica M205 C stereomicroscope. Female external genitalia were pre-treated in a warm 10% solution of potassium hydroxide (KOH). Left male pedipalps were illustrated. Images were captured using an Olympus C7070 wide zoom digital camera (7.1 megapixels) mounted on the stereomicroscope, and were montaged using Helicon Focus 6.6.1 image stacking software (http://www.heliconsoft.com/heliconfocus.html). All measurements are given in millimeters. Leg measurements are shown as: Total length (femur + patella + tibia + metatarsus + tarsus). Leg segments were measured on their dorsal side. Morphological descriptions of the 11 new Uthina species can be found in supplementary information accompanies this paper at http://www.nature.com/srep. All the new species are registered in Zoobank at http://zoobank.org/. LSIDs from Zoobank include *U. huahinensis* (urn:lsid:zoobank.org:act:3CE79C7A-9273-4E4B-8B00-FB60E7FEF0AF), *U. huifengi* (urn:lsid:zoobank.org:act:C81D62D1-EE65-421A-B4DD-3515E75D394E), *U. javaensis* (urn:lsid:zoobank.org:act:560EF029-35C9-49CC-81BF-5D42E45CA3C4), *U. muangensis* (urn:lsid:zoobank.org:act:5736AB8A-46E5-4E50-900C-0E7CE3041D87), *U. potharamensis* (urn:lsid:zoobank.org:act:4A034FDA-E209-4534-9881-99C4A22EB961), *U. saiyokensis* (urn:lsid:zoobank.org:act:0780BF58-5336-4F81-8D2E-9DDDEE3CA7A6), *U. sarikaensis* (urn:lsid:zoobank.org:act:B74F0176-11F0-4F91-AE1F-FD5F52A834DC), *U. sulawesiensis* (urn:lsid:zoobank.org:act:4458BB80-B637-4E9D-924E-B02BD4AD5BB3), *U. wongpromi* (urn:lsid:zoobank.org:act:C8BDC3F2-20D7-49E6-A700-4A1E5390015D), *U. yunchuni* (urn:lsid:zoobank.org:act:D94F2B1F-B4A1-4C12-868C-EBF3DE8BB683), *U. zhigangi* (urn:lsid:zoobank.org:act:809004F3-A47B-45B5-BD93-59B0655D5E28). Hard copies on description of new Uthina species are deposited in twenty libraries worldwide.

Terminology and taxonomic descriptions followed Huber^25^. The following abbreviations are used in the descriptions: ALE = anterior lateral eye, AME = anterior median eye, PME = posterior median eye, L/d = length/diameter; used in the illustrations: a = appendix, b = bulb, da = distal apophysis, e = embolus, pa = proximo-lateral apophysis, pp = pore plate, pr = procursus, u = uncus.

## Additional Information

**How to cite this article**: Yao, Z. *et al*. High endemism at cave entrances: a case study of spiders of the genus *Uthina*. *Sci. Rep.* **6**, 35757; doi: 10.1038/srep35757 (2016).

## Supplementary Material

Supplementary Information

## Figures and Tables

**Table 1:** The average uncorrected p-distances (below diagonal) and K2P distances (above diagonal) among the 13 major clades and the maximum p-distances within each major clade (on diagonal) from the COI gene data.

	*luzonica*	*ratchaburi*	*huifengi*	*saiyokensis*	*muangensis*	*huahinensis*	*potharamensis*	*yunchuni*	*sulawesiensis*	*javaensis*	*wongpromi*	*zhigangi*	*sarikaensis*
*U. luzonica*	**0.019**	0.141	0.133	0.117	0.138	0.101	0.123	0.125	0.130	0.142	0.033	0.127	0.127
*U. ratchaburi*	0.127	**0.005**	0.146	0.125	0.135	0.132	0.119	0.154	0.145	0.158	0.147	0.159	0.138
*U. huifengi*	0.120	0.131	**0.025**	0.121	0.155	0.128	0.129	0.102	0.129	0.103	0.141	0.100	0.138
*U. saiyokensis*	0.107	0.113	0.110	**0.005**	0.131	0.109	0.106	0.097	0.106	0.126	0.125	0.096	0.111
*U. muangensis*	0.125	0.122	0.138	0.119	**0.002**	0.123	0.123	0.143	0.127	0.141	0.139	0.144	0.127
*U. huahinensis*	0.093	0.120	0.116	0.100	0.112	**0.016**	0.102	0.128	0.107	0.135	0.104	0.129	0.114
*U. potharamensis*	0.112	0.109	0.117	0.097	0.113	0.095	**0.006**	0.130	0.110	0.127	0.123	0.133	0.091
*U. yunchuni*	0.114	0.137	0.094	0.090	0.128	0.116	0.117	**0**	0.115	0.104	0.133	0.028	0.126
*U. sulawesiensis*	0.118	0.130	0.118	0.097	0.115	0.099	0.101	0.106	**0**	0.117	0.130	0.114	0.107
*U. javaensis*	0.128	0.141	0.094	0.115	0.128	0.122	0.115	0.096	0.108	**0**	0.146	0.101	0.134
*U. wongpromi*	0.032	0.132	0.127	0.114	0.125	0.096	0.112	0.121	0.118	0.131	**0**	0.141	0.128
*U. zhigangi*	0.116	0.141	0.093	0.089	0.130	0.117	0.120	0.027	0.105	0.093	0.127	**0**	0.127
*U. sarikaensis*	0.115	0.124	0.123	0.101	0.115	0.104	0.084	0.114	0.098	0.121	0.117	0.115	**0**
