# Supplementary material for: High endemism at cave entrances: a case study of spiders of the genus Uthina
Source: Sci Rep. 2016 Oct 24;6:35757. doi: 10.1038/srep35757 (PMC5075877; doi:10.1038/srep35757)

SUPPLEMENTARY INFORMATION
High endemism at cave entrances: a case study of spiders of the genus *Uthina*

urn:lsid:zoobank.org:pub:A4549147-13E0-4C93-A334-20EF8B1724F1

Zhiyuan Yao[1, 2, 3], Tingting Dong[4], Guo Zheng[4], Jinzhong Fu[3], Shuqiang Li[1, 2]

[1]Key Laboratory of Zoological Systematics and Evolution, Institute of Zoology, Chinese Academy of Sciences, Beijing 100101, China
[2]Southeast Asia Biodiversity Research Institute, Chinese Academy of Sciences, Menglun, Mengla, Yunnan 666303, China
[3]Department of Integrative Biology, College of Biological Sciences, University of Guelph, Guelph N1G2W1, Canada
[4]College of Life Sciences, Shenyang Normal University, Shenyang 110034, China
Correspondence and requests for materials should be addressed to S.L. (email: lisq@ioz.ac.cn)

TABLE OF CONTENTS

Figure S1. Phylogenetic tree derived from the Bayesian analysis of the concatenated data...III
Figure S2. Individual gene trees……………………………………..………………...IV
Figure S3. Results of bPTP species delimitation based on COI gene tree…………………....V
Figure S4. Results of bPTP species delimitation based on 28S gene tree………………..…VI
Figure S5. Results of GMYC species delimitation based on COI gene tree………………..VII
Table S1. Sampling localities and GenBank accession numbers…………………………..VIII
Table S2. List of cave clusters involved……………………………………………..…...XIV
Table S3. List of haplotypes and samples that share the same haplotype………................XV
Text S1. Description of *Uthina huahinensis* Yao & Li sp. nov…………………………....XIX
Text S2. Description of *Uthina huifengi* Yao & Li sp. nov……………………………..….XX
Text S3. Description of *Uthina javaensis* Yao & Li sp. nov………………………….…....XXI
Text S4. Description of *Uthina muangensis* Yao & Li sp. nov…………………………....XXII
Text S5. Description of *Uthina potharamensis* Yao & Li sp. nov……………………….XXIII
Text S6. Description of *Uthina saiyokensis* Yao & Li sp. nov…………………………...XXIII
Text S7. Description of *Uthina sarikaensis* Yao & Li sp. nov…………………………....XXV
Text S8. Description of *Uthina sulawesiensis* Yao & Li sp. nov…………………..…….XXV
Text S9. Description of *Uthina wongpromi* Yao & Li sp. nov…………………………...XXVI
Text S10. Description of *Uthina yunchuni* Yao & Li sp. nov……………………..…....XXVII
Text S11. Description of *Uthina zhigangi* Yao & Li sp. nov…………………………...XXVIII
Figures S6–S8. Illustrations of *Uthina huahinensis* Yao & Li sp. nov………….……..XXIX
Figures S9–S11. Illustrations of *Uthina huifengi* Yao & Li sp. nov……………………XXXII
Figure S12. Illustrations of *Uthina javaensis* Yao & Li sp. nov………………..…...XXXV
Figures S13–S15. Illustrations of *Uthina muangensis* Yao & Li sp. nov……………...XXXVI
Figures S16–S18. Illustrations of *Uthina potharamensis* Yao & Li sp. nov…………..XXXIX
Figures S19–S21. Illustrations of *Uthina saiyokensis* Yao & Li sp. nov………………..…XLII
Figure S22. Illustrations of *Uthina sarikaensis* Yao & Li sp. nov………………………...XLV
Figures S23–S25. Illustrations of *Uthina sulawesiensis* Yao & Li sp. nov……………....XLVI
Figures S26–S27. Illustrations of *Uthina wongpromi* Yao & Li sp. nov………………...XLIX
Figure S28. Illustrations of *Uthina yunchuni* Yao & Li sp. nov……………………....….LI
Figure S29. Illustrations of *Uthina zhigangi* Yao & Li sp. nov…………………………..…LII

Figure S1. Phylogenetic tree derived from the Bayesian analysis of the concatenated data. Bayesian posterior probabilities and bootstrap values from likelihood analysis are provided above and below branches respectively. Bootstrap values below 50 are not shown

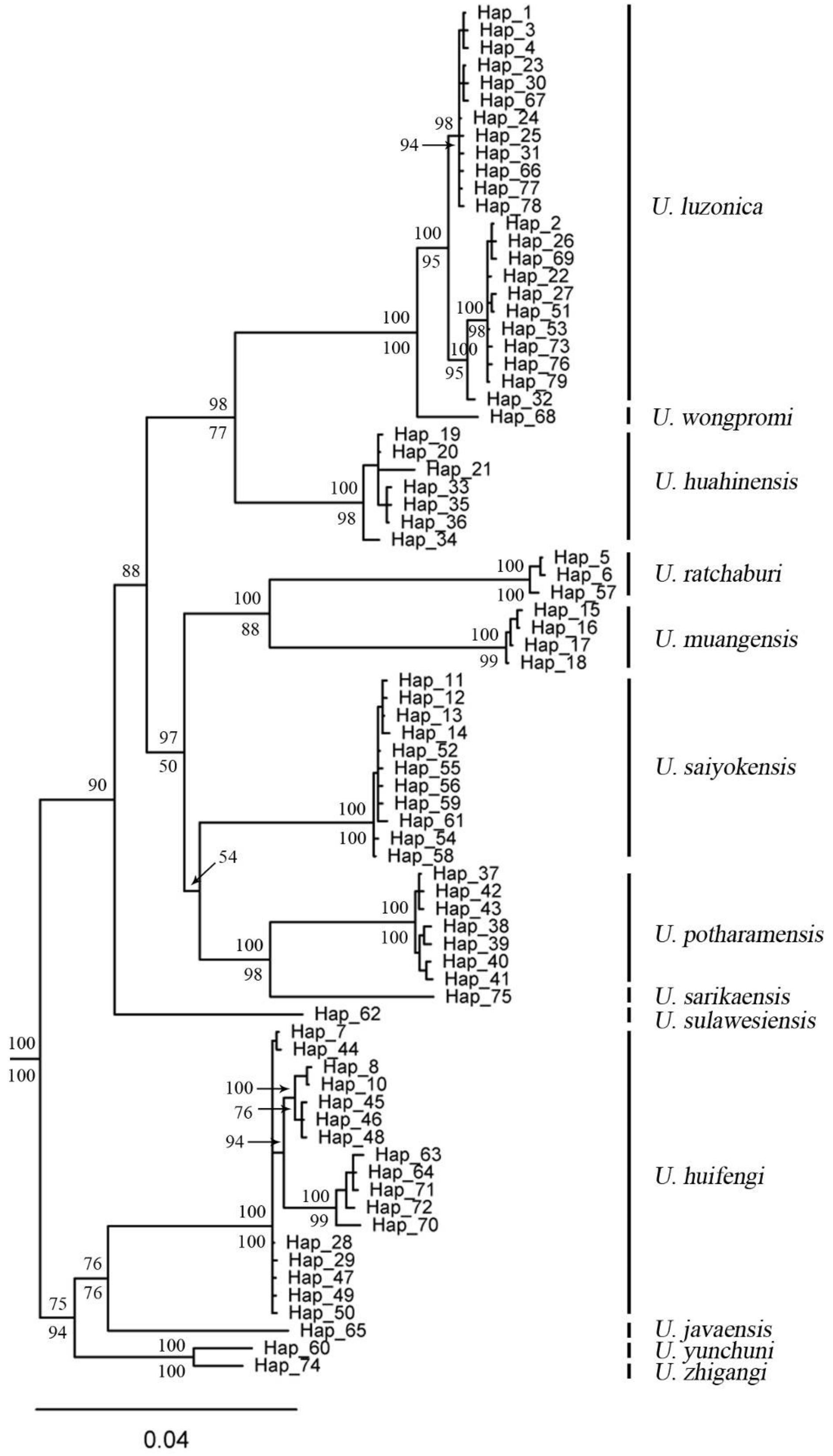

Figure S2. Individual gene trees. a, COI gene tree derived from the Bayesian analysis; b, clade *U.huifengi* of the COI gene tree derived from maximum likelihood analysis; c, 28S gene tree. Bayesian posterior probabilities and bootstrap values from the likelihood analysis are provided above and below branches respectively. Bootstrap values below 50 are not shown

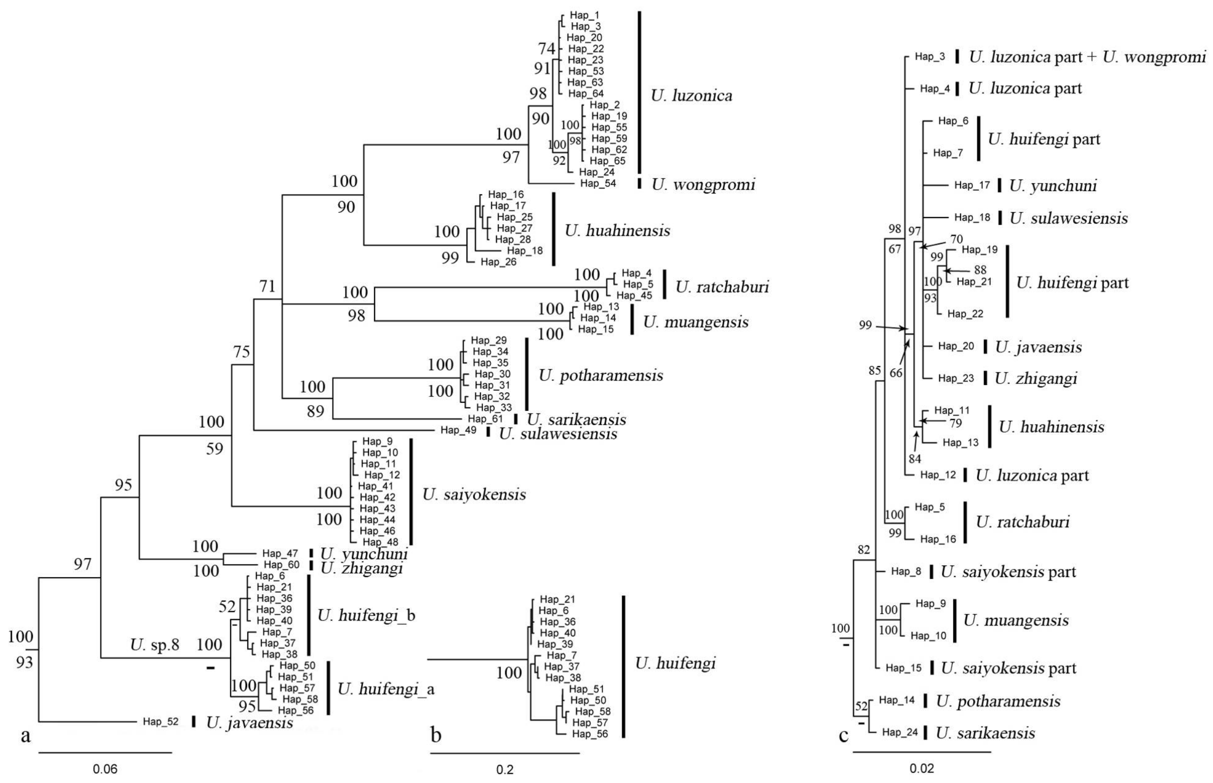

Figure S3. Results of bPTP species delimitation analysis based on COI gene tree derived from likelihood analysis (left) and Bayesian analysis (right). BS values are provided near the nodes

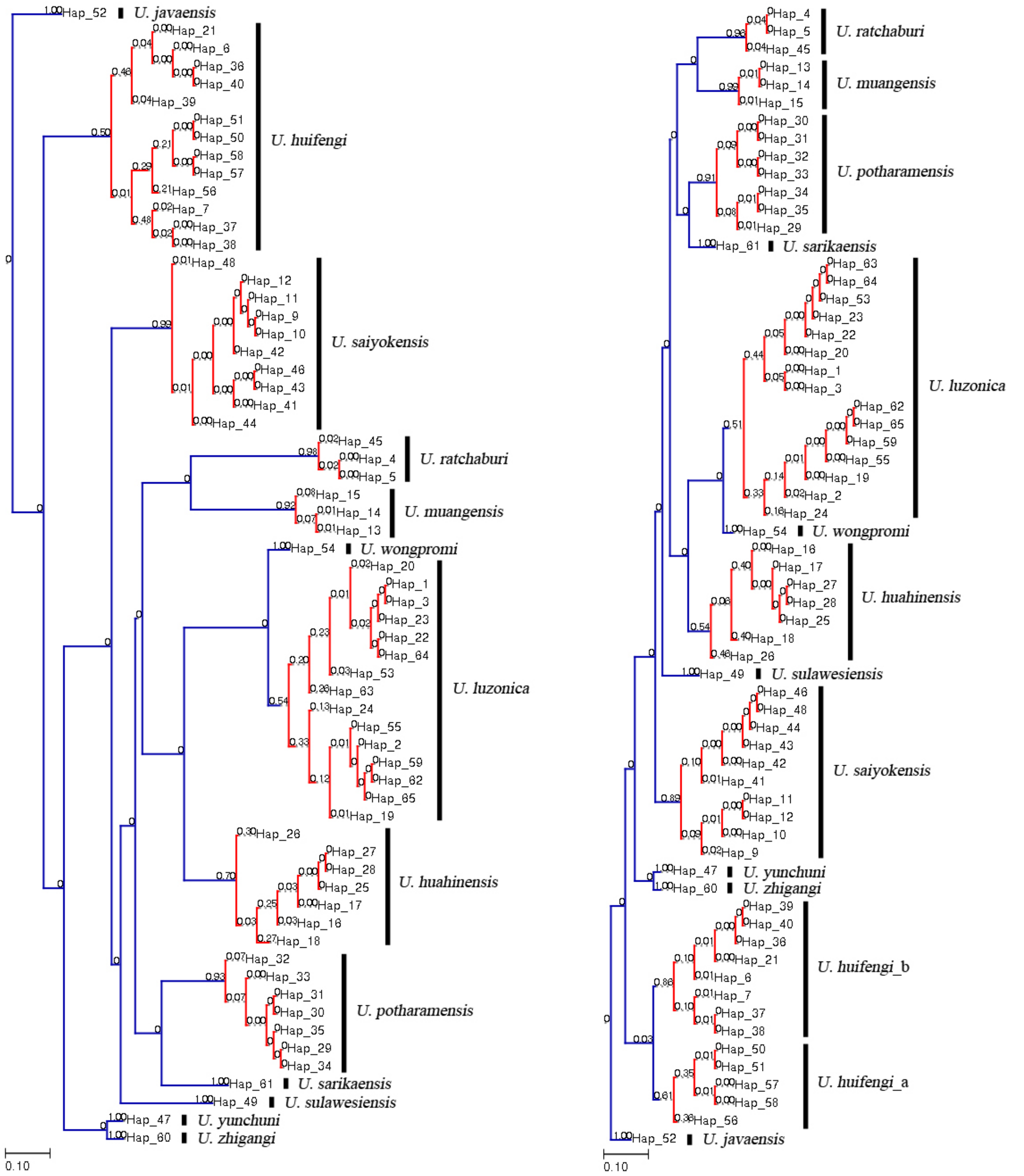

Figure S4. Results of bPTP species delimitation analysis based on 28S gene tree derived from Bayesian analysis (above) and likelihood analysis (below). BS values are provided near the nodes

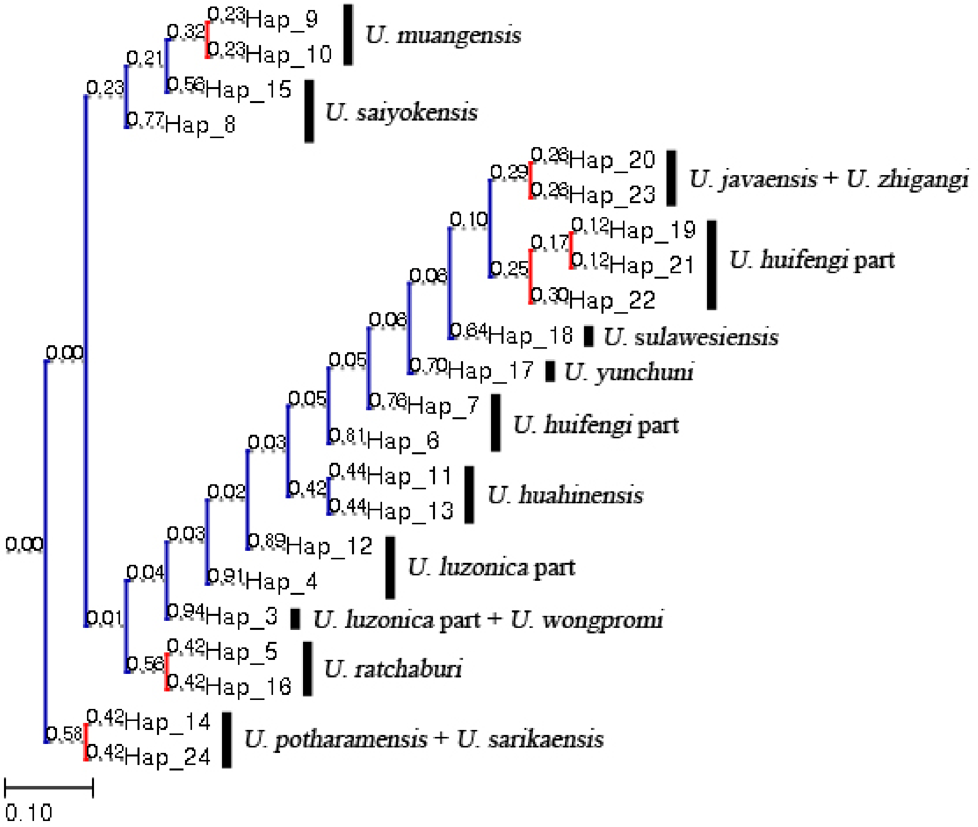

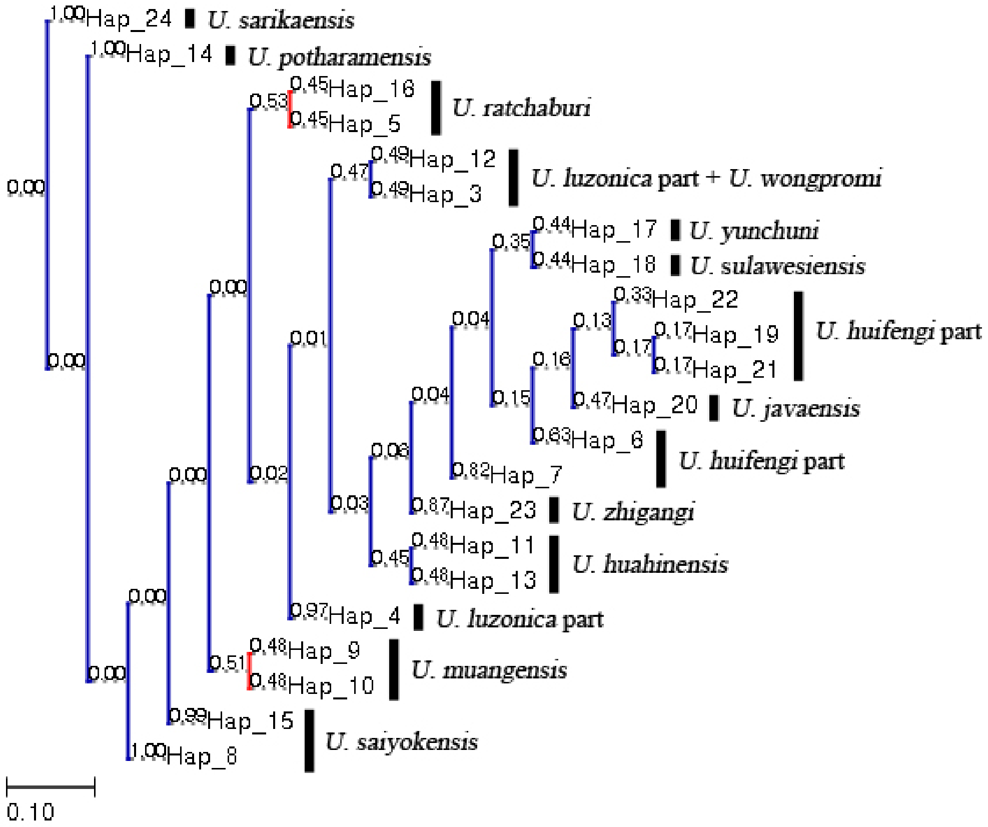

Figure S5. Results of GMYC species delimitation analysis based on COI gene tree. The blue line indicates the maximum-likelihood transition point where the branching rates switch from inter-specific to intra-specific events, as estimated by the GMYC model. Below are lineages-through-time plot (left) and single-threshold GMYC likelihood profile plot (right)

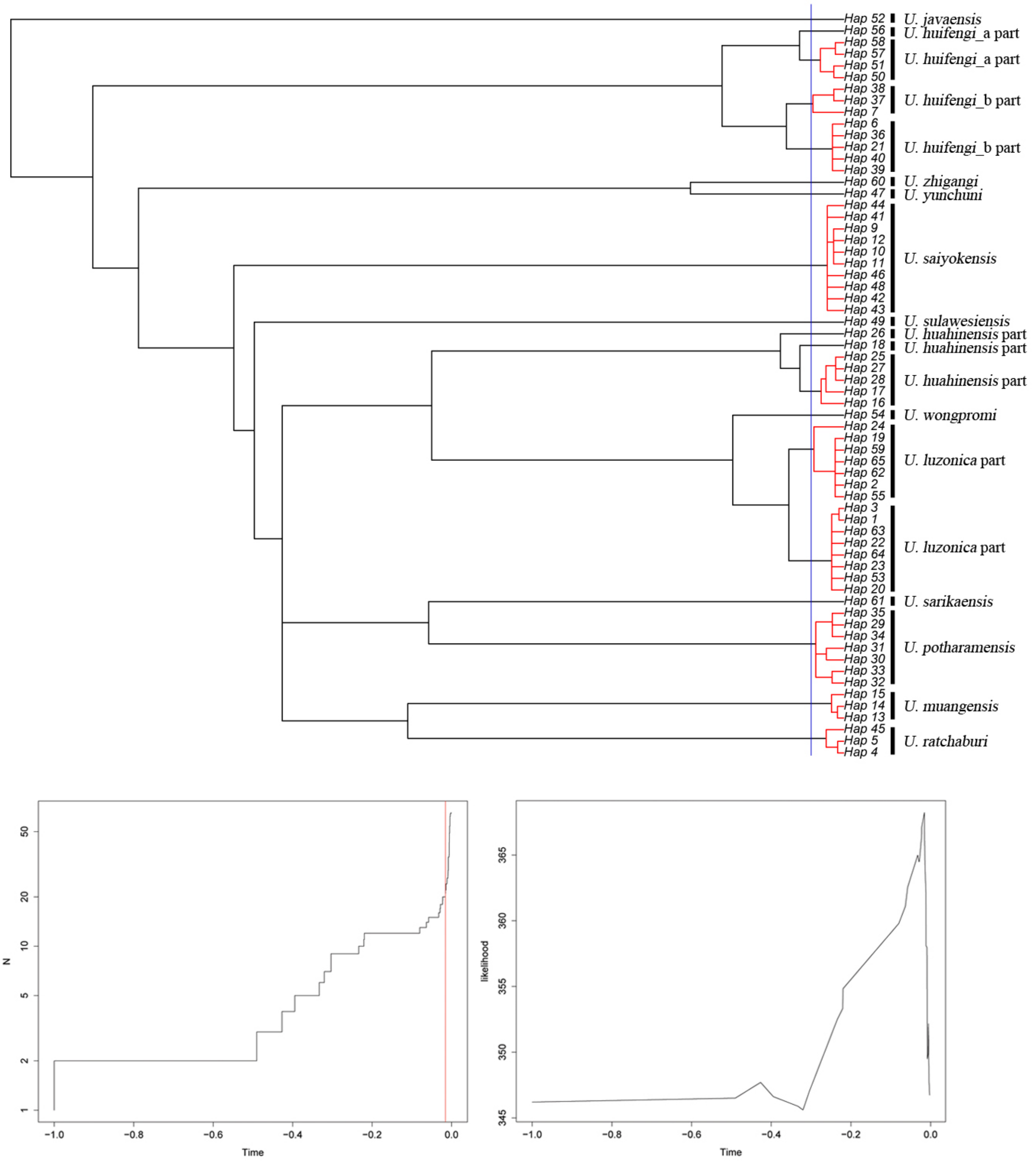

Table S1. Sampling localities and GenBank accession numbers

Species	Sample code	Site Number	Locality	COI	28S
U. luzonica	001_1	1	ID 003–004, H. Zhao, Z. Yao, 2014-VIII-12, Indonesia, Central Kalimantan, Tumbang Tahai, near Rungan Sari Hotel, 1º58.241'S, 113º44.712'E, elevation 3 m	KX980548	KX980764
	001_2			KX980547	KX980763
	001_3			KX980549	KX980765
	001_4			KX980550	KX980766
	001_5			KX980551	KX980767
	001_6			KX980552	KX980768
	001_7			KX980553	KX980769
	001_8			KX980554	KX980770
	001_9			KX980555	KX980771
	001_10			KX980556	KX980772
	019_1	2	ID 006–007, H. Zhao, Z. Yao, 2014-VIII-13, Indonesia, Central Kalimantan, Tumbang Tahai, Batu Banama, 1º59.312'S, 113º45.328'E, elevation 26 m	KX980697	KX980913
	019_2			KX980698	KX980914
	019_3			KX980699	KX980915
	019_4			KX980700	KX980916
	019_5			KX980701	KX980917
	035_1	3	ID 018–020, H. Zhao, Z. Yao, 2014-VIII-16, Indonesia, East Kalimantan, Penajam, Camp of International Timber Corp. of Indonesia, 1º5'17.49″S, 116º41'5.63″E, elevation 64 m	KX980746	KX980962
	007_1	4	Li-TW 008–009, S. Li, 2013-VI-26, China, Taiwan, Pingdong County, Kending, 21º57′25.15″N, 120º49′06.29″E, elevation 221 m	KX980607	KX980823
	007_2			KX980608	KX980824
	007_3			KX980609	KX980825
	007_4			KX980610	KX980826
	007_5			KX980611	KX980827
	007_6			KX980612	KX980828
	007_7			KX980613	KX980829
	007_8			KX980614	KX980830
	007_9			KX980615	KX980831
	007_10			KX980616	KX980832
	009_1	5	Li-TW 015–022, S. Li, 2013-VI-29, China, Taiwan, Gaoxiong, Shou Mountain, entrance of cave without a name, 22º39'N, 120º16'E	KX980627	KX980843
	009_2			KX980628	KX980844
	009_3			KX980629	KX980845
	009_4			KX980630	KX980846
	009_5			KX980631	KX980847
	009_6			KX980632	KX980848
	009_7			KX980633	KX980849
	009_8			KX980634	KX980850
	009_9			KX980635	KX980851
	009_10			KX980636	KX980852
	016_1	6	004-005, H. Zhao, 2013-XI-30, Forest face to Tea factory near Morne Blanc in Mahe, Seychelles, 4°39.724'S, 55°26.272'E, elevation 421 m	KX980682	KX980898
	016_2			KX980683	KX980899
	016_3			KX980684	KX980900
	016_4			KX980685	KX980901
	016_5			KX980686	KX980902
	041_1	7	H. Zhao, 2013-XII-03, Costal forest of Jardin Marron in Praslin, Seychelles, 4°17.929'S, 55°42.820'E, elevation 18 m	KX980752	KX980968
	042_1	8	H. Zhao, 2013-XII-07, Leaf litter in west costal forest in Mahe, Seychelles, 4°44.094'S,55°29.612'E, elevation 27 m	KX980753	KX980969

	017_1	9	SR 061–065, Z. Yao, Q. Zhao, 2014-X-7, Sri Lanka, Central Province, Kandy District, Peraderiya Town, Royal Botanic Gardens, 7º16'31.3″N, 80º35'42.7″E, elevation 484 m	KX980687	KX980903
	017_2			KX980688	KX980904
	017_3			KX980689	KX980905
	017_4			KX980690	KX980906
	017_5			KX980691	KX980907
	024_1	10	SR 010–011, Z. Yao, Q. Zhao, 2014-IX-25, Sri Lanka, Western Province, Colombo District, Maniyangama, Avissawella Town, Mahawafa Village, by the roadside, 6º57'26.0″N, 80º13'54.6″E, elevation 41 m	KX980722	KX980938
	024_2			KX980723	KX980939
	024_3			KX980724	KX980940
	032_1	11	SR 012–014, Z. Yao, Q. Zhao, 2014-IX-26, Sri Lanka, Western Province, Colombo District, Maniyangama, Avissawella Town, Mahawafa Village, Mahawafa Hill, 6º55'55.3″N, 80º14'40.6″E, elevation 38 m	KX980740	KX980956
	030_1	12	ID 057, H. Zhao, Z. Yao, 2014-VIII-22, Indonesia, South Sulawesi, Maros, Samanggi Village, Balang Ajia Orchard, near entrance of Cave 1 without a name, 5º3'2.76″S, 119º41'9.40″E, elevation 50 m	KX980737	KX980953
	030_2			KX980738	KX980954
	040_1	13	H. Zhao, 2014-I-15, Indonesia, Sumatra, North Sumatra Province, Lake Toba, Samosir island, 02°42.858' N, 98°48.312'E, elevation 934 m	KX980751	KX980967
	405_1	14	047–048, H. Zhao, Y. Li, 2015-IV-18, Fiji, Viti levu, Ba Province, Lautoka, Abaca vill., a forest in Koroyanitu National Heritage park, 17.57842ºS, 177.96129ºE, elevation 763 m	KX980754	KX980970
	405_2			KX980755	KX980971
	406_1	15	063–064, H. Zhao, Y. Li,2015-IV-21, Fiji, Viti levu,Suva, forest of Colo-i-suva forest park, 18.11344ºS, 178.33635ºE, elevation 26 m	KX980756	KX980972
	406_2			KX980757	KX980973
	407_1	16	Phi210, Huber B.A., 2014-II-28 Philippines, Luzon, near Olangapo, along river near Pamulaklakin Forest Trail, 14.7980°N, 121.3380°E	KX980758	KX980974
	408_1	17	Phi252, B.A. Huber, 2014-II-16, Philippines, Mindanao, Barangay San Jose, entrance of Blue Water Cave, 7.7060°N, 125.0320°E	KX980759	KX980975
U. wongpromi	031_2	18	056–058, H. Zhao, Y. Li, Z. Chen, 2014-X-17, Thailand, Phitsanulok Province, Nakhothai Dist, Ban Kaset Sut Vill., entrance of Tham Than La Cave, 16°54.816'N, 100°43.227'E	KX980739	KX980955
	034_1	19	066–067, H. Zhao, Y. Li, Z. Chen, 2014-X-18, Thailand, Phitsanulok Province, Nakhothai Dist,. Ban Tham Phrik Vill., entrance of Taraswan Cave, 16°55.220'N, 100°43.19'E, elevation 460m	KX980744	KX980960
	034_2			KX980745	KX980961
	039_1	20	130–139, 140–141, H. Zhao, Y. Li, Z. Chen, 2014-X-28, Thailand, Ranong Province, Kraburi Dist. entrance of Phra Kha Yang Cave, 10°19.568'N, 98°45.908'E, elevation 6 m	KX980750	KX980966
U. huahinensis	006_1	21	148–149, H. Zhao, Y. Li, Z. Chen, 2014-X-30, Thailand, Prachuap Kiri Khan Province, Hua Hin Dist. Nong Phlap Subdist., entrance of Dao Cave,	KX980597	KX980813
	006_2			KX980598	KX980814
	006_3			KX980599	KX980815
	006_4			KX980600	KX980816

	006_5		12°35.449'N, 99°43.692'E, elevation 123 m	KX980601	KX980817
	006_6			KX980602	KX980818
	006_7			KX980603	KX980819
	006_8			KX980604	KX980820
	006_9			KX980605	KX980821
	006_10			KX980606	KX980822
	010_1	22	150–154, H. Zhao, Y. Li, Z. Chen, 2014-X-30, Thailand, Prachuap Kiri Khan Province, Hua Hin Dist. Nong Phlap Subdist. entrances of Laplae Cave & Kailone Cave, 12°36.255'N, 99°43.341'E, elevation 175 m	KX980637	KX980853
	010_2			KX980638	KX980854
	010_3			KX980639	KX980855
	010_4			KX980640	KX980856
	010_5			KX980641	KX980857
	010_6			KX980642	KX980858
	010_7			KX980643	KX980859
	010_8			KX980644	KX980860
	010_9			KX980645	KX980861
	010_10			KX980646	KX980862
U. ratchaburi	002_1	23	087, H. Zhao, Y. Li, Z. Chen, 2014-X-21, Thailand, Ratchaburi Province, Muang Dist. Koh Phalapphla Vill., entrance of Tham Phraya Prap Cave, 13°35.018'N, 99°45.297'E	KX980557	KX980773
	002_2			KX980558	KX980774
	002_3			KX980559	KX980775
	002_4			KX980560	KX980776
	002_5			KX980561	KX980777
	002_6			KX980562	KX980778
	002_7			KX980563	KX980779
	002_8			KX980564	KX980780
	002_9			KX980565	KX980781
	002_10			KX980566	KX980782
	022_1	24	088–090, H. Zhao, Y. Li, Z. Chen, 2014-X-21, Thailand, Ratchaburi Province, Muang Dist., Koh Phalappha Vill., entrance of Tham Wat Takaeng Cave, 13°35.226'N, 99°45.525'E	KX980712	KX980928
	022_2			KX980713	KX980929
	022_3			KX980714	KX980930
	022_4			KX980715	KX980931
	022_5			KX980716	KX980932
U. muangensis	005_1	25	155–159, H. Zhao, Y. Li, Z. Chen, 2014-X-31, Thailand, Ratchaburi Province, Maung Dist. Thuang luang Subdist. entrance of Sawamg Cave, 13°28.014'N, 99°44.014'E, elevation 44 m	KX980587	KX980803
	005_2			KX980588	KX980804
	005_3			KX980589	KX980805
	005_4			KX980590	KX980806
	005_5			KX980591	KX980807
	005_6			KX980592	KX980808
	005_7			KX980593	KX980809
	005_8			KX980594	KX980810
	005_9			KX980595	KX980811
	005_10			KX980596	KX980812
U. saiyokensis	004_1	26	165–167, H. Zhao, Y. Li, Z. Chen, 2014-XI-2, Thailand, Kanchanaburi Province, Sai Yok Dist. Wang Krachae Subdist., entrance of cave without a name, 14°11.429'N, 99°01.594'E	KX980577	KX980793
	004_2			KX980578	KX980794
	004_3			KX980579	KX980795
	004_4			KX980580	KX980796
	004_5			KX980581	KX980797
	004_6			KX980582	KX980798
	004_7			KX980583	KX980799
	004_8			KX980584	KX980800
	004_9			KX980585	KX980801
	004_10			KX980586	KX980802
	018_1	27	168–170, H. Zhao, Y. Li, Z. Chen, 2014-XI-2, Thailand, Kanchanaburi Province, Sai Yok Dist. Wang Krachae Subdist. entrance of cave without a name, 14°11.400'N, 99°01.555'E	KX980692	KX980908
	018_2			KX980693	KX980909
	018_3			KX980694	KX980910
	018_4			KX980695	KX980911
	018_5			KX980696	KX980912

	021_1	28	162, H. Zhao, Y. Li, Z. Chen, 2014-XI-1, Thailand, Kanchanaburi Province, Sai Yok Dist. Wang Krachae Subdist., entrance of cave without a name, 14°12.219' N, 99°01.666'E, elevation 325 m	KX980707	KX980923
	021_2			KX980708	KX980924
	021_3			KX980709	KX980925
	021_4			KX980710	KX980926
	021_5			KX980711	KX980927
	023_1	29	164, H. Zhao, Y. Li, Z. Chen, 2014-XI-1, Thailand, Kanchanaburi Province, Sai Yok Dist. Wang Krachae Subdist., entrance of cave without a name, 14°12.127'N, 99°01.195'E, elevation 438 m	KX980717	KX980933
	023_2			KX980718	KX980934
	023_3			KX980719	KX980935
	023_4			KX980720	KX980936
	023_5			KX980721	KX980937
	026_1	30	163, H. Zhao, Y. Li, Z. Chen, 2014-XI-1, Thailand, Kanchanaburi Province, Sai Yok Dist. Wang Krachae Subdist., entrance of cave without a name, 14°12.182'N, 99°01.416'E, elevation 342 m	KX980727	KX980943
	026_2			KX980728	KX980944
	026_3			KX980729	KX980945
U. potharamensis	011_1	31	082, H. Zhao, Y. Li, Z. Chen, 2014-X-21, Thailand, Ratchaburi Province, Potharam Dist. Nang Kaeo Subdist., entrance of Tham Nam Cave, 13°41.860'N, 99°45.415'E	KX980647	KX980863
	011_2			KX980648	KX980864
	011_3			KX980649	KX980865
	011_4			KX980650	KX980866
	011_5			KX980651	KX980867
	011_6			KX980652	KX980868
	011_7			KX980653	KX980869
	011_8			KX980654	KX980870
	011_9			KX980655	KX980871
	011_10			KX980656	KX980872
U. sarikaensis	038_1	32	083–086, H. Zhao, Y. Li, Z. Chen, 2014-X-21, Thailand, Ratchaburi Province, Muang Dist.,Tham Sen Subdist., entrance of Tham Sarika Cave, 13°29.773' N, 99°44.229'E, elevation 48 m	KX980749	KX980965
U. sulawesiensis	027_1	33	ID 063–064, H. Zhao, Z. Yao, 2014-VIII-23, Indonesia, South Sulawesi, Maros, entrance of cave without a name, 4º52.344'S, 119º53.858'E, elevation 417 m	KX980730	KX980946
	027_2			KX980731	KX980947
	036_1	34	ID 060, H. Zhao, Z. Yao, 2014-VIII-23, Indonesia, South Sulawesi, Maros, Cenrana Village, Benteng Orchard, entrance of Gua (= Cave) Liang Gose, 04°56.304′S, 119°50.341′E, elevation 355 m	KX980747	KX980963
U. huifengi	003_1	35	Yao-ID 038–041, Z. Yao, 2014-V-18, Indonesia, Sumatra, West Sumatra Province, Bukittinggi, entrance of Kalam Cave, 00º12.275'S, 100º23.475' E, elevation 874 m	KX980567	KX980783
	003_2			KX980568	KX980784
	003_3			KX980569	KX980785
	003_5			KX980571	KX980787
	003_6			KX980572	KX980788
	003_7			KX980573	KX980789
	003_8			KX980574	KX980790
	003_9			KX980575	KX980791
	003_10			KX980576	KX980792
	008_1	36	H. Zhao, 2014-I-12, Indonesia, Sumatra, West Sumatra Province, Kab Agam Kecamatan Matur, Kanagarian Matuailia, entrance of Batang lawang Cave, 0°15.739'S, 100°18.491'E, elevation 761m	KX980617	KX980833
	008_2			KX980618	KX980834
	008_3			KX980619	KX980835
	008_4			KX980620	KX980836
	008_5			KX980621	KX980837
	008_6			KX980622	KX980838
	008_7			KX980623	KX980839
	008_8			KX980624	KX980840

	008_9			KX980625	KX980841
	008_10			KX980626	KX980842
	012_1	37	H. Zhao, 2014-I-10, Indonesia, Sumatra, West Sumatra Province, Bukitinggi, Fort de Kock, entrance of cave without a name, 0°17.982'S, 100°22.090'E, elevation 940 m	KX980657	KX980873
	012_2			KX980658	KX980874
	012_3			KX980659	KX980875
	012_4			KX980660	KX980876
	012_5			KX980661	KX980877
	012_6			KX980662	KX980878
	012_7			KX980663	KX980879
	012_8			KX980664	KX980880
	012_9			KX980665	KX980881
	012_10			KX980666	KX980882
	013_1	38	H. Zhao, 2014-I-11, Indonesia, Sumatra, West Sumatra Province, Kab Agam TaBik Simarasok Village, entrance of Jorong Koto Tuo Cave, 0°14.901'S, 100°28.992'E, elevation 714 m	KX980667	KX980883
	013_2			KX980668	KX980884
	013_3			KX980669	KX980885
	013_4			KX980670	KX980886
	013_5			KX980671	KX980887
	014_1	39	Yao-ID 012–016, Z. Yao, 2014-V-12, Indonesia, Sumatra, West Sumatra Province, Bukittinggi, entrance of Tarang Cave, 00º12.653'S, 100º24.977'E, elevation 877 m	KX980672	KX980888
	014_2			KX980673	KX980889
	014_3			KX980674	KX980890
	014_4			KX980675	KX980891
	014_5			KX980676	KX980892
	015_1	40	H. Zhao, 2014-I-12, Indonesia, Sumatra, West Sumatra Province, Bukitinggi, entrance of Jenjang Seribu Cave, 0°18.104'S, 100°21.335'E, elevation 815 m	KX980677	KX980893
	015_2			KX980678	KX980894
	015_3			KX980679	KX980895
	015_4			KX980680	KX980896
	015_5			KX980681	KX980897
	020_1	41	H. Zhao, 2014-I-11, Indonesia, Sumatra, West Sumatra Province, Bukitinggi, entrance of Japanese Cave, 0°18.458'S, 100°21.933'E, elevation 906 m	KX980702	KX980918
	020_2			KX980703	KX980919
	020_3			KX980704	KX980920
	020_4			KX980705	KX980921
	020_5			KX980706	KX980922
	028_1	42	Yao-ID 061–065, Z. Yao, 2014-V-27, Indonesia, Sumatra, West Sumatra Province, Sijunjung, Padang Sibusuk Village, entrance of Bukit Ponggang Cave, 00º44.245'S, 100º50.330'E, elevation 278 m	KX980732	KX980948
	028_2			KX980733	KX980949
	028_3			KX980734	KX980950
	033_1	43	Yao-ID 052–054, Z. Yao, 2014-V-24, Indonesia, Sumatra, Jambi Province, Kerinci, Talang Cindang, near river in a village, 02º04.834'S, 101º22.448'E, elevation 1054 m	KX980741	KX980957
	033_2			KX980742	KX980958
	033_3			KX980743	KX980959
U. javaensis	029_1	44	ID 072–075, H. Zhao, Z. Yao, 2014-VIII-27, Indonesia, Java, Bantul, Mangunan Village, entrance of Gua (= Cave) Gajah, 07°56.662′S, 110°26.404′E, elevation 234 m	KX980735	KX980951
	029_2			KX980736	KX980952
U. yunchuni	025_1	45	108–110, H. Zhao, Y. Li, Z. Chen, 2014-X-25, Thailand, Krabi Province, Muang Dist., Ban Chong Plee Vill., entrance of cave without a name, 08°05.122′N, 98°51.223′E, elevation 442 m	KX980725	KX980941
	025_2			KX980726	KX980942
U. zhigangi	037_1	46	116–117, H. Zhao, Y. Li, Z. Chen, 2014-X-26, Thailand, Krabi Province, Muang Dist. Ban Khao Klom Nong Thale Subdist., entrance of cave without a name, 08°08.155'N, 98°48.430'E, elevation 89 m	KX980748	KX980964
Pholcus jiuwei	outgroup	Tong Y, 2014-IX-7, China, Liaoning, Benxi,		KX980760	KX980976

Tong & Ji, 2010		Pingshan District, Xing'an Village, 41°15'N, 123°42'E		
Pholcus phoenixus Zhang & Zhu, 2009	outgroup	Q. Zhao, 2014-VIII-28, China, Liaoning, Fenghuang, Fenghuang Mountain, 40°25'38.2"N, 124°4'7.5" E, elevation 149–530 m	KX980761	KX980977
Leptopholcus podophthalmus (Simon, 1893)	outgroup	H. Zhao, L. Shao, 2014-XII-16, China, Hainan, Dongfang, Donghe Town, Nanglang Village, Jiulong Mountain, 19°0.652'N, 109°5.623'E, elevation 522 m	KX980762	N/A

Table S2. List of cave clusters involved

Cave cluster	Site Number	Coordinate
Cave cluster 1	18	N: 16°54.816', E: 100°43.227'
	19	N: 16°55.220', E: 100°43.19'
Cave cluster 2	21	N: 12°35.449', E: 99°43.692'
	22	N: 12°36.255', E: 99°43.341'
Cave cluster 3	23	N: 13°35.018', E: 99°45.297'
	24	N: 13°35.226', E: 99°45.525'
Cave cluster 4	26	N: 14°11.429', E: 99°01.594'
	27	N: 14°11.400', E: 99°01.555'
	28	N: 14°12.219', E: 99°01.666'
	29	N: 14°12.127', E: 99°01.195'
	30	N: 14°12.182', E: 99°01.416'
Cave cluster 5	33	S: 4º52.344', E: 119º53.858'
	34	S: 4º56.304', E: 119º50.341'
Cave cluster 6	35	S: 0º12.275', E:100º23.475'
	39	S: 0º12.653', E:100º24.977'
	38	S: 0°14.901' E: 100°28.992'
	36	S: 0°15.739' E: 100°18.491'
	37	S: 0°17.982’ E: 100°22.090'
	40	S: 0°18.104' E: 100°21.335'
	41	S: 0°18.458' E: 100°21.933'

Table S3. List of haplotypes and samples that share the same haplotype

COI	Freq.	Samples
Hap_1	9	001_2, 001_5, 001_6, 001_7, 001_8, 001_9, 001_10, 019_3, 019_5
Hap_2	8	001_1, 017_3, 019_1, 019_2, 019_4, 024_1, 024_2, 024_3
Hap_3	2	001_3, 001_4
Hap_4	9	002_1, 002_2, 002_3, 002_4, 002_5, 002_7, 002_8, 002_9, 002_10
Hap_5	1	002_6
Hap_6	21	003_1, 003_5, 003_6, 003_8, 008_1, 008_2, 008_3, 008_4, 008_5, 008_6, 008_7, 008_8, 008_9, 013_4, 014_3, 014_5, 015_5, 020_1, 020_3, 020_4, 020_5
Hap_7	8	003_2, 003_3, 003_7, 003_9, 003_10, 014_1, 014_2, 014_4
Hap_9	4	004_1, 004_2, 004_5, 004_7
Hap_10	1	004_3
Hap_11	8	004_4, 004_8, 004_9, 004_10, 018_1, 018_2, 018_3, 018_4
Hap_12	1	004_6
Hap_13	7	005_1, 005_2, 005_5, 005_6, 005_8, 005_9, 005_10
Hap_14	2	005_3, 005_4
Hap_15	1	005_7
Hap_16	5	006_1, 006_2, 006_5, 006_6, 006_8
Hap_17	4	006_3, 006_4, 006_7, 006_9
Hap_18	1	006_10
Hap_19	3	007_1, 007_7, 007_10
Hap_20	26	007_2, 007_3, 007_4, 007_5, 007_6, 007_8, 007_9, 009_2, 009_5, 009_7, 009_8, 009_9, 009_10, 016_1, 016_2, 016_3, 016_4, 016_5, 017_1, 017_2, 017_4, 017_5, 041_1, 042_1, 405_1, 406_1
Hap_21	1	008_10
Hap_22	2	009_1, 009_4
Hap_23	1	009_3
Hap_24	1	009_6
Hap_25	3	010_1, 010_3, 010_6
Hap_26	1	010_2
Hap_27	1	010_4
Hap_28	5	010_5, 010_7, 010_8, 010_9, 010_10
Hap_29	3	011_1, 011_7, 011_8
Hap_30	1	011_2
Hap_31	2	011_3, 011_4
Hap_32	1	011_5
Hap_33	1	011_6
Hap_34	1	011_9
Hap_35	1	011_10
Hap_36	3	012_1, 012_7, 012_10
Hap_37	11	012_2, 012_3, 012_4, 012_5, 012_6, 012_8, 012_9, 015_1, 015_2, 015_3, 020_2
Hap_38	3	013_1, 013_3, 013_5
Hap_39	1	013_2
Hap_40	1	015_4
Hap_41	5	018_5, 023_3, 023_4, 026_1, 026_3
Hap_42	1	021_1
Hap_43	3	021_2, 021_3, 021_4
Hap_44	3	021_5, 023_1, 023_2
Hap_45	5	022_1, 022_2, 022_3, 022_4, 022_5
Hap_46	1	023_5
Hap_47	2	025_1, 025_2
Hap_48	1	026_2
Hap_49	3	027_1, 027_2, 036_1
Hap_50	2	028_1, 028_3
Hap_51	1	028_2
Hap_52	2	029_1, 029_2
Hap_53	2	030_1, 030_2

Hap_54	4	031_2, 034_1, 034_2, 039_1
Hap_55	1	032_1
Hap_56	1	033_1
Hap_57	1	033_2
Hap_58	1	033_3
Hap_59	1	035_1
Hap_60	1	037_1
Hap_61	1	038_1
Hap_62	1	040_1
Hap_63	2	405_2, 406_2
Hap_64	1	407_1
Hap_65	1	408_1

**28S**	**Freq.**	**Samples**
Hap_3	38	001_2, 001_3, 001_4, 001_5, 001_7, 001_8, 001_10, 007_1, 007_3, 007_4, 007_8, 007_9, 009_2, 009_3, 009_6, 009_7, 009_8, 009_9, 016_2, 019_3, 019_4, 024_1, 024_2, 024_3, 030_1, 031_2, 034_1, 034_2, 035_1, 039_1, 040_1, 042_1, 405_1, 405_2, 406_1, 406_2, 407_1, 408_1
Hap_4	19	001_1, 001_6, 001_9, 007_5, 007_6, 007_7, 009_10, 016_1, 016_3, 016_4, 016_5, 017_1, 017_4, 017_5, 019_1, 019_2, 019_5, 032_1, 041_1
Hap_5	10	002_1, 002_2, 002_3, 002_4, 002_5, 002_6, 002_7, 002_8, 002_9, 002_10
Hap_6	14	003_1, 003_2, 003_3, 003_5, 003_6, 003_7, 003_8, 003_10, 012_1, 012_2, 012_8, 014_2, 015_2, 020_4
Hap_7	37	003_9, 008_1, 008_2, 008_3, 008_4, 008_5, 008_6, 008_7, 008_8, 008_9, 008_10, 012_3, 012_4, 012_5, 012_6, 012_7, 012_9, 012_10, 013_1, 013_2, 013_3, 013_4, 013_5, 014_1, 014_3, 014_4, 014_5, 015_1, 015_3, 015_4, 015_5, 020_1, 020_2, 020_3, 020_5, 028_1, 028_3
Hap_8	25	004_1, 004_2, 004_3, 004_4, 004_5, 004_6, 004_7, 004_8, 004_9, 004_10, 018_1, 018_2, 018_3, 018_4, 018_5, 021_2, 021_3, 021_4, 021_5, 023_1, 023_2, 023_5, 026_1, 026_2, 026_3
Hap_9	1	005_1
Hap_10	9	005_2, 005_3, 005_4, 005_5, 005_6, 005_7, 005_8, 005_9, 005_10
Hap_11	10	006_1, 006_2, 006_3, 006_4, 006_5, 006_6, 006_7, 006_8, 006_9, 006_10
Hap_12	8	007_2, 007_10, 009_1, 009_4, 009_5, 017_2, 017_3, 030_2
Hap_13	10	010_1, 010_2, 010_3, 010_4, 010_5, 010_6, 010_7, 010_8, 010_9, 010_10
Hap_14	10	011_1, 011_2, 011_3, 011_4, 011_5, 011_6, 011_7, 011_8, 011_9, 011_10
Hap_15	3	021_1, 023_3, 023_4
Hap_16	5	022_1, 022_2, 022_3, 022_4, 022_5
Hap_17	2	025_1, 025_2
Hap_18	3	027_1, 027_2, 036_1
Hap_19	2	028_2, 033_2
Hap_20	2	029_1, 029_2
Hap_21	1	033_1
Hap_22	1	033_3
Hap_23	1	037_1
Hap_24	1	038_1

**COI_28S**	**Freq.**	**Samples**
Hap_1	6	001_2, 001_5, 001_7, 001_8, 001_10, 019_3
Hap_2	3	001_1, 019_1, 019_2
Hap_3	2	001_3, 001_4
Hap_4	3	001_6, 001_9, 019_5
Hap_5	9	002_1, 002_2, 002_3, 002_4, 002_5, 002_7, 002_8, 002_9, 002_10
Hap_6	1	002_6
Hap_7	5	003_1, 003_5, 003_6, 003_8, 020_4
Hap_8	5	003_2, 003_3, 003_7, 003_10, 014_2

Hap_10	3	003_9, 014_1, 014_4
Hap_11	4	004_1, 004_2, 004_5, 004_7
Hap_12	1	004_3
Hap_13	8	004_4, 004_8, 004_9, 004_10, 018_1, 018_2, 018_3, 018_4
Hap_14	1	004_6
Hap_15	1	005_1
Hap_16	6	005_2, 005_5, 005_6, 005_8, 005_9, 005_10
Hap_17	2	005_3, 005_4
Hap_18	1	005_7
Hap_19	5	006_1, 006_2, 006_5, 006_6, 006_8
Hap_20	4	006_3, 006_4, 006_7, 006_9
Hap_21	1	006_10
Hap_22	1	007_1
Hap_23	3	007_2, 009_5, 017_2
Hap_24	12	007_3, 007_4, 007_8, 007_9, 009_2, 009_7, 009_8, 009_9, 016_2, 042_1, 405_1, 406_1
Hap_25	11	007_5, 007_6, 009_10, 016_1, 016_3, 016_4, 016_5, 017_1, 017_4, 017_5, 041_1
Hap_26	1	007_7
Hap_27	1	007_10
Hap_28	16	008_1, 008_2, 008_3, 008_4, 008_5, 008_6, 008_7, 008_8, 008_9, 013_4, 014_3, 014_5, 015_5, 020_1, 020_3, 020_5
Hap_29	1	008_10
Hap_30	2	009_1, 009_4
Hap_31	1	009_3
Hap_32	1	009_6
Hap_33	3	010_1, 010_3, 010_6
Hap_34	1	010_2
Hap_35	1	010_4
Hap_36	5	010_5, 010_7, 010_8, 010_9, 010_10
Hap_37	3	011_1, 011_7, 011_8
Hap_38	1	011_2
Hap_39	2	011_3, 011_4
Hap_40	1	011_5
Hap_41	1	011_6
Hap_42	1	011_9
Hap_43	1	011_10
Hap_44	1	012_1
Hap_45	3	012_2, 012_8, 015_2
Hap_46	8	012_3, 012_4, 012_5, 012_6, 012_9, 015_1, 015_3, 020_2
Hap_47	2	012_7, 012_10
Hap_48	3	013_1, 013_3, 013_5
Hap_49	1	013_2
Hap_50	1	015_4
Hap_51	1	017_3
Hap_52	3	018_5, 026_1, 026_3
Hap_53	4	019_4, 024_1, 024_2, 024_3
Hap_54	1	021_1
Hap_55	3	021_2, 021_3, 021_4
Hap_56	3	021_5, 023_1, 023_2
Hap_57	5	022_1, 022_2, 022_3, 022_4, 022_5
Hap_58	2	023_3, 023_4
Hap_59	1	023_5
Hap_60	2	025_1, 025_2
Hap_61	1	026_2
Hap_62	3	027_1, 027_2, 036_1
Hap_63	2	028_1, 028_3
Hap_64	1	028_2
Hap_65	2	029_1, 029_2
Hap_66	1	030_1
Hap_67	1	030_2

Hap_68	4	031_2, 034_1, 034_2, 039_1
Hap_69	1	032_1
Hap_70	1	033_1
Hap_71	1	033_2
Hap_72	1	033_3
Hap_73	1	035_1
Hap_74	1	037_1
Hap_75	1	038_1
Hap_76	1	040_1
Hap_77	2	405_2, 406_2
Hap_78	1	407_1
Hap_79	1	408_1

Text S1. Description of *Uthina huahinensis* Yao & Li sp. nov.
urn:lsid:zoobank.org:act:3CE79C7A-9273-4E4B-8B00-FB60E7FEF0AF
Figs S6–S8

Material examined. Holotype: male, Dao Cave (12°35.449′N, 99°43.692′E, elevation 123 m), Nong Phlap, Hua Hin District, Prachuap Kiri Khan, Thailand, 30 October 2014, H. Zhao, Y. Li and Z. Chen leg. Paratypes: one male and two females, same data as holotype.

Other material. One male, two females and seven juveniles, same data as holotype. One male, seven females and two juveniles, Laplae Cave and Kailone Cave (12°36.255′N, 99°43.341′E, elevation 175 m), Nong Phlap, Hua Hin District, Prachuap Kiri Khan, Thailand, 30 October 2014, H. Zhao, Y. Li and Z. Chen leg.

Etymology. The specific name refers to the type locality; adjective.

Diagnosis. This species resembles *U. huifengi* sp. nov., in having similar male chelicerae (Fig. S7D), uncus (Fig. S7A), and female external genitalia (Fig. S8A), but can be distinguished by distally bifurcated appendix (Fig. S7A), by small angular apophysis close to 'knee' of procursus (arrow in Fig. S6D), and by different shape of vulval pore plates (anterior part wide but posterior part narrow, Fig. S8B).

Description. Male (holotype): Total length 4.09 (4.29 with clypeus), carapace 1.09 long, 1.33 wide, opisthosoma 3.00 long, 0.98 wide. Leg I: 43.15 (10.38 + 0.53 + 10.64 + 19.10 + 2.50), leg II: 26.60 (7.05 + 0.52 + 6.73 +10.90 + 1.40), leg III: 17.32 (4.94 + 0.48 + 4.42 + 6.60 + 0.88), leg IV: 22.36 (6.86 + 0.50 + 6.03 + 8.97 + 1.13); tibia I L/d: 97. Distance PME-PME 0.32; diameter PME 0.08; distance PME-ALE 0.02; AME absent. Sternum wider than long (0.84/0.70). Habitus as in Fig. S7B. Carapace yellowish, with large brown marks extending to ocular area; ocular area yellowish; sternum brown. Legs yellowish, distal parts of femora and tibiae whitish, darker rings absent. Opisthosoma yellowish, with spots posteriorly. Ocular area elevated; each eye triad on top of a short, laterally directed eye-stalk. Thoracic furrow absent. Chelicerae as in Fig. S7D, with a pair of proximo-lateral apophyses and a pair of distal apophyses. Pedipalps as in Figs S6A–B; trochanter with a short retrolatero-ventral apophysis; femur with a dorsal apophysis proximally; procursus simple proximally but complex distally; uncus strongly curved, with scales and a thin distal transparent process; appendix hooked, bifurcated distally, with scales; embolus weakly sclerotized, with some transparent projections distally. Retrolateral trichobothrium of tibia I at 4.9%; legs with short vertical setae on metatarsi and tarsi, without spines and curved setae; tarsus I with more than 20 distinct pseudosegments.

Female: Similar to male, habitus as in Fig. S7C. Total length 4.57 (4.87 with clypeus), carapace 1.17 long, 1.20 wide, opisthosoma 3.40 long, 1.13 wide; tibia I: 10.45; tibia I L/d: 82. Distance PME-PME 0.24; diameter PME 0.09; distance PME-ALE 0.02; AME absent. Sternum wider than long (0.84/0.72). Opisthosoma without spots. Ocular area brown, without eye-stalks. External genitalia (Fig. S8A) with a weakly sclerotized knob. Vulva (Fig. S8B) with an anterior arch and two nearly semicircular pore plates.

Variation: Unknown. Leg I missing in other specimens.

Distribution. Thailand (Prachuap Kiri Khan).

Natural history. The species was found in the entrance zones of caves.

Text S2. Description of *Uthina huifengi* Yao & Li sp. nov.
urn:lsid:zoobank.org:act:C81D62D1-EE65-421A-B4DD-3515E75D394E
Figs S9–S11

Material examined. Holotype: male, Kalam Cave (0°12.275′S, 100°23.475′E, elevation 874 m), Bukittinggi, West Sumatra, Sumatra, Indonesia, 18 May 2014, Z. Yao leg. Paratypes: two males and three females, same data as holotype.

Other material. Two males and eight females, same data as holotype. Two males, two females and one juvenile, Tarang Cave (0º12.653′S, 100º24.977′E, elevation 877 m), Bukittinggi, West Sumatra, Sumatra, Indonesia, 12 May 2014, Z. Yao leg. One male and two juveniles, near river in a village (2º04.834′S, 101º22.448′E, elevation 1054 m), Talang Cindang, Kerinci, Jambi, Sumatra, Indonesia, 24 May 2014, Z. Yao leg. One female and two juveniles, Bukit Ponggang Cave (0º44.245′S, 100º50.330′E, elevation 278 m), Padang Sibusuk Village, Sijunjung, West Sumatra, Sumatra, Indonesia, 27 May 2014, Z. Yao leg. One male, four females and five juveniles, Cave without a name (0°17.982′S, 100°22.090′E, elevation 940 m), Fort de Kock, Bukittinggi, West Sumatra, Sumatra, Indonesia, 10 January 2014, H. Zhao leg. One male, three females and one juvenile, Jorong Koto Tuo Cave (0°14.901′S, 100°28.992′E, elevation 714 m), Kab Agam Tabik Simarasok Village, West Sumatra, Sumatra, Indonesia, 11 January 2014, H. Zhao leg. Two females and three juveniles, Japanese Cave (0°18.458′S, 100°21.933′E, elevation 906 m), Bukittinggi, West Sumatra, Sumatra, Indonesia, 11 January 2014, H. Zhao leg. One male, seven females and two juveniles, Batang Lawang Cave (0°15.739′S, 100°18.491′E, elevation 761 m), Kanagarian Matuailia, Kab Agam Kecamatan Matur, West Sumatra, Sumatra, Indonesia, 12 January 2014, H. Zhao leg. Two females and three juveniles, Jenjang Seribu Cave (0°18.104′S, 100°21.335′E, elevation 815 m), Bukittinggi, West Sumatra, Sumatra, Indonesia, 12 January 2014, H. Zhao leg.

Etymology. The specific name is a patronym in honor of the collector Huifeng Zhao; noun (name) in genitive case.

Diagnosis. This species resembles *U. huahinensis* sp. nov., in having similar male chelicerae (Fig. S10D), uncus (Fig. S10A), and female external genitalia (Fig. S11A), but can be distinguished by appendix not bifurcated distally (Fig. S10A), by small angular apophysis close to large weakly sclerotized apophysis of procursus (arrow in Fig. S9D), and by different shape of vulval pore plates (anterior part narrow but posterior part wide, Fig. S11B).

Description. Male (holotype): Total length 3.92 (4.00 with clypeus), carapace 1.02 long, 1.20 wide, opisthosoma 2.72 long, 0.86 wide. Leg I: 37.44 (8.91 + 0.52 + 9.10 + 16.73 + 2.18), leg II: 22.53 (5.96 + 0.53 + 5.71 +9.23 + 1.10), leg III: 14.83 (4.23 + 0.44 + 3.66 + 5.64 + 0.86), leg IV missing; tibia I L/d: 84. Distance PME-PME 0.35; diameter PME 0.08; distance PME-ALE 033; AME absent. Sternum wider than long (0.68/0.60). Habitus as in Fig. S10B. Carapace yellowish, with large brown marks extending to ocular area; ocular area yellowish; sternum brown. Legs yellowish, subdistal parts of tibiae whitish, darker rings absent. Opisthosoma pale grey dorsally but yellowish ventrally, with spots dorsally and laterally. Ocular area elevated; each eye triad on top of a short, laterally directed eye-stalk. Thoracic furrow absent. Chelicerae as in Fig. S10D, with a pair of proximo-lateral apophyses and a pair of distal apophyses. Pedipalps as in Figs S9A–B; trochanter with a short

retrolatero-ventral apophysis; femur with a dorsal apophysis proximally; procursus simple proximally but complex distally; uncus strongly curved, with scales; appendix hooked, with a scaly edge and a small apophysis medially; embolus weakly sclerotized, with transparent distal projections. Retrolateral trichobothrium of tibia I at 4.4%; legs with short vertical setae on metatarsi and tarsi, without spines and curved setae; tarsus I with approximately 15 distinct pseudosegments.

Female: Similar to male, habitus as in Fig. S10C. Total length 3.37 (3.84 with clypeus), carapace 1.01 long, 1.17 wide, opisthosoma 2.63 long, 0.94 wide; tibia I: 7.44; tibia I L/d: 63. Distance PME-PME 0.23; diameter PME 0.11; distance PME-ALE 0.06; AME absent. Sternum wider than long (0.80/0.71). Opisthosoma yellowish. Ocular area brown, without eye-stalks. External genitalia (Fig. S11A) with a weakly sclerotized knob. Vulva (Fig. S11B) with an anterior arch and two pore plates.

Variation: Tibia I in males (n=6): 6.79–9.10 (mean: 7.85). Tibia I in females (n=27): 5.19–7.44 (mean: 6.95).

Distribution. Indonesia (West Sumatra, Jambi).

Natural history. The species was found in the entrance zones of caves and in bamboo internodes.

Text S3. Description of *Uthina javaensis* Yao & Li sp. nov.

urn:lsid:zoobank.org:act:560EF029-35C9-49CC-81BF-5D42E45CA3C4

Fig. S12

Material examined. Holotype: female, Gua (cave) Gajah (7°56.662′S, 110°26.404′E, elevation 234 m), Mangunan Village, Bantul, Java, Indonesia, 27 August 2014, H. Zhao and Z. Yao leg.

Other material. One female, same data as holotype.

Etymology. The specific name refers to the type locality; adjective.

Diagnosis. This species can be distinguished from all other *Uthina* species by strongly protruding medially vulval anterior arch (Fig. S12B) and by unique shape of vulval pore plates (Fig. S12B).

Description. Female (holotype): Total length 4.74 (5.13 with clypeus), carapace 1.10 long, 1.23 wide, opisthosoma 3.64 long, 1.25 wide. Leg I: 34.74 (8.33 + 0.52 + 8.72 +15.13 + 2.04), leg II: 20.75 (5.64 + 0.51 + 5.45 + 8.33 + 1.10), leg III, leg IV missing; tibia I L/d: 73. Distance PME-PME 0.22; diameter PME 0.12; distance PME-ALE 0.05; AME absent. Sternum wider than long (0.84/0.78). Habitus as in Figs S12C–E. Carapace yellowish, with brown marks extending to ocular area; ocular area brown; sternum brown. Legs yellowish, distal parts of femora brown, distal parts of tibiae whitish, subdistal parts of tibiae brown, darker rings absent. Opisthosoma yellowish, with brown spots dorsally and laterally. Ocular area elevated; without eye-stalks. Thoracic furrow absent. External genitalia (Fig. S12A) with a weakly sclerotized knob. Vulva (Fig. S12B) with an anterior arch and two pore plates. Retrolateral trichobothrium of tibia I at 4.6%; legs with short vertical setae on metatarsi and tarsi, without spines and curved setae; tarsus I with approximately 13 distinct pseudosegments.

Male: Unknown.

Variation: Unknown. Leg I missing in another female.

Distribution. Indonesia (Java).

Natural history. The species was found in the entrance zone of the cave.

Text S4. Description of *Uthina muangensis* Yao & Li sp. nov.

urn:lsid:zoobank.org:act:5736AB8A-46E5-4E50-900C-0E7CE3041D87

Figs S13–S15

Material examined. Holotype: male, Sawamg Cave (13°28.014′N, 99°44.014′E, elevation 44 m), Thuang Luang, Muang District, Ratchaburi, Thailand, 31 October 2014, H. Zhao, Y. Li and Z. Chen leg. Paratypes: two females, same data as holotype.

Other material. Three males, six females and one juvenile, same data as holotype.

Etymology. The specific name refers to the type locality; adjective.

Diagnosis. This species resembles *U. saiyokensis* sp. nov., in having similar male chelicerae (Fig. S14D) and procursus (Figs S13C–D), but can be distinguished by proximal apophysis of uncus indistinct (Fig. S14A), by wide and medially strongly protruding appendix (Fig. S14A), by knob of female external genitalia not swollen distally (Fig. S15A), and by nearly elliptic vulval pore plates (Fig. S15B).

Description. Male (holotype): Total length 4.82 (5.06 with clypeus), carapace 1.30 long, 1.50 wide, opisthosoma 3.52 long, 1.30 wide. Leg I missing, leg II: 35.85 (9.17 + 0.67 + 9.23 + 15.38 + 1.40), leg III: 22.89 (6.67 + 0.60 + 5.96 + 8.72 + 0.94), leg IV: 29.73 (8.40 + 0.60 + 7.56 + 11.92 + 1.25). Distance PME-PME 0.43; diameter PME 0.11; distance PME-ALE 0.03; AME absent. Sternum wider than long (0.95/0.80). Habitus as in Fig. S14B. Carapace yellowish, with large brown radiating marks extending to ocular area; ocular area yellowish, with brown marks; sternum dark brown. Legs yellowish, distal parts of femora and tibiae whitish, darker rings absent. Opisthosoma yellowish, with spots posteriorly. Ocular area elevated; each eye triad on top of a laterally directed eye-stalk. Thoracic furrow absent. Chelicerae as in Fig. S14D, with a pair of proximo-lateral apophyses and a pair of distal apophyses. Pedipalps as in Figs S13A–B; trochanter with a short retrolatero-ventral apophysis; femur with a dorsal apophysis proximally; procursus simple proximally but complex distally; uncus strongly curved, with scales and a thin distal transparent process; appendix wide, strongly protruding medially, with scales; embolus weakly sclerotized, with some transparent projections distally. Legs with short vertical setae on metatarsi and tarsi, without spines and curved setae.

Female: Similar to male, habitus as in Fig. S14C. Total length 5.08 (5.31 with clypeus), carapace 1.40 long, 1.52 wide, opisthosoma 3.68 long, 1.40 wide; tibia I: 13.14; tibia I L/d: 82. Distance PME-PME 0.29; diameter PME 0.12; distance PME-ALE 0.03; AME absent. Sternum wider than long (0.98/0.83). Opisthosoma without spots. Ocular area without eye-stalks. External genitalia (Fig. S15A) with a weakly sclerotized knob. Vulva (Fig. S15B) with an anterior arch and two nearly elliptic pore plates.

Variation: Unknown. Leg I missing in other specimens.

Distribution. Thailand (Ratchaburi).

Natural history. The species was found in the entrance zone of the cave.

Text S5. Description of *Uthina potharamensis* Yao & Li sp. nov.
urn:lsid:zoobank.org:act:4A034FDA-E209-4534-9881-99C4A22EB961
Figs S16–S18

Material examined. Holotype: male, Tham (cave) Nam (13°41.860′N, 99°45.415′E), Nang Kaeo, Potharam District, Ratchaburi, Thailand, 21 October 2014, H. Zhao, Y. Li and Z. Chen leg. Paratypes: two females, same data as holotype.

Other material. One male, four females and five juveniles, same data as holotype.

Etymology. The specific name refers to the type locality; adjective.

Diagnosis. This species resembles *U. ratchaburi* Huber, 2011, in having similar male chelicerae (Fig. S17D), uncus (Fig. S17A), and appendix (Fig. S17A), but can be distinguished by presence of a membranous lamella proximally on large, weakly sclerotized prolateral apophysis of procursus (arrow in Fig. S16D), by knob of female external genitalia not bifurcated (Fig. S18A), and by long distance between two vulval pore plates (Fig. S18B).

Description. Male (holotype): Total length 3.72 (3.88 with clypeus), prosoma 1.06 long, 1.22 wide, opisthosoma 2.66 long, 0.80 wide. Leg I: 43.79 (10.58 + 0.71 + 10.64 + 19.42 + 2.44), leg II: 25.16 (6.41 + 0.53 + 6.54 + 10.51 + 1.17), leg III: 16.76 (4.74 + 0.50 + 4.17 + 6.41 + 0.94), leg IV: 22.16 (6.41 + 0.50 + 5.51 + 8.65 + 1.09). Distance PME-PME 0.22; diameter PME 0.10; distance PME-ALE 0.03; AME absent. Sternum wider than long (0.75/0.70). Habitus as in Fig. S17B. Carapace yellowish, with light brown marks extending to ocular area; ocular area yellowish, with light brown marks; sternum brown. Legs yellowish, darker rings absent. Opisthosoma yellowish, with spots dorsally. Ocular area elevated; each eye triad on top of a short, laterally directed eye-stalk. Thoracic furrow absent. Chelicerae as in Fig. S17D, with a pair of proximo-lateral apophyses and a pair of distal apophyses. Pedipalps as in Figs S16A–B; trochanter with a short retrolatero-ventral apophysis; femur with a dorsal apophysis proximally; procursus simple proximally but complex distally; uncus strongly curved, with scales and a thin distal transparent process; appendix hooked and with scales; embolus weakly sclerotized. Legs with short vertical setae on tibiae, metatarsi and tarsi; without spines and curved setae.

Female: Similar to male, habitus as in Fig. S17C. Total length 4.37 (4.68 with clypeus), carapace 1.17 long, 1.22 wide, opisthosoma 3.20 long, 1.02 wide; tibia I: 11.09; tibia I L/d: 84. Distance PME-PME 0.24; diameter PME 0.10; distance PME-ALE 0.03. Sternum wider than long (0.95/0.75). Carapace and ocular area with dark brown marks; ocular area without eye-stalks. External genitalia (Fig. S18A) with a weakly sclerotized knob. Vulva (Fig. S18B) with an anterior arch and two elliptic pore plates.

Variation: Leg I missing in another male. Tibia I in females (n=4): 9.29–11.12 (mean: 10.21).

Distribution. Thailand (Ratchaburi).

Natural history. The species was found in the entrance zone of the cave.

Text S6. Description of *Uthina saiyokensis* Yao & Li sp. nov.
urn:lsid:zoobank.org:act:0780BF58-5336-4F81-8D2E-9DDDEE3CA7A6
Figs S19–S21

Material examined. Holotype: male, Cave without a name (14°11.400′N, 99°01.555′E, elevation 334 m), Wang Krachae, Sai Yok District, Kanchanaburi, Thailand, 2 November 2014, H. Zhao, Y. Li and Z. Chen leg. Paratype: one female, same data as holotype.

Other material. Two males, two females and one juvenile, same data as holotype. One male and nine females, Cave without a name (14°11.429′N, 99°01.594′E, elevation 324 m), Wang Krachae, Sai Yok District, Kanchanaburi, Thailand, 2 November 2014, H. Zhao, Y. Li and Z. Chen leg. Two females and three juveniles, Cave without a name (14°12.219′N, 99°01.666′E, elevation 325 m), Wang Krachae, Sai Yok District, Kanchanaburi, Thailand, 1 November 2014, H. Zhao, Y. Li and Z. Chen leg. One female and four juveniles, Cave without a name (14°12.127′N, 99°01.195′E, elevation 438 m), Wang Krachae, Sai Yok District, Kanchanaburi, Thailand, 1 November 2014, H. Zhao, Y. Li and Z. Chen leg. Three juveniles, Cave without a name (14°12.182′N, 99°01.416′E, elevation 342 m), Wang Krachae, Sai Yok District, Kanchanaburi, Thailand, 1 November 2014, H. Zhao, Y. Li and Z. Chen leg.

Etymology. The specific name refers to the type locality; adjective.

Diagnosis. This species resembles *U. muangensis* sp. nov., in having similar male chelicerae (Fig. S20D) and procursus (Figs S19C–D), but can be distinguished by presence of a distinct apophysis proximally on uncus (arrow in Fig. S20A), by slender appendix and appendix not protruding medially (Fig. S20A), by knob of female external genitalia swollen distally (Fig. S21A), and by nearly triangular vulval pore plates (Fig. S21B). This species also resembles *U. sarikaensis* sp. nov., in having similar knob of female external genitalia (Fig. S21A), but can be distinguished by nearly triangular vulval pore plates (Fig. S21B).

Description. Male (holotype): Total length Total length 4.57 (4.94 with clypeus), carapace 1.21 long, 1.41 wide, opisthosoma 3.36 long, 1.22 wide. Leg I: 51.1 (11.86 + 0.76 + 12.63 + 23.33 + 2.52), leg II, leg III and leg IV missing; tibia I L/d: 94. Distance PME-PME 0.37; diameter PME 0.11; distance PME-ALE 0.03; AME absent. Sternum wider than long (0.90/0.76). Habitus as in Fig. S20B. Carapace yellowish, with large brown marks extending to ocular area; ocular area yellowish, with a brown band medially; sternum dark brown. Legs yellowish, distal parts of femora and tibiae whitish, darker rings absent. Opisthosoma yellowish, with spots dorsally. Ocular area elevated; each eye triad on top of a short, laterally directed eye-stalk. Thoracic furrow absent. Chelicerae as in Fig. S20D, with a pair of proximo-lateral apophyses and a pair of distal apophyses. Pedipalps as in Figs S19A–B; trochanter with a short retrolatero-ventral apophysis; femur with a dorsal apophysis proximally; procursus simple proximally but complex distally; uncus strongly curved, with scales; appendix hooked, with scales; embolus weakly sclerotized, with some transparent projections distally. Retrolateral trichobothrium of tibia I at 7.3%; legs with short vertical setae on metatarsi and tarsi, without spines and curved setae; tarsus I with about 5 distinct pseudosegments.

Female: Similar to male, habitus as in Fig. S20C. Total length 4.53 (4.81 with clypeus), carapace 1.13 long, 1.19 wide, opisthosoma 3.40 long, 1.41 wide. Distance PME-PME 0.25; diameter PME 0.10; distance PME-ALE 0.02; AME absent. Sternum wider than long (0.86/0.74). Opisthosoma without spots. Ocular area brown, with a dark brown band medially, without eye-stalks. External genitalia (Fig. S21A) with a weakly sclerotized knob. Vulva (Fig. S21B) with an anterior arch and two nearly triangular pore plates.

Variation: Tibia I in males (n=2): 10.58, 12.63. Tibia I in females (n=18): 7.05–11.54 (mean: 9.65).

Distribution. Thailand (Kanchanaburi).

Natural history. The species was found in the entrance zones of caves.

Text S7. Description of *Uthina sarikaensis* Yao & Li sp. nov.

urn:lsid:zoobank.org:act:B74F0176-11F0-4F91-AE1F-FD5F52A834DC

Fig. S22

Material examined. Holotype: female, Tham (Cave) Sarika (13°29.773′N, 99°44.229′E, elevation 48 m), Muang District, Ratchaburi, Thailand, 21 October 2014, H. Zhao, Y. Li and Z. Chen leg.

Etymology. The specific name refers to the type locality; adjective.

Diagnosis. This species resembles *U. potharamensis* sp. nov. and *U. saiyokensis* sp. nov., in having similar knob of female external genitalia (Fig. S22A), but can be distinguished from *U. potharamensis* sp. nov. by short distance between two vulval pore plates (Fig. S22B), distinguished from *U. saiyokensis* sp. nov. by nearly oval vulval pore plates (Fig. S22B).

Description. Female (holotype): Total length 4.03 (4.23 with clypeus), carapace 1.09 long, 1.17 wide, opisthosoma 2.94 long, 0.92 wide. Leg I, leg III and leg IV missing, leg II: 21.78 (4.81 + 0.48 + 4.17 + 6.15 + 0.94). Distance PME-PME 0.29; diameter PME 0.06; distance PME-ALE 0.03; AME absent. Sternum wider than long (0.79/0.71). Habitus as in Figs S22C–E. Carapace yellowish, with brown marks extending to ocular area; ocular area yellowish, with a brown band medially and brown marks laterally; sternum brown. Legs yellowish, distal parts of femora whitish, darker rings absent. Opisthosoma yellowish, with spots posteriorly. Ocular area elevated; without eye-stalks. Thoracic furrow absent. External genitalia (Fig. S22A) with a weakly sclerotized knob. Vulva (Fig. S22B) with an anterior arch and two nearly oval pore plates. Legs with short vertical setae on metatarsi and tarsi, without spines and curved setae.

Male: Unknown.

Variation: Unknown.

Distribution. Thailand (Ratchaburi).

Natural history. The species was found in the entrance zone of the cave.

Text S8. Description of *Uthina sulawesiensis* Yao & Li sp. nov.

urn:lsid:zoobank.org:act:4458BB80-B637-4E9D-924E-B02BD4AD5BB3

Figs S23–S25

Material examined. Holotype: male, Cave without a name (4°52.344′S, 119°53.858′E, elevation 417 m), Maros, South Sulawesi, Indonesia, 23 August 2014, H. Zhao and Z. Yao leg. Paratype: one female, same data as holotype.

Other material. One female, Gua (cave) Liang Gose (4°56.304′S, 119°50.341′E, elevation 355 m), Benteng Orchard, Cenrana Village, Maros, South Sulawesi, Indonesia, 23

August 2014, H. Zhao and Z. Yao leg.

Etymology. The specific name refers to the type locality; adjective.

Diagnosis. This species resembles one type of *U. luzonica* from North Sulawesi in Huber (2011), in having similar uncus (Fig. S24A) and female external genitalia (Fig. S25A), but can be distinguished by appendix not bifurcated distally (Fig. S24A) and by large, wide vulval pore plates (Fig. S25B). This species can be distinguished from other species in *Uthina* by presence of a large membranous process distally on uncus (Fig. S24A), by presence of a large angular apophysis close to large weakly sclerotized apophysis of procursus (arrow in Fig. S23D), and by unique shape of vulval pore plates (Fig. S25B).

Description. Male (holotype): Total length 4.16 (4.42 with clypeus), carapace 1.03 long, 1.09 wide, opisthosoma 3.13 long, 0.88 wide. Leg I missing, leg II: 21.78 (5.78 + 0.47 + 5.51 + 8.72 + 1.30), leg III: 14.26 (4.10 + 0.43 + 3.53 + 5.26 + 0.94), leg IV: 20.61 (6.09 + 0.44 + 5.13 + 8.01 + 0.94). Distance PME-PME 0.27; diameter PME 0.08; distance PME-ALE 0.08; AME absent. Sternum wider than long (0.77/0.70). Habitus as in Fig. S24B. Carapace yellowish, with brown marks extending to ocular area; ocular area brown; sternum brown. Legs yellowish, distal parts of femora and tibiae whitish, subdistal parts of femora and tibiae brown, darker rings absent. Opisthosoma yellowish, with spots dorsally. Ocular area elevated; each eye triad on top of a short, laterally directed eye-stalk. Thoracic furrow absent. Chelicerae as in Fig. S24D, with a pair of proximo-lateral apophyses and a pair of distal apophyses. Pedipalps as in Figs S23A–B; trochanter with a short retrolatero-ventral apophysis; femur with a dorsal apophysis proximally; procursus simple proximally but complex distally; uncus strongly curved, with scales, a larger transparent process distally, and a small, thin process distally; appendix hooked, with scales; embolus weakly sclerotized. Legs with short vertical setae on tibiae, metatarsi and tarsi; without spines and curved setae.

Female: Similar to male, habitus as in Fig. S24C. Total length 3.38 (3.56 with clypeus), carapace 1.00 long, 1.08 wide, opisthosoma 2.38 long, 0.96 wide; leg I missing. Distance PME-PME 0.20; diameter PME 0.11; distance PME-ALE 0.04. Sternum wider than long (0.69/0.67). Ocular area without eye-stalks. External genitalia (Fig. S25A) with a weakly sclerotized knob. Vulva (Fig. S25B) with an anterior arch and two pore plates.

Variation: Tibia I in another female: 8.46.

Distribution. Indonesia (South Sulawesi).

Natural history. The species was found in the entrance zones of caves.

Text S9. Description of *Uthina wongpromi* Yao & Li sp. nov.

urn:lsid:zoobank.org:act:C8BDC3F2-20D7-49E6-A700-4A1E5390015D

Figs S26–S27

Material examined. Holotype: male, Phra Kha Yang Cave (10°19.568′N, 98°45.908′E, elevation 6 m), Kraburi District, Ranong, Thailand, 28 October 2014, H. Zhao, Y. Li and Z. Chen leg.

Other material. One juvenile, Tham Than La Cave (16°54.816′N, 100°43.227′E, elevation 510 m), Ban Kaset Sut Village, Nakhothai District, Phitsanulok, Thailand, 17 October 2014, W. Prasit, Y. Li and Z. Chen leg. Two juveniles, Taraswan Cave (16°55.220′N,

100°43.19′E, elevation 460 m), Ban Tham Phrik Village, Nakhothai District, Phitsanulok, Thailand, 18 October 2014, H. Zhao, Y. Li and Z. Chen leg.

Etymology. The specific name is a patronym in honor of the collector Wongprom Prasit; noun (name) in genitive case.

Diagnosis. This species resembles true *U. luzonica* and one type of *U. luzonica* from Fiji in Huber (2011), in having similar uncus (Fig. S27A), but can be distinguished by distally bifurcated appendix (Fig. S27A); also distinguished from true *U. luzonica* by large bifurcated apophysis close to large weakly sclerotized apophysis of procursus (arrow in Fig. S26D; large angular apophysis not bifurcated in *U. luzonica*).

Description. Male (holotype): Total length 4.30 (4.49 with clypeus), carapace 1.02 long, 1.19 wide, opisthosoma 3.28 long, 1.06 wide. Leg I: 37.12 (8.85 + 0.56 + 9.29 + 16.22 + 2.20), leg II, leg III, leg IV missing; tibia I L/d: 68. Distance PME-PME 0.32; diameter PME 0.13; distance PME-ALE 0.05; AME absent. Sternum wider than long (0.85/0.80). Habitus as in Fig. S27B. Carapace yellowish, with brown marks extending to ocular area, ocular area dark brown; sternum dark brown. Legs yellowish, distal parts of femora and tibiae whitish, darker rings absent. Opisthosoma yellowish, with spots dorsally and laterally. Ocular area elevated; each eye triad on top of a short, laterally directed eye-stalk. Thoracic furrow absent. Chelicerae as in Fig. S27C, with a pair of proximo-lateral apophyses and a pair of distal apophyses. Pedipalps as in Figs S26A–B; trochanter with a short retrolatero-ventral apophysis; femur with a dorsal apophysis proximally; procursus simple proximally but complex distally; uncus strongly curved, with scales and a thin distal transparent process; appendix hooked, with scales, bifurcated distally; embolus weakly sclerotized. Retrolateral trichobothrium of tibia I at 3.4%; legs with short vertical setae on tibiae, metatarsi and tarsi; without spines and curved setae; tarsus I with approximately 12 distinct pseudosegments.

Female: Unknown.

Variation: Unknown.

Distribution. Thailand (Ranong, Phitsanulok).

Natural history. The species was found in the entrance zones of caves.

Text S10. Description of *Uthina yunchuni* Yao & Li sp. nov.

urn:lsid:zoobank.org:act:D94F2B1F-B4A1-4C12-868C-EBF3DE8BB683

Fig. S28

Material examined. Holotype: female, Cave without a name (8°05.122′N, 98°51.223′E, elevation 442 m), Ban Chong Plee, Muang District, Krabi, Thailand, 25 October 2014, H. Zhao, Y. Li and Z. Chen leg.

Other material. One female, same data as holotype.

Etymology. The specific name is a patronym in honor of the collector Yunchun Li; noun (name) in genitive case.

Diagnosis. This species resembles *P. zhigangi* sp. nov., in having similar vulval pore plates (Fig. S28B), but can be distinguished by wide knob of female external genitalia (Fig. S28A).

Description. Female (holotype): Total length 3.63 (3.88 with clypeus), carapace 1.00

long, 1.04 wide, opisthosoma 2.63 long, 080 wide. Leg I missing, leg II: 20.06 (5.45 + 0.44 + 5.13 + 7.82 + 1.22), leg III: 13.80 (3.97 + 0.44 + 3.53 + 5.00 + 0.86), leg IV: 19.46 (6.09 + 0.44 + 5.45 + 6.60 + 0.88). Distance PME-PME 0.25; diameter PME 0.08; distance PME-ALE 0.02; AME absent. Sternum wider than long (0.70/0.62). Habitus as in Figs S28C–E. Carapace yellowish, with brown marks extending to ocular area; ocular area yellowish, with brown marks; sternum brown. Legs yellowish, distal parts of femora and tibiae whitish, darker rings absent. Opisthosoma yellowish, with spots dorsally and laterally. Ocular area elevated; without eye-stalks. Thoracic furrow absent. External genitalia (Fig. S28A) with a weakly sclerotized knob. Vulva (Fig. S28B) with an anterior arch and two long pore plates. Legs with short vertical setae on metatarsi and tarsi, without spines and curved setae.

Male: Unknown.

Variation: Tibia I in another female: 8.97.

Distribution. Thailand (Krabi).

Natural history. The species was found in the entrance zone of the cave.

Text S11. Description of *Uthina zhigangi* Yao & Li sp. nov.

urn:lsid:zoobank.org:act:809004F3-A47B-45B5-BD93-59B0655D5E28

Fig. S29

Material examined. Holotype: female, Cave without a name (8°08.155′N, 98°48.430′E, elevation 89 m), Ban Khao Klom Nong Thale, Muang District, Krabi, Thailand, 26 October 2014, H. Zhao, Y. Li and Z. Chen leg.

Etymology. The specific name is a patronym in honor of the collector Zhigang Chen; noun (name) in genitive case.

Diagnosis. This species resembles *P. yunchuni* sp. nov., in having similar vulval pore plates (Fig. S29B), but can be distinguished by distally narrow knob of female external genitalia (Fig. S29A).

Description. Female (holotype): Total length 4.03 (4.36 with clypeus), carapace 1.03 long, 1.10 wide, opisthosoma 3.00 long, 0.92 wide. Leg I, leg II and leg IV missing, leg III: 14.26 (3.97 + 0.36 + 3.46 + 5.13 + 0.94). Distance PME-PME 0.13; diameter PME 0.09; distance PME-ALE 0.03; AME absent. Sternum wider than long (0.73/0.64). Habitus as in Figs S29C–E. Carapace yellowish, with brown marks extending to ocular area; ocular area yellowish with brown marks; sternum brown. Legs yellowish, distal parts of femora whitish, darker rings absent. Opisthosoma yellowish, with spots dorsally and laterally. Ocular area elevated; without eye-stalks. Thoracic furrow absent. External genitalia (Fig. S29A) with a weakly sclerotized knob. Vulva (Fig. S29B) with an anterior arch and two long pore plates. Legs with short vertical setae on metatarsi and tarsi, without spines and curved setae.

Male: Unknown.

Variation: Unknown.

Distribution. Thailand (Krabi).

Natural history. The species was found in the entrance zone of the cave.

Figure S6. Illustrations of *Uthina huahinensis* Yao & Li sp. nov., holotype male. A–B, pedipalp (A, prolateral view; B, retrolateral view); C–D, distal part of procursus (C, prolateral view; D, dorsal view, arrow points at small angular apophysis). Scale bars: 0.20 (A–B), 0.05 (C–D)

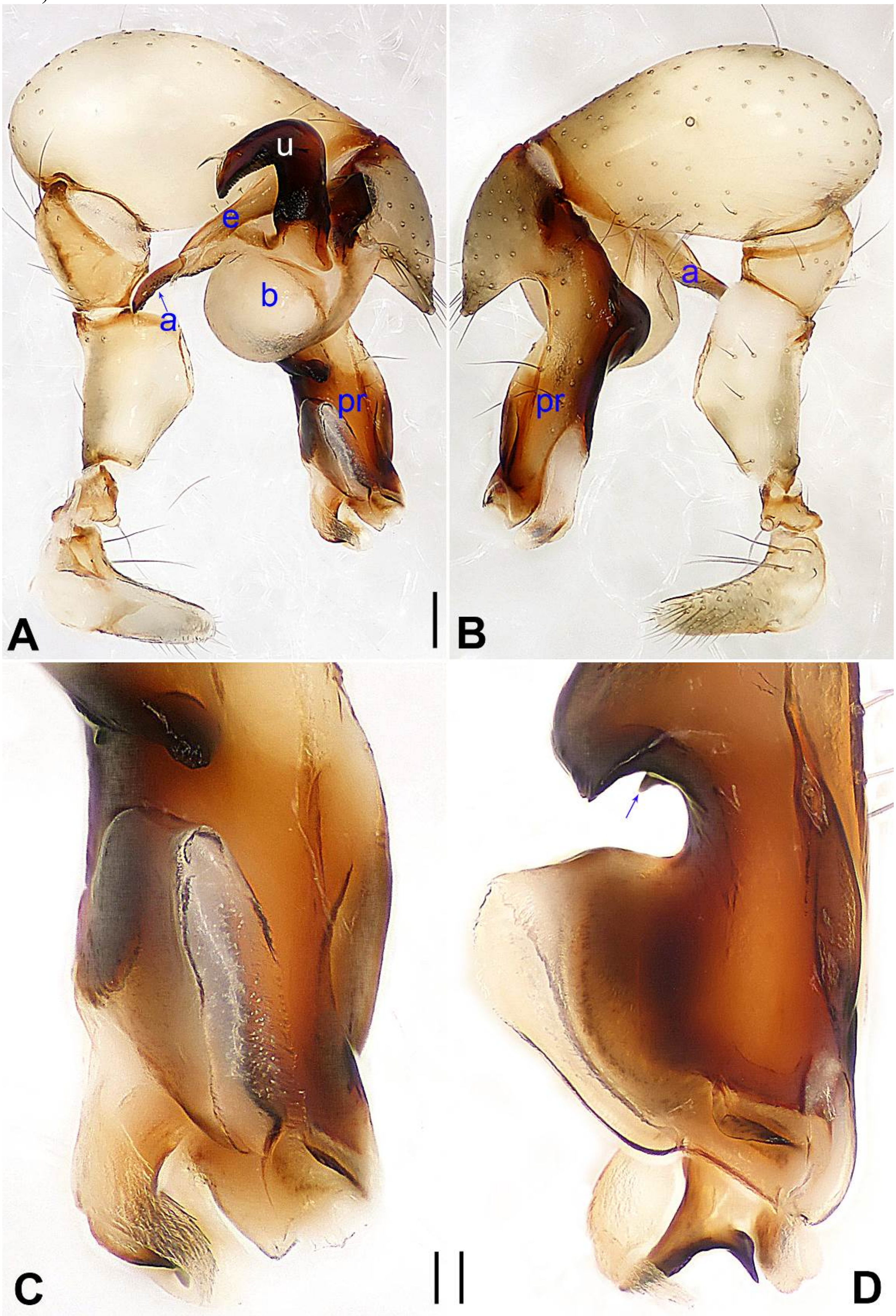

Figure S7. Illustrations of *Uthina huahinensis* Yao & Li sp. nov., holotype male (A–B, D) and paratype female (C). A, bulbal apophyses, prolateral view; B–C, habitus, dorsal view; D, chelicerae, frontal view. Scale bars: 0.05 (A), 0.50 (B–C), 0.10 (D)

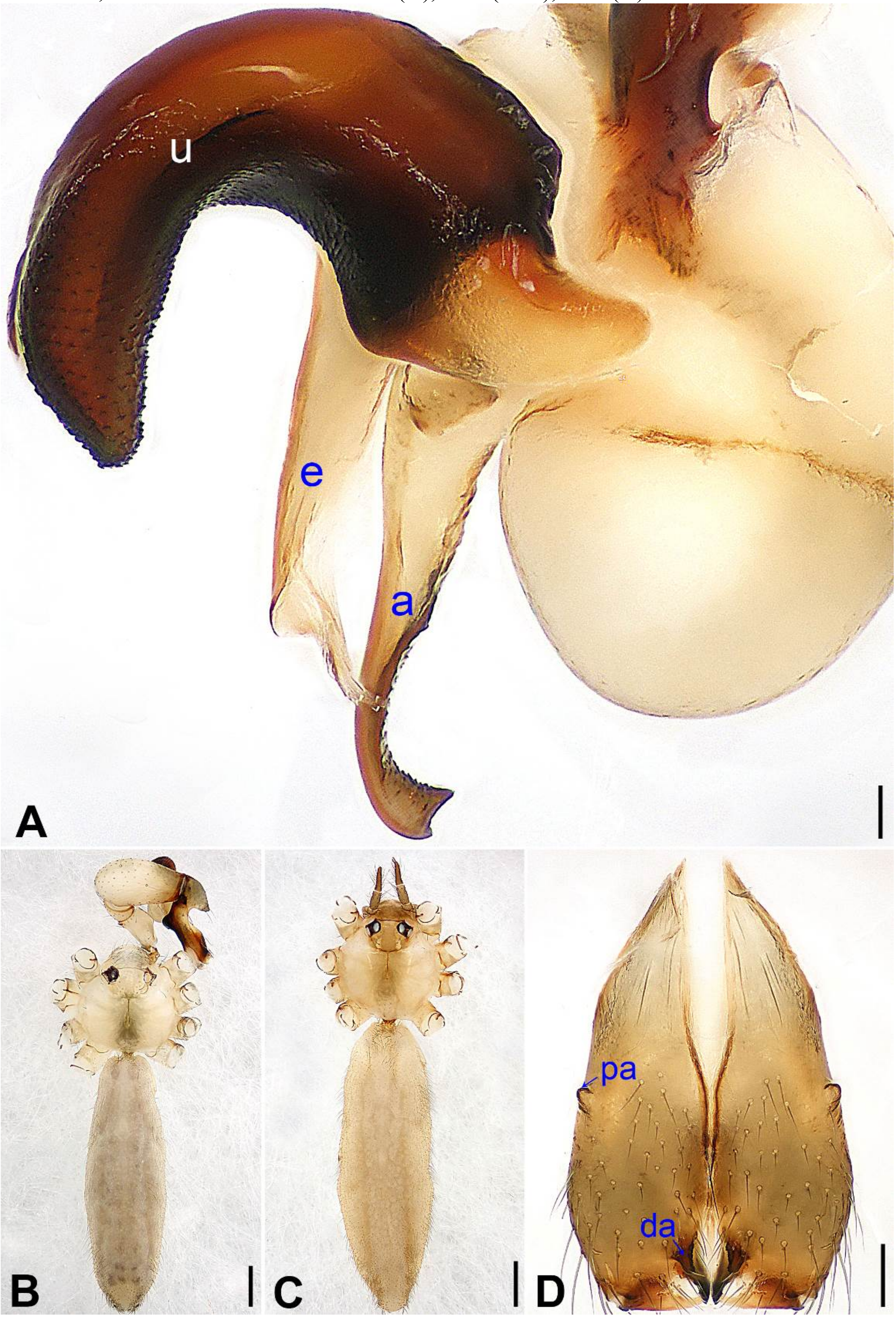

Figure S8. Illustrations of *Uthina huahinensis* Yao & Li sp. nov., paratype female. A, external genitalia, ventral view; B, vulva, dorsal view. Scale bars: 0.05

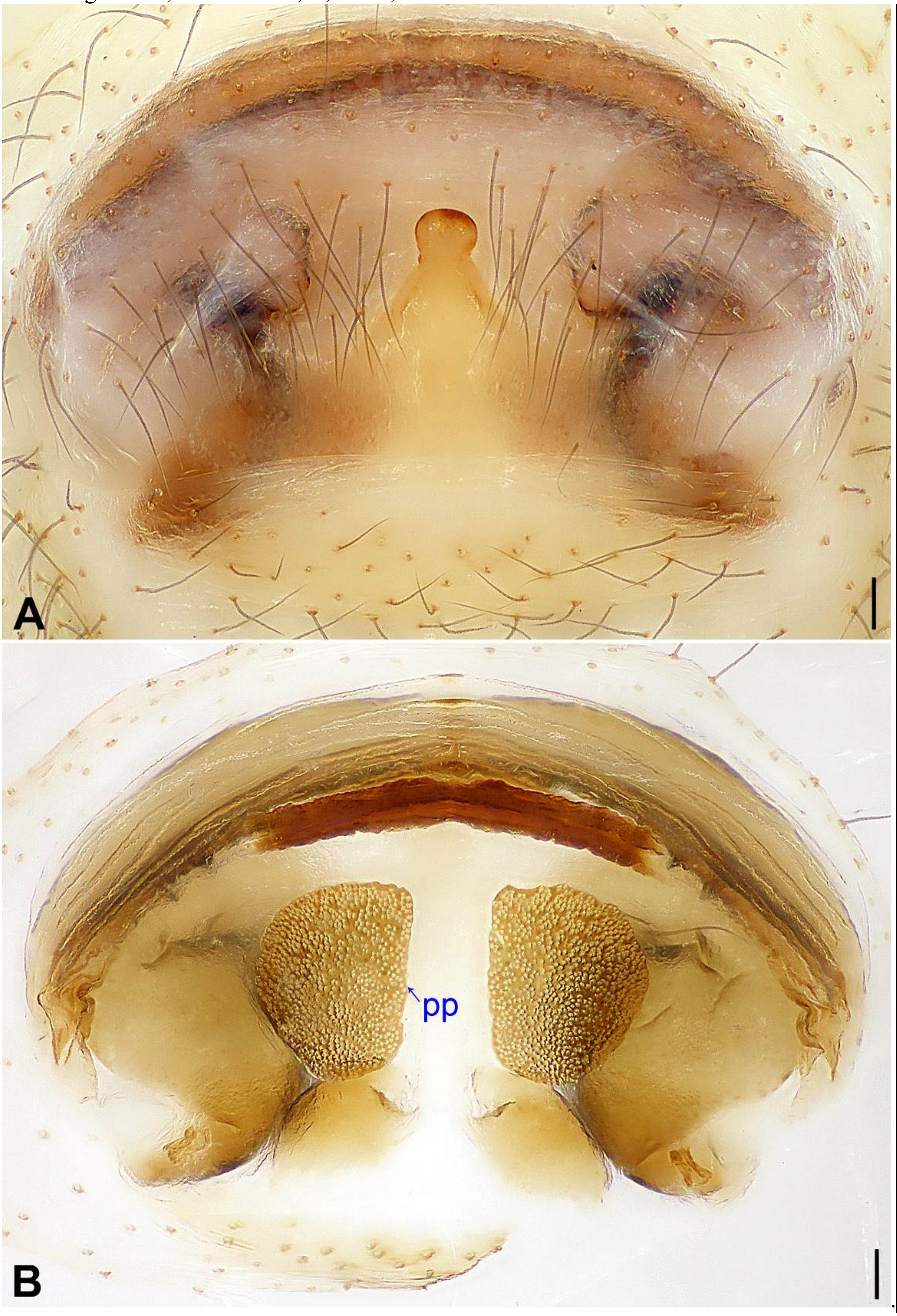

Figure S9. Illustrations of *Uthina huifengi* Yao & Li sp. nov., holotype male. A–B, pedipalp (A, prolateral view; B, retrolateral view); C–D, distal part of procursus (C, prolateral view; D, dorsal view, arrow points at small angular apophysis). Scale bars: 0.15 (A–B), 0.05 (C–D)

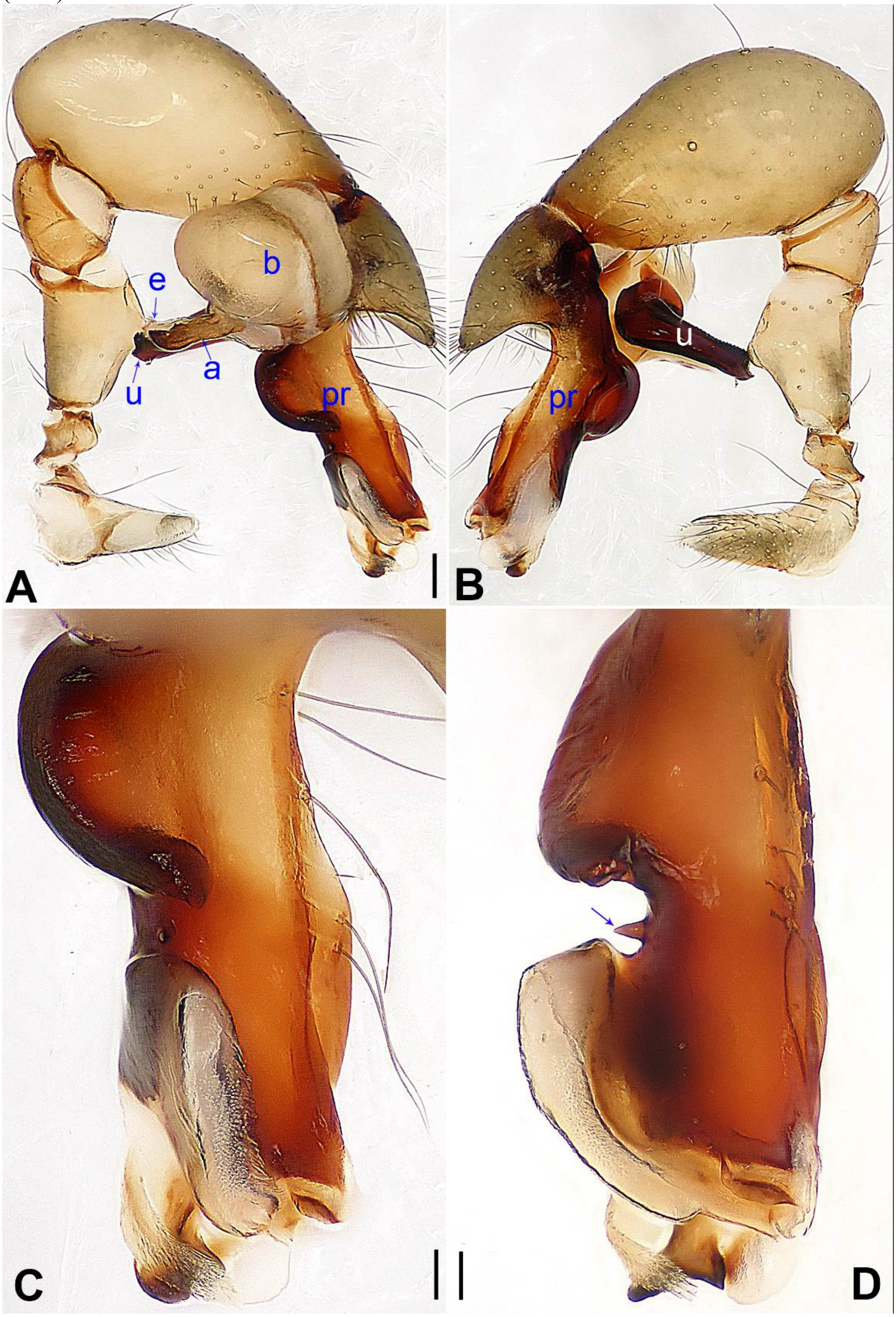

Figure S10. Illustrations of *Uthina huifengi* Yao & Li sp. nov., holotype male (A–B, D) and paratype female (C). A, bulbal apophyses, prolateral view; B–C, habitus, dorsal view; D, chelicerae, frontal view. Scale bars: 0.03 (A), 0.50 (B–C), 0.10 (D)

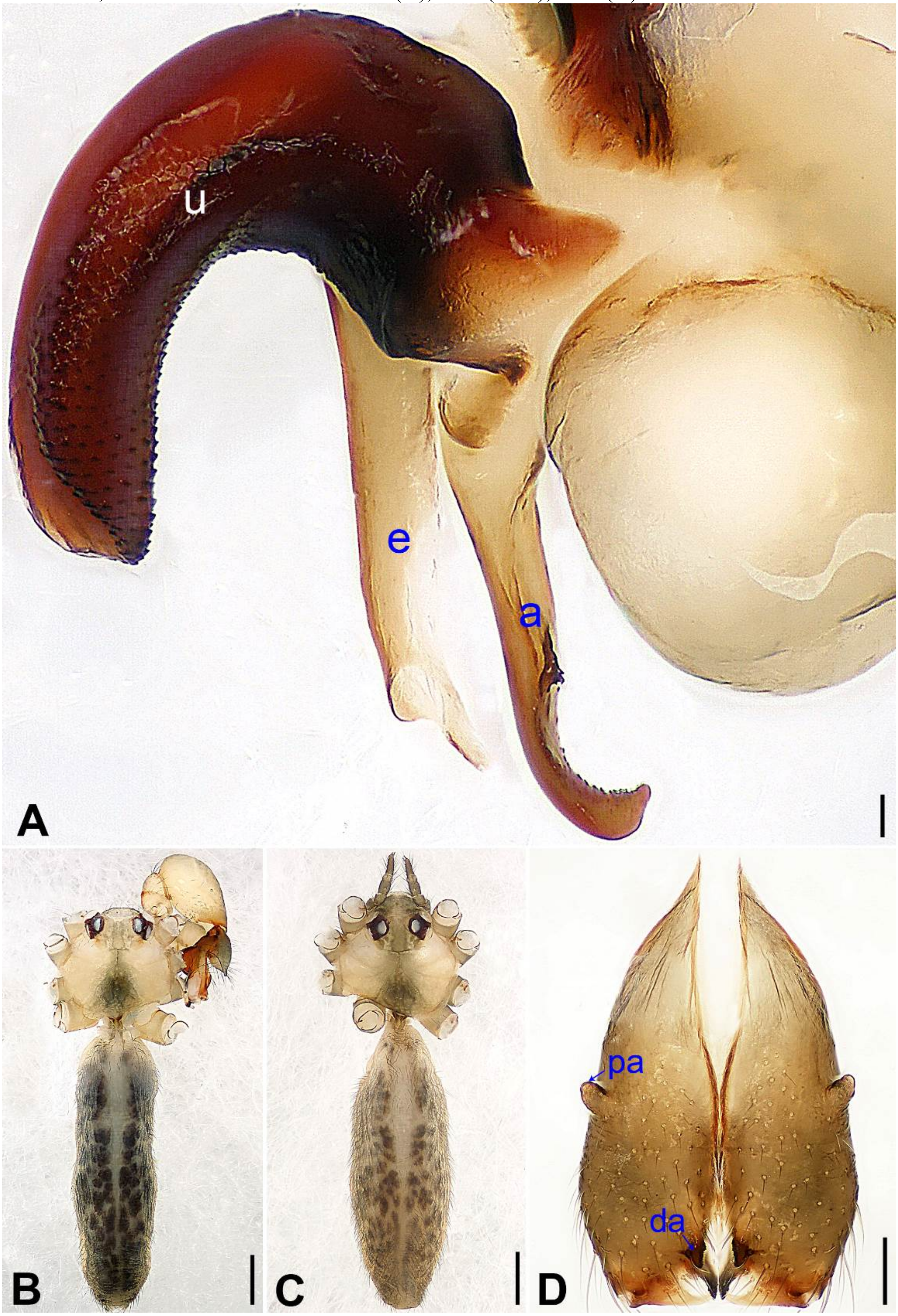

Figure S11. Illustrations of *Uthina huifengi* Yao & Li sp. nov., paratype female. A, external genitalia, ventral view; B, vulva, dorsal view. Scale bars: 0.05

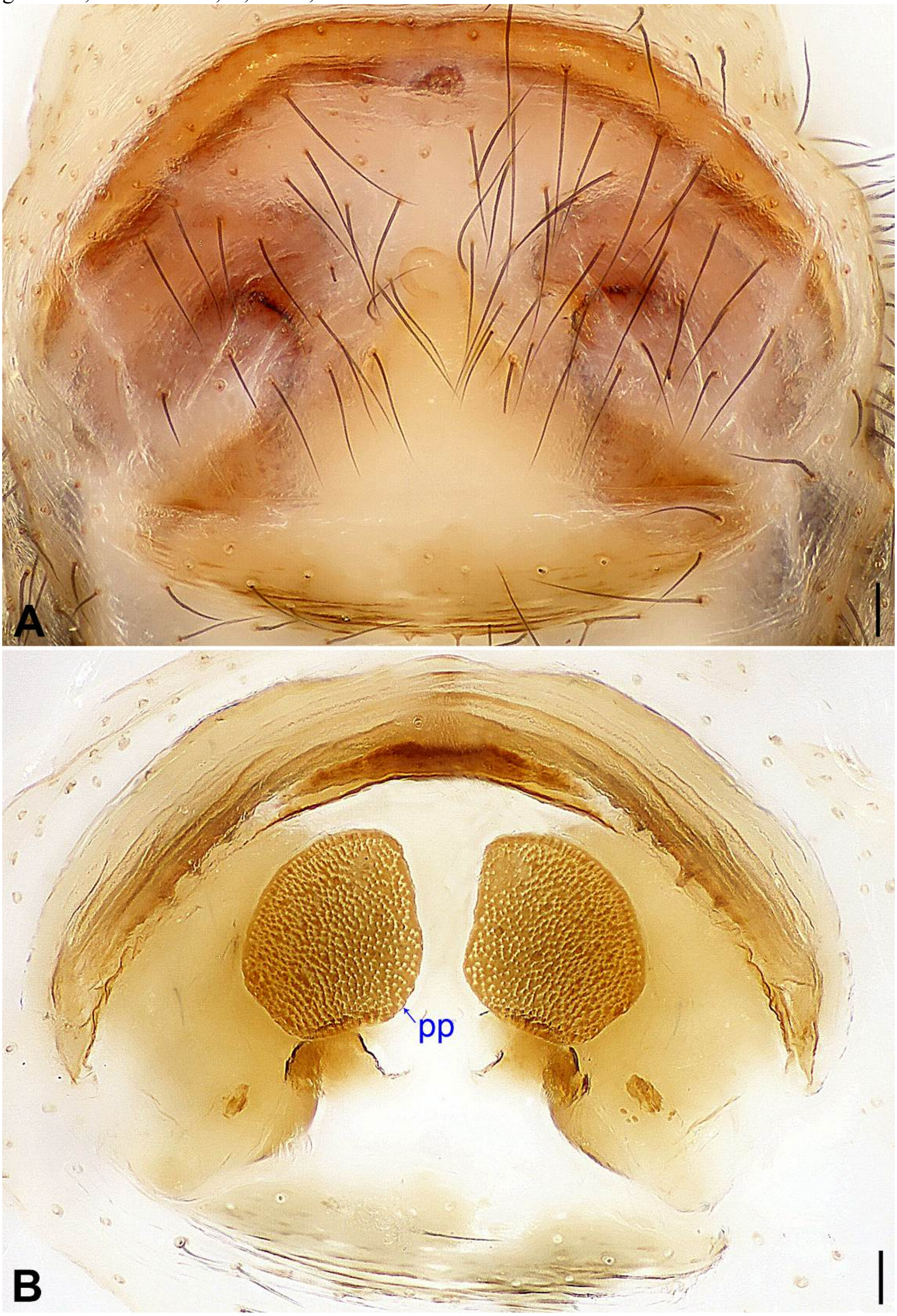

Figure S12. Illustrations of *Uthina javaensis* Yao & Li sp. nov., holotype female. A, external genitalia, ventral view; B, vulva, dorsal view; C–E, habitus (C, dorsal view; D, ventral view; E, lateral view). Scale bars: 0.10 (A–B), 1.00 (C–E)

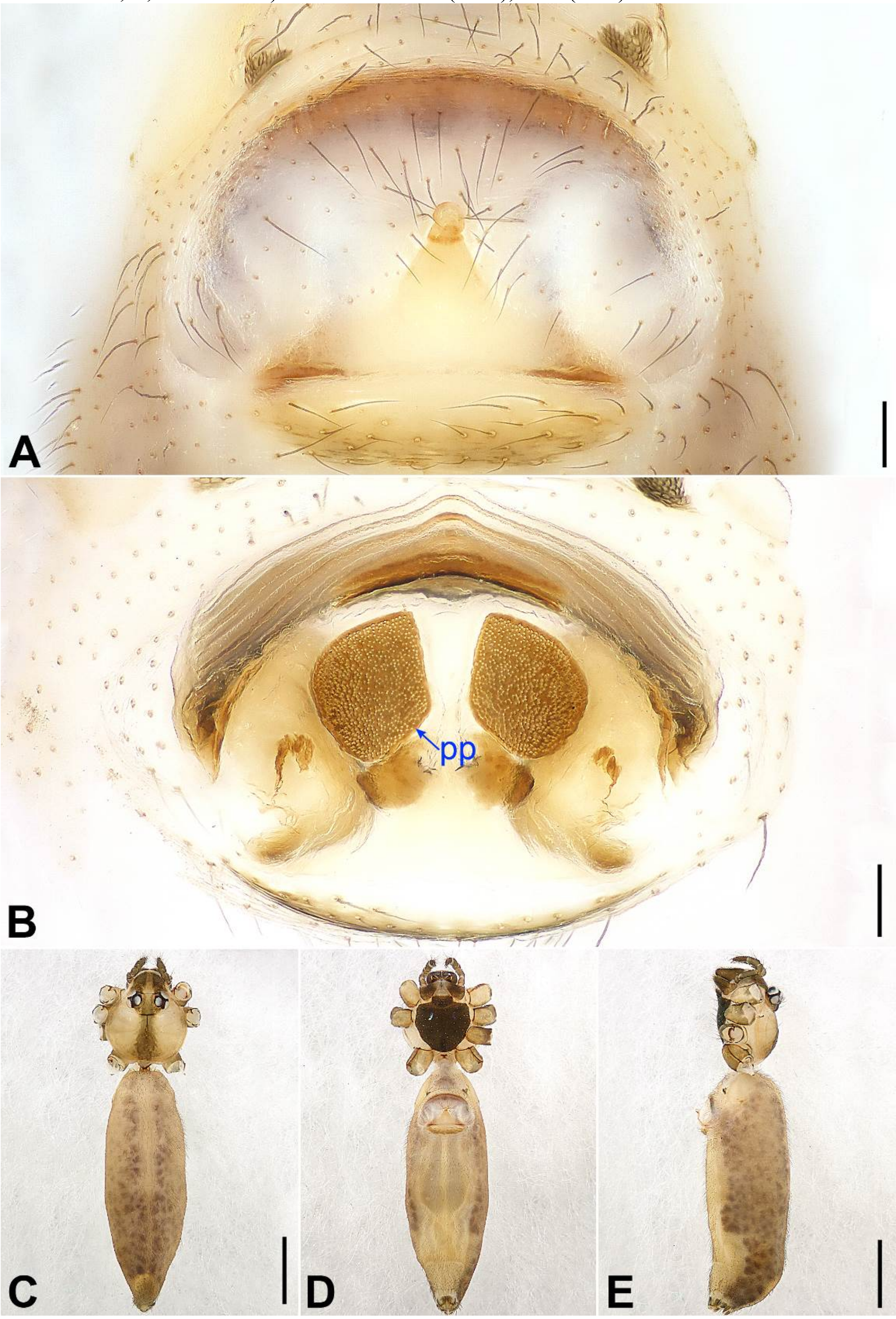

Figure S13. Illustrations of *Uthina muangensis* Yao & Li sp. nov., holotype male. A–B, pedipalp (A, prolateral view; B, retrolateral view); C–D, distal part of procursus (C, prolateral view; D, dorsal view). Scale bars: 0.20 (A–B), 0.05 (C–D)

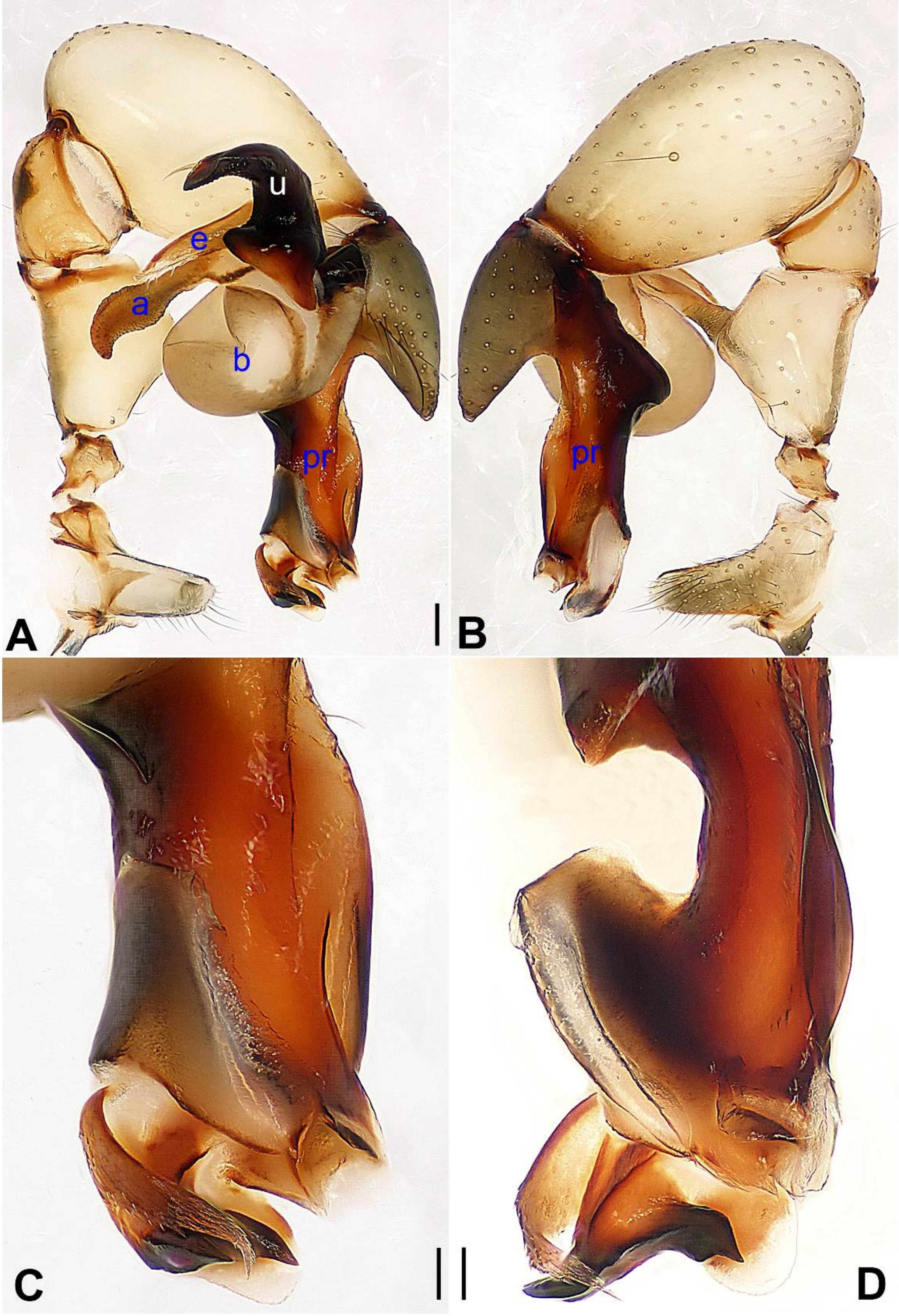

Figure S14. Illustrations of *Uthina muangensis* Yao & Li sp. nov., holotype male (A–B, D) and paratype female (C). A, bulbal apophyses, prolateral view; B–C, habitus, dorsal view; D, chelicerae, frontal view. Scale bars: 0.05 (A), 0.50 (B–C), 0.10 (D)

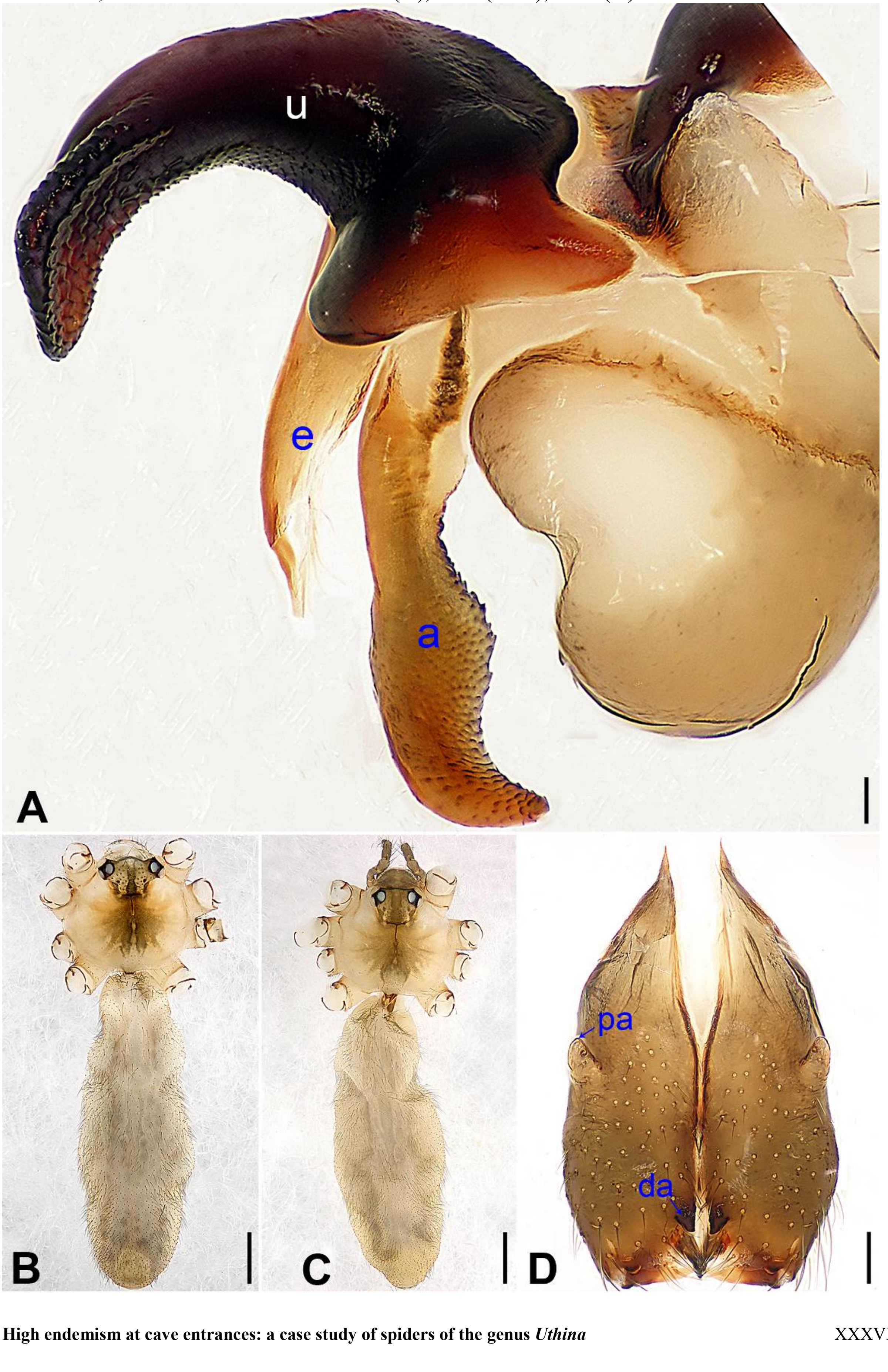

Figure S15. Illustrations of *Uthina muangensis* Yao & Li sp. nov., paratype female. A, external genitalia, ventral view; B, vulva, dorsal view. Scale bars: 0.05

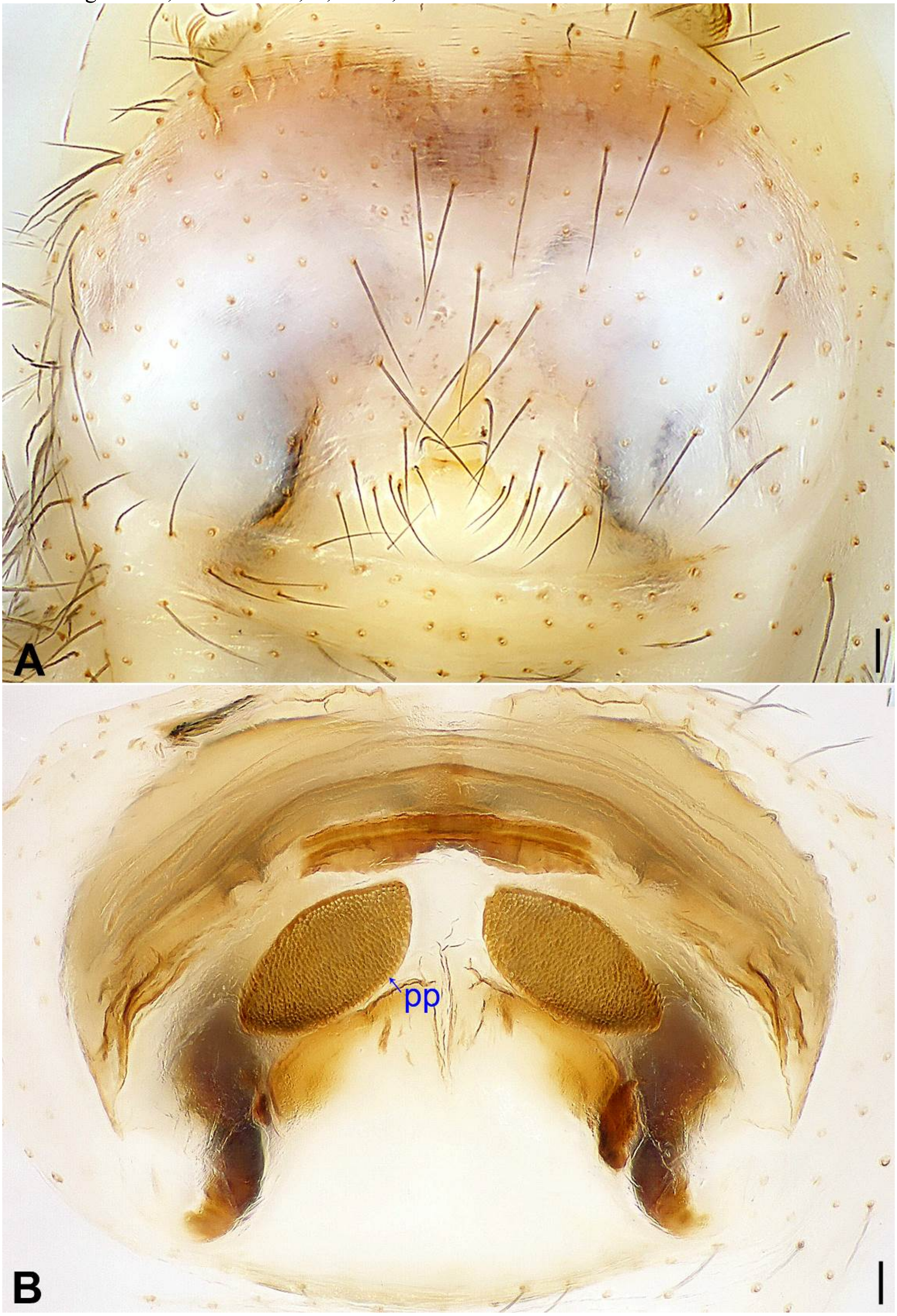

Figure S16. Illustrations of *Uthina potharamensis* Yao & Li sp. nov., holotype male. A–B, pedipalp (A, prolateral view; B, retrolateral view); C–D, distal part of procursus (C, prolateral view; D, dorsal view, arrow points at membranous lamella). Scale bars: 0.15 (A–B), 0.05 (C), 0.03 (D)

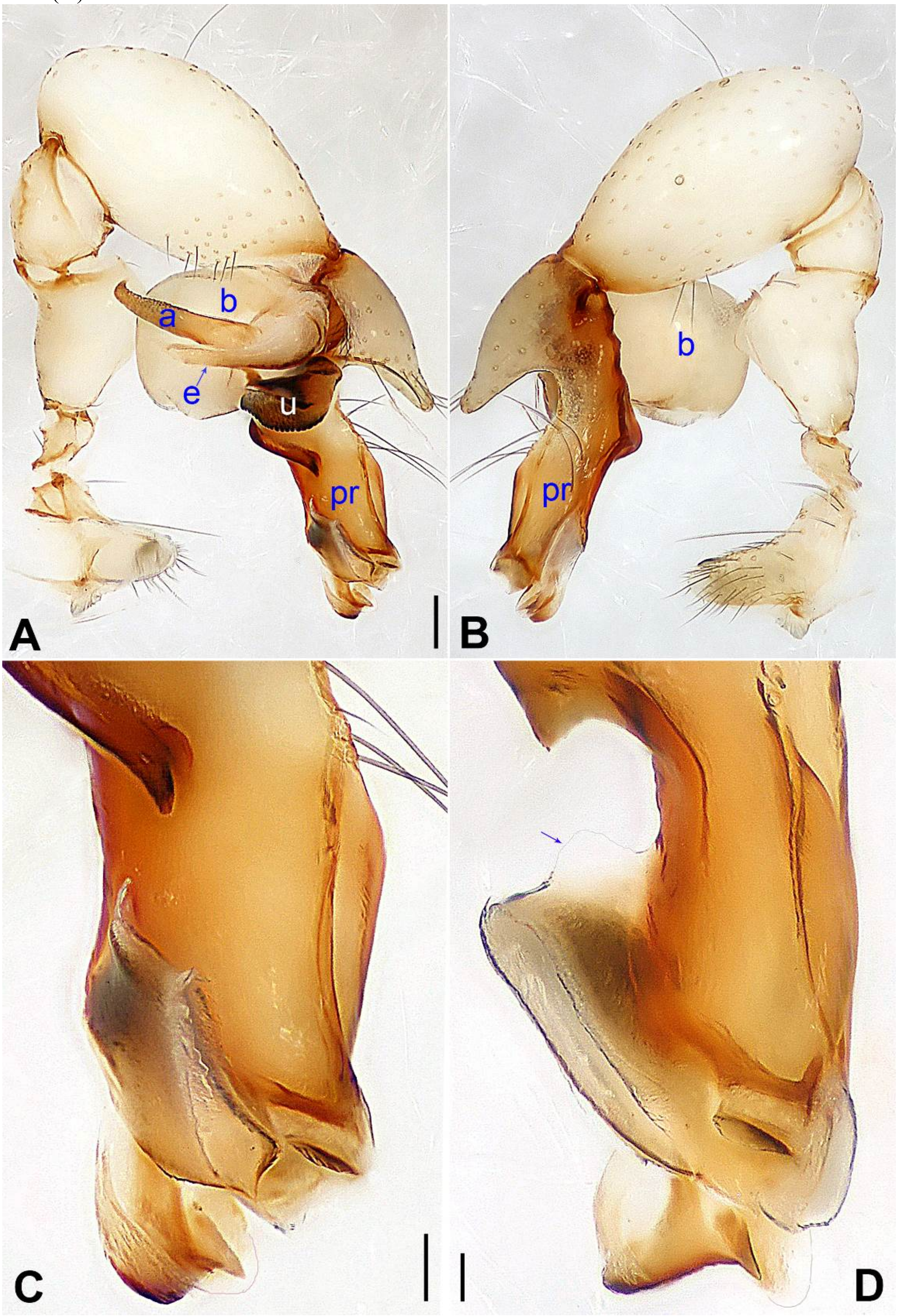

Figure S17. Illustrations of *Uthina potharamensis* Yao & Li sp. nov., holotype male (A–B, D) and paratype female (C). A, bulbal apophyses, prolateral view; B–C, habitus, dorsal view; D, chelicerae, frontal view. Scale bars: 0.03 (A), 0.50 (B–C), 0.10 (D)

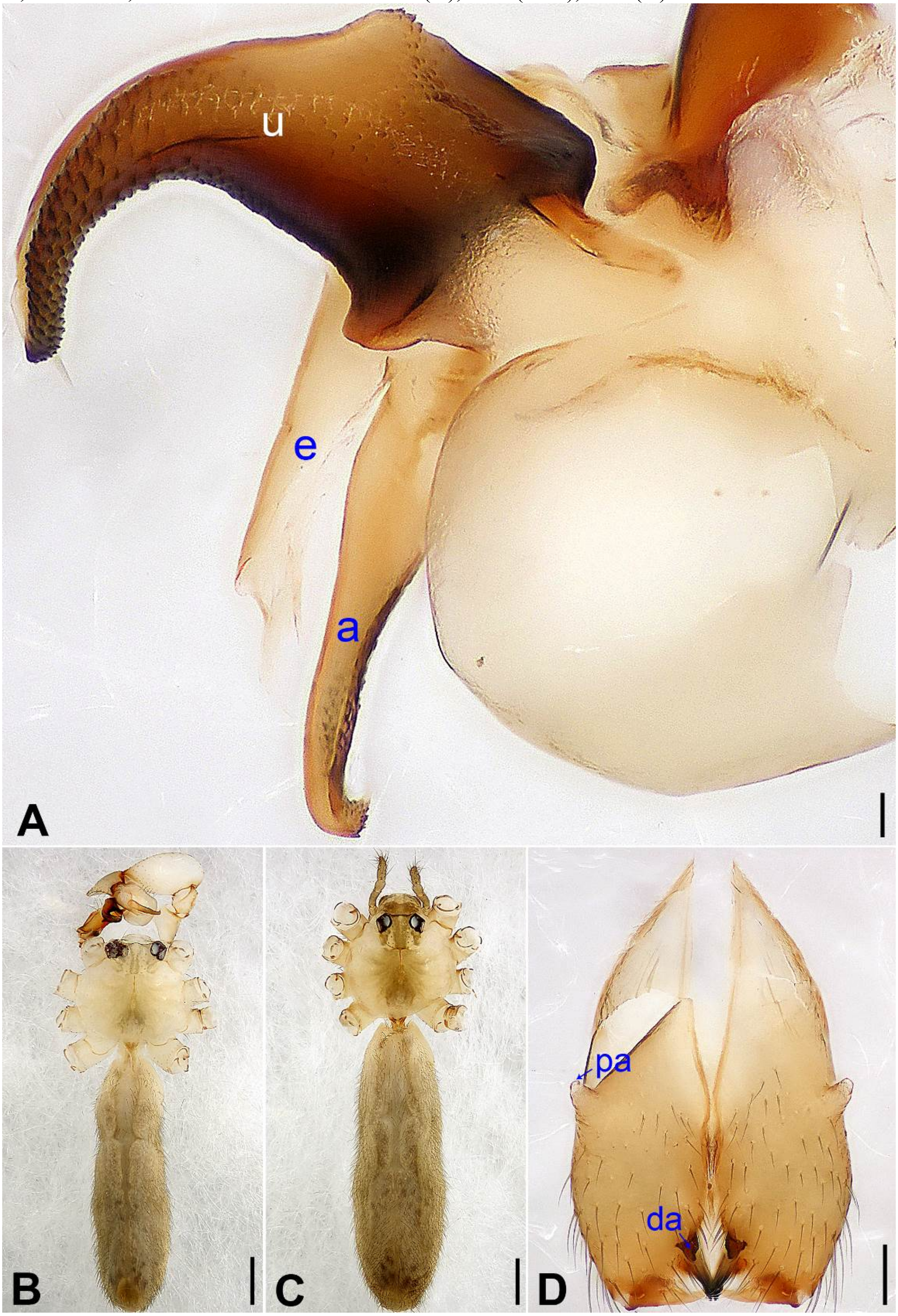

Figure S18. Illustrations of *Uthina potharamensis* Yao & Li sp. nov., paratype female. A, external genitalia, ventral view; B, vulva, dorsal view. Scale bars: 0.05

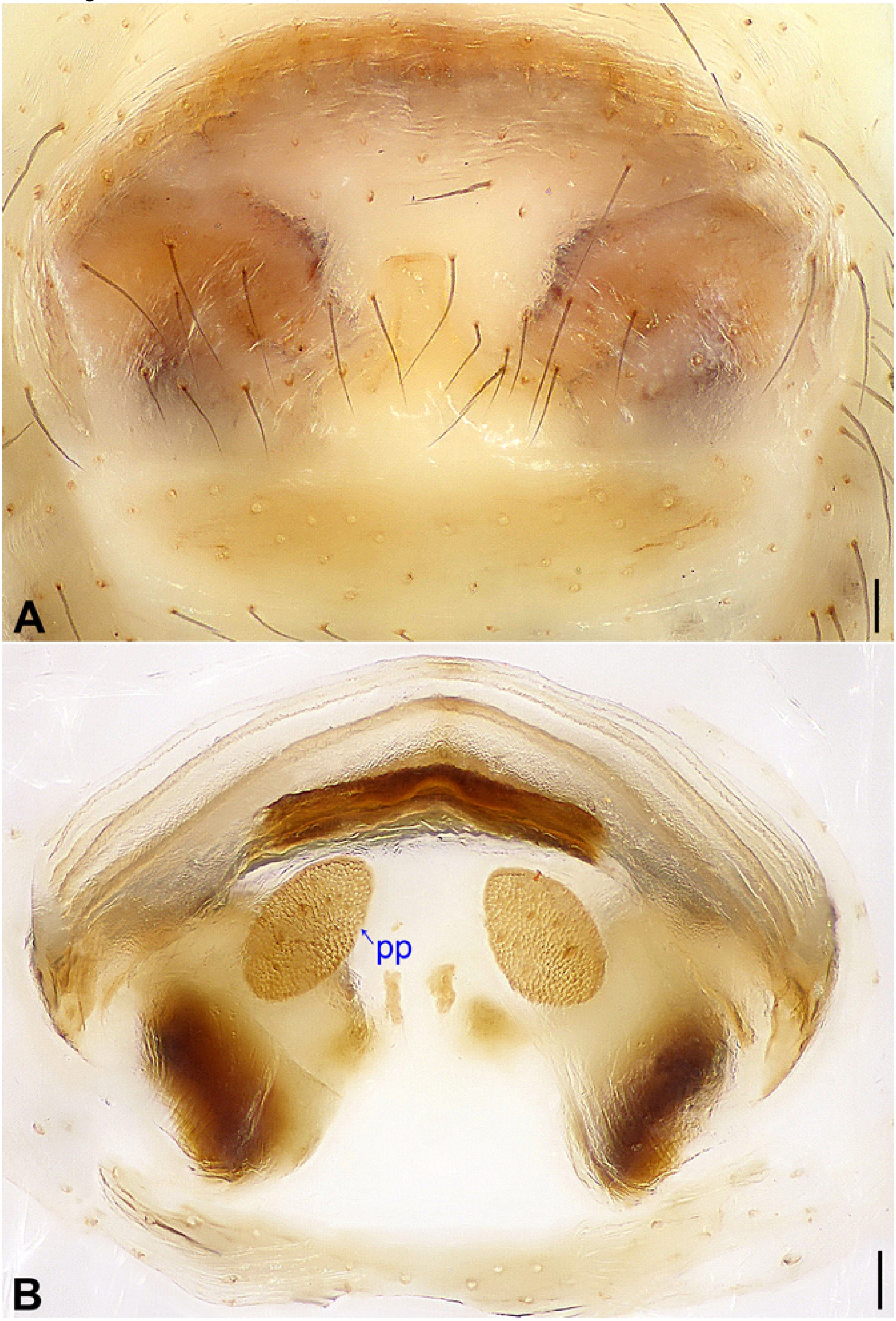

Figure S19. Illustrations of *Uthina saiyokensis* Yao & Li sp. nov., holotype male. A–B, pedipalp (A, prolateral view; B, retrolateral view); C–D, distal part of procursus (C, prolateral view; D, dorsal view). Scale bars: 0.20 (A–B), 0.05 (C–D)

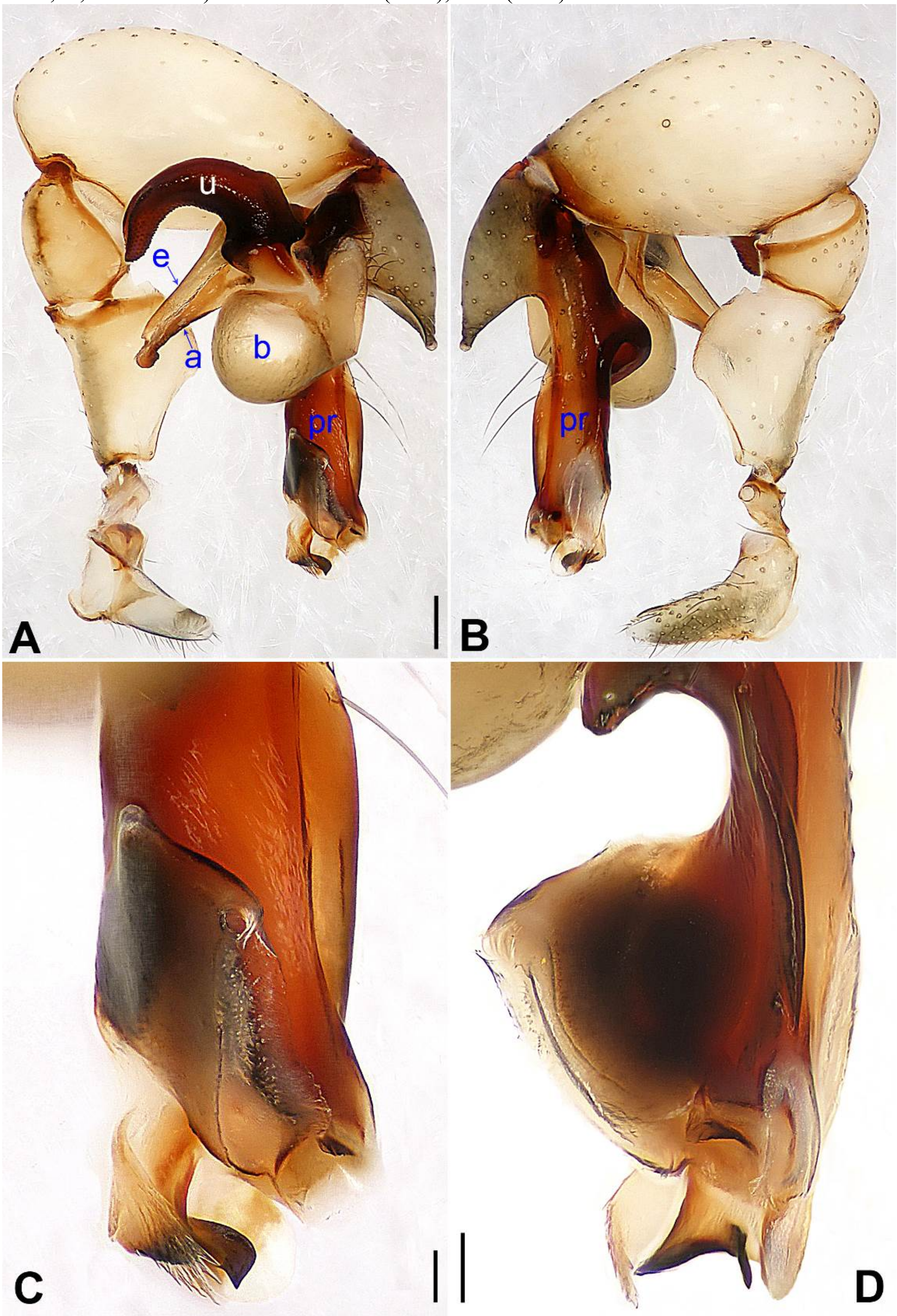

Figure S20. Illustrations of *Uthina saiyokensis* Yao & Li sp. nov., holotype male (A–B, D) and paratype female (C). A, bulbal apophyses, prolateral view, arrow points at distinct proximal apophysis on uncus; B–C, habitus, dorsal view; D, chelicerae, frontal view. Scale bars: 0.05 (A), 0.50 (B–C), 0.10 (D)

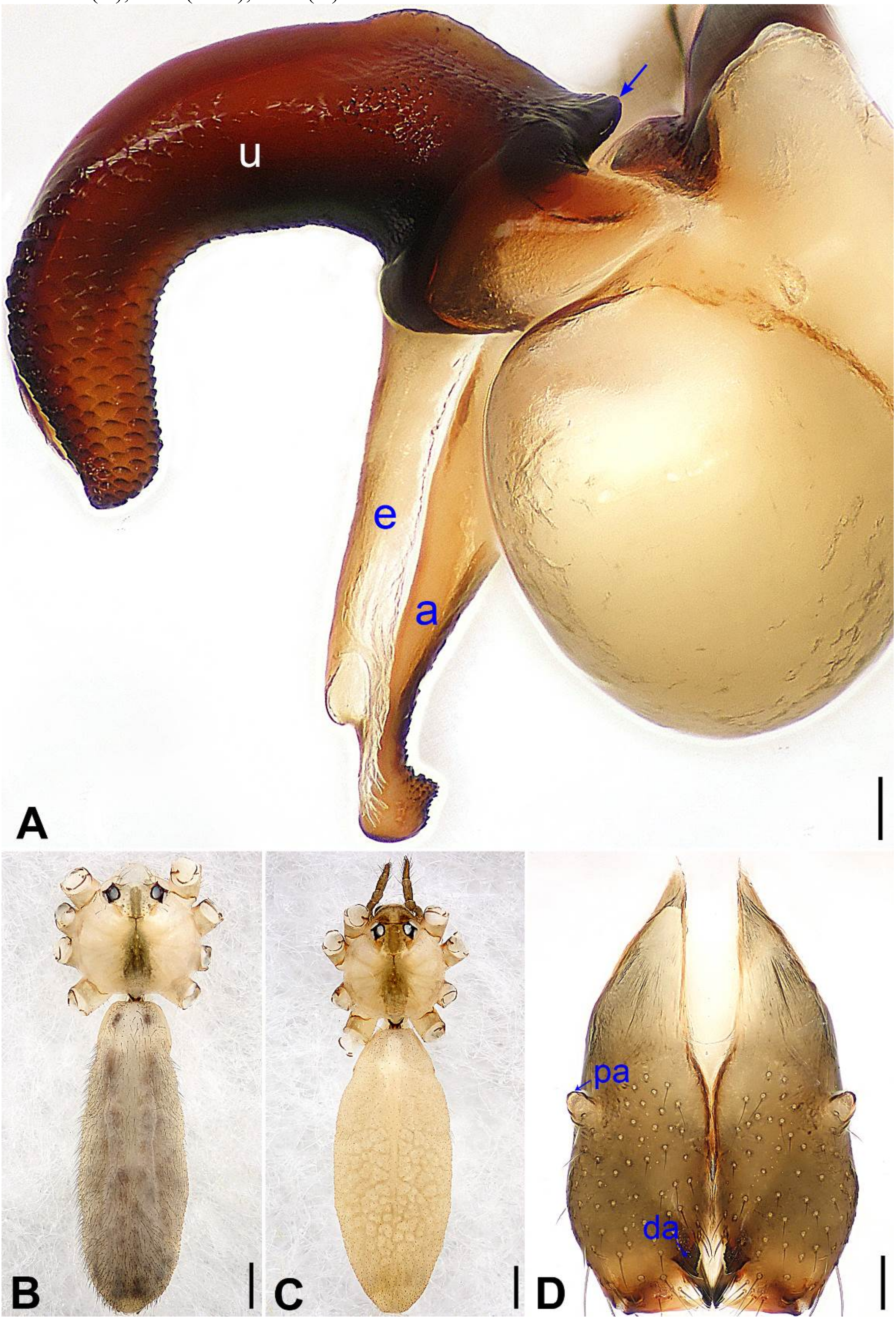

Figure S21. Illustrations of *Uthina saiyokensis* Yao & Li sp. nov., paratype female. A, external genitalia, ventral view; B, vulva, dorsal view. Scale bars: 0.05

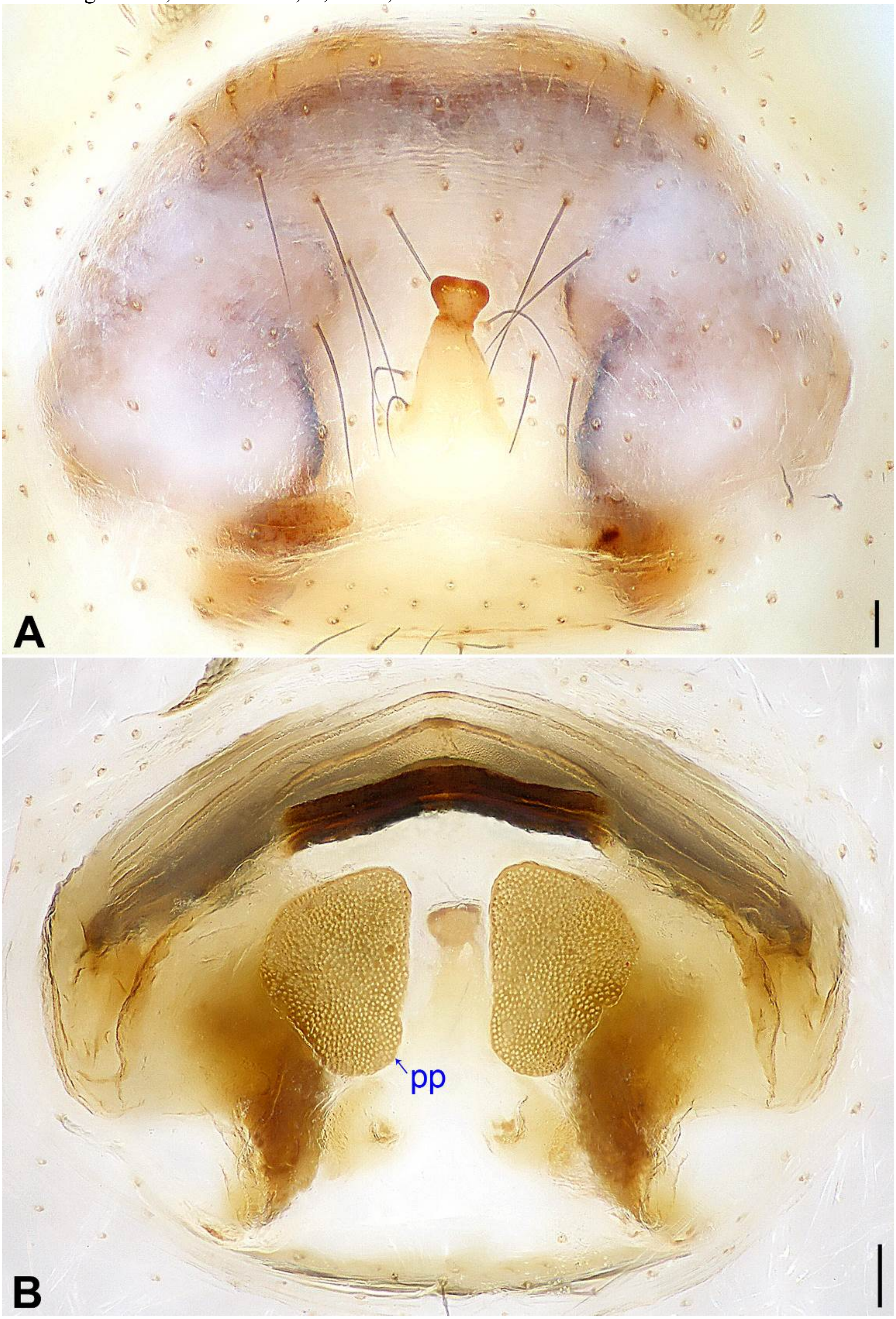

Figure S22. Illustrations of *Uthina sarikaensis* Yao & Li sp. nov., holotype female. A, external genitalia, ventral view; B, vulva, dorsal view; C–E, habitus (C, dorsal view; D, ventral view; E, lateral view). Scale bars: 0.05 (A–B), 0.50 (C–E)

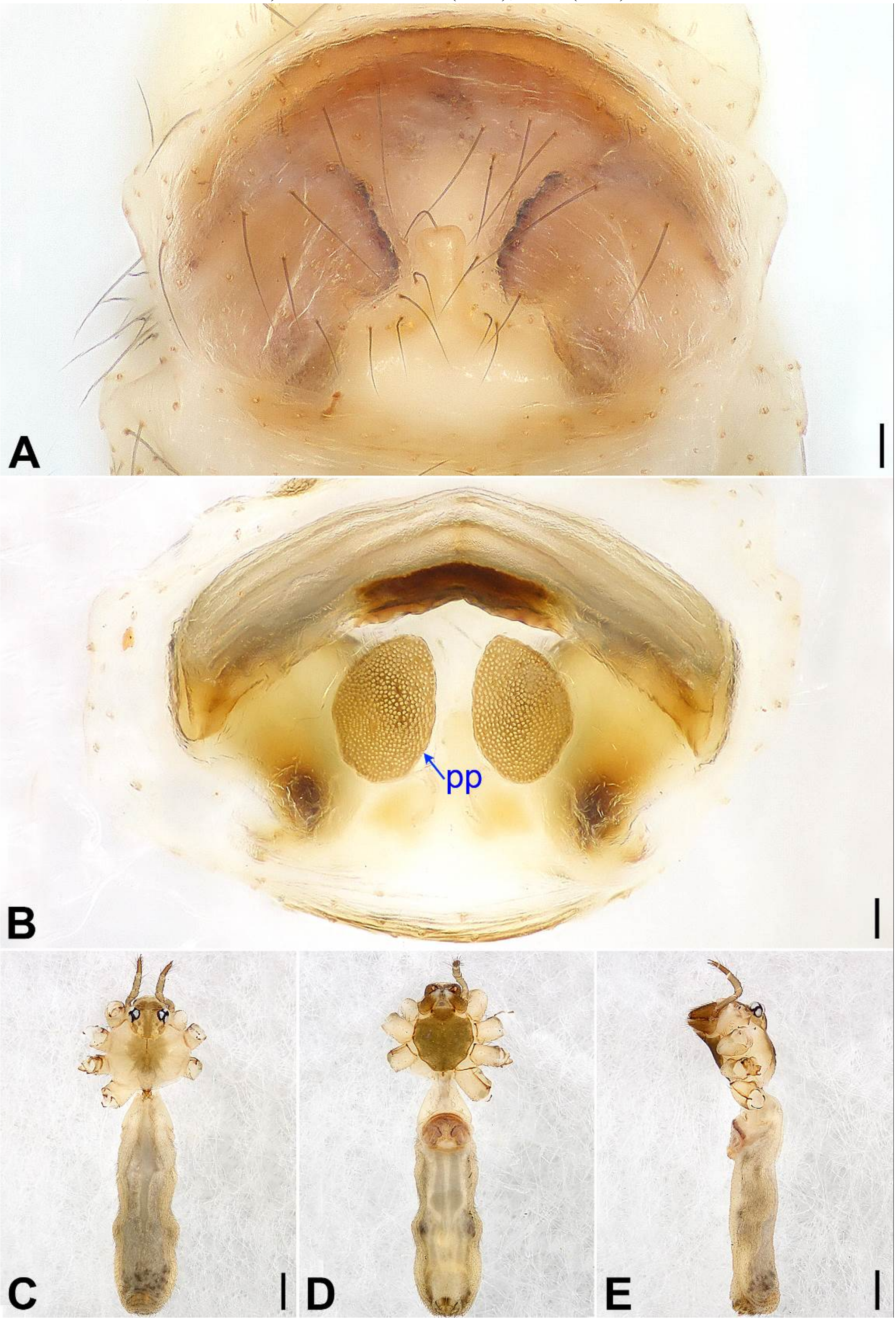

Figure S23. Illustrations of *Uthina sulawesiensis* Yao & Li sp. nov., holotype male. A–B, pedipalp (A, prolateral view; B, retrolateral view); C–D, distal part of procursus (C, prolateral view; D, dorsal view, arrow points at large angular apophysis). Scale bars: 0.10 (A–B), 0.05 (C–D)

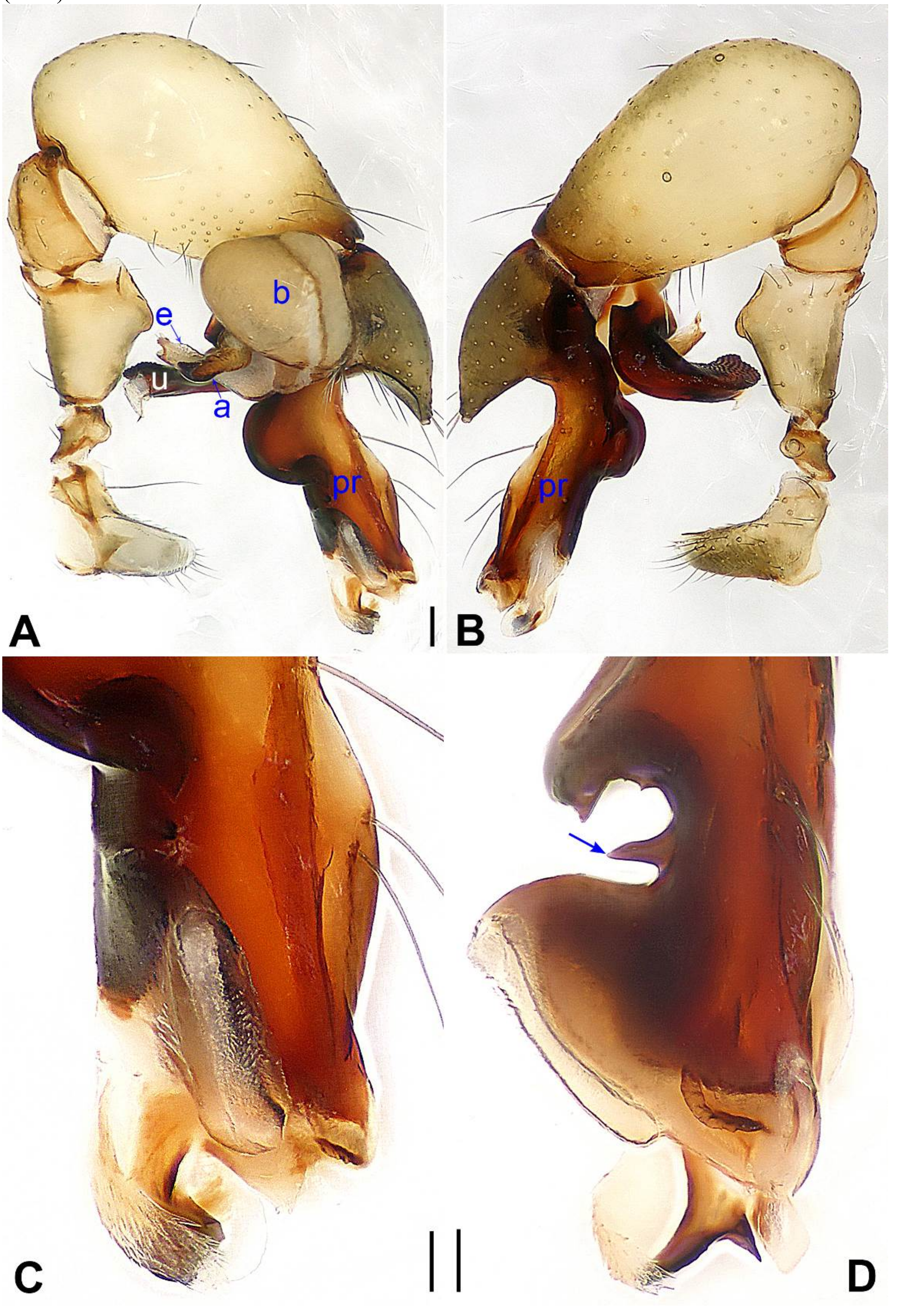

Figure S24. Illustrations of *Uthina sulawesiensis* Yao & Li sp. nov., holotype male (A–B, D) and paratype female (C). A, bulbal apophyses, prolateral view; B–C, habitus, dorsal view; D, chelicerae, frontal view. Scale bars: 0.10 (A, D), 0.50 (B–C)

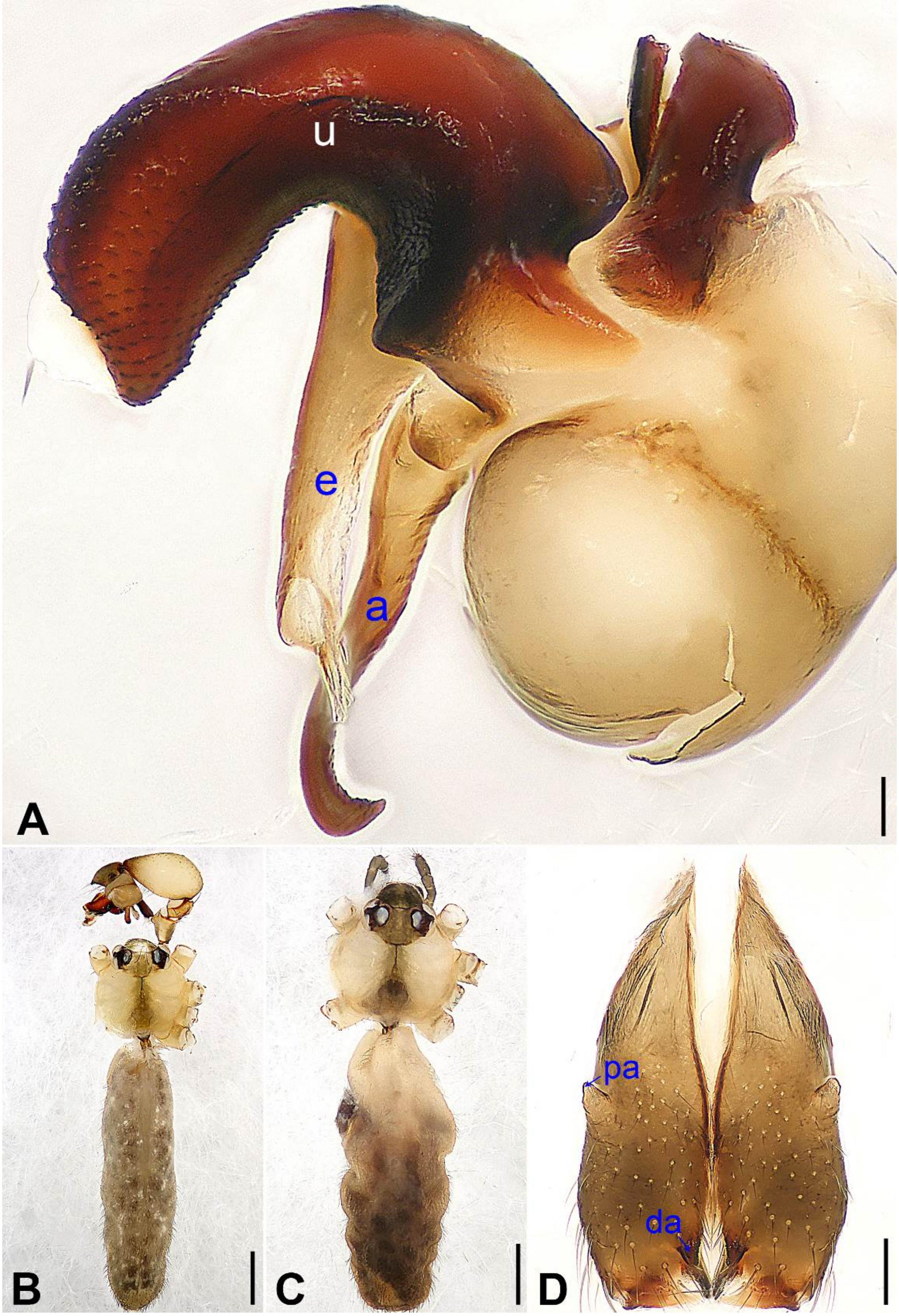

Figure S25. Illustrations of *Uthina sulawesiensis* Yao & Li sp. nov., paratype female. A, external genitalia, ventral view; B, vulva, dorsal view. Scale bars: 0.05

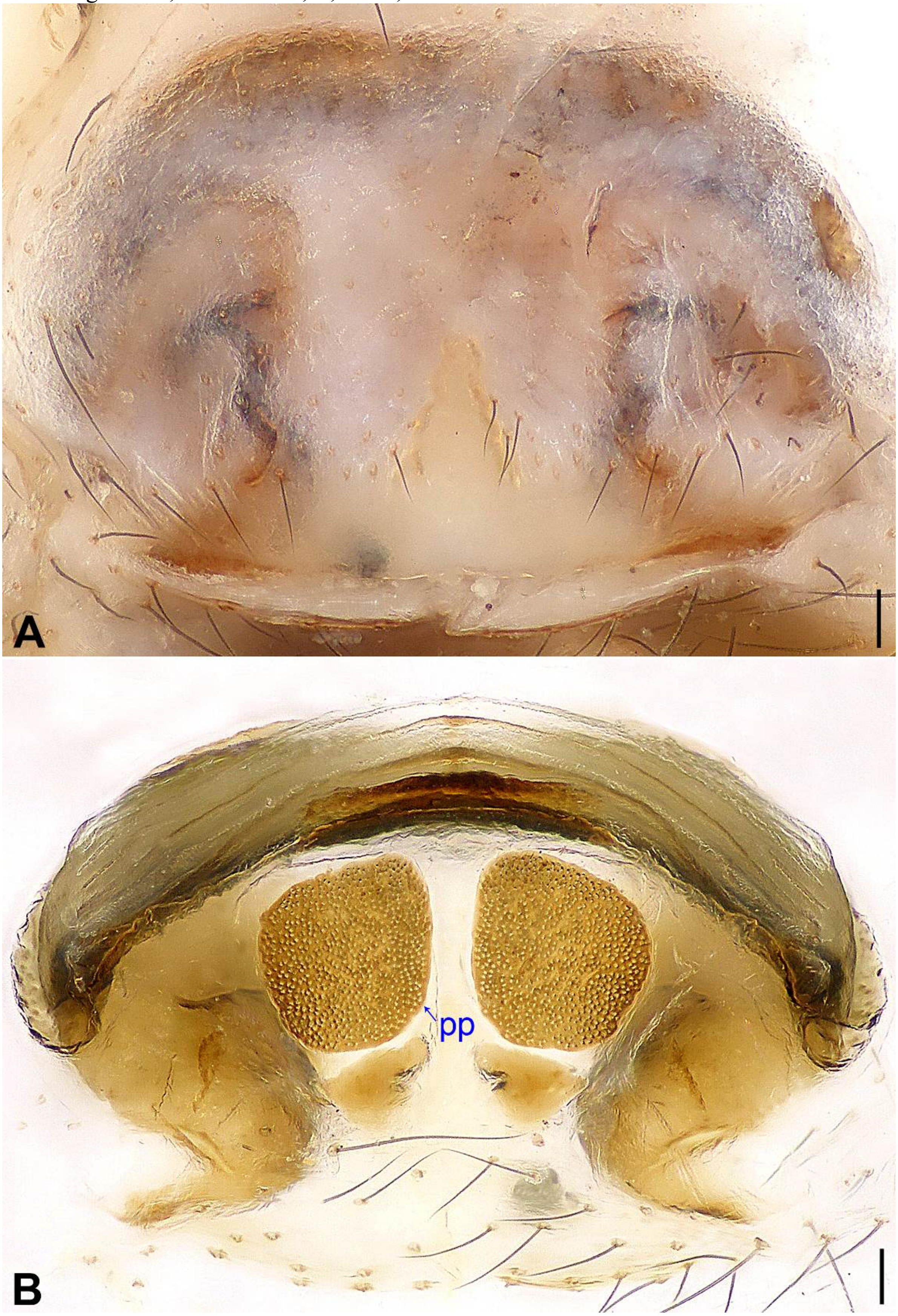

Figure S26. Illustrations of *Uthina wongpromi* Yao & Li sp. nov., holotype male. A–B, pedipalp (A, prolateral view; B, retrolateral view); C–D, distal part of procursus (C, prolateral view; D, dorsal view, arrow points at large bifurcated apophysis). Scale bars: 0.15 (A–B), 0.05 (C–D)

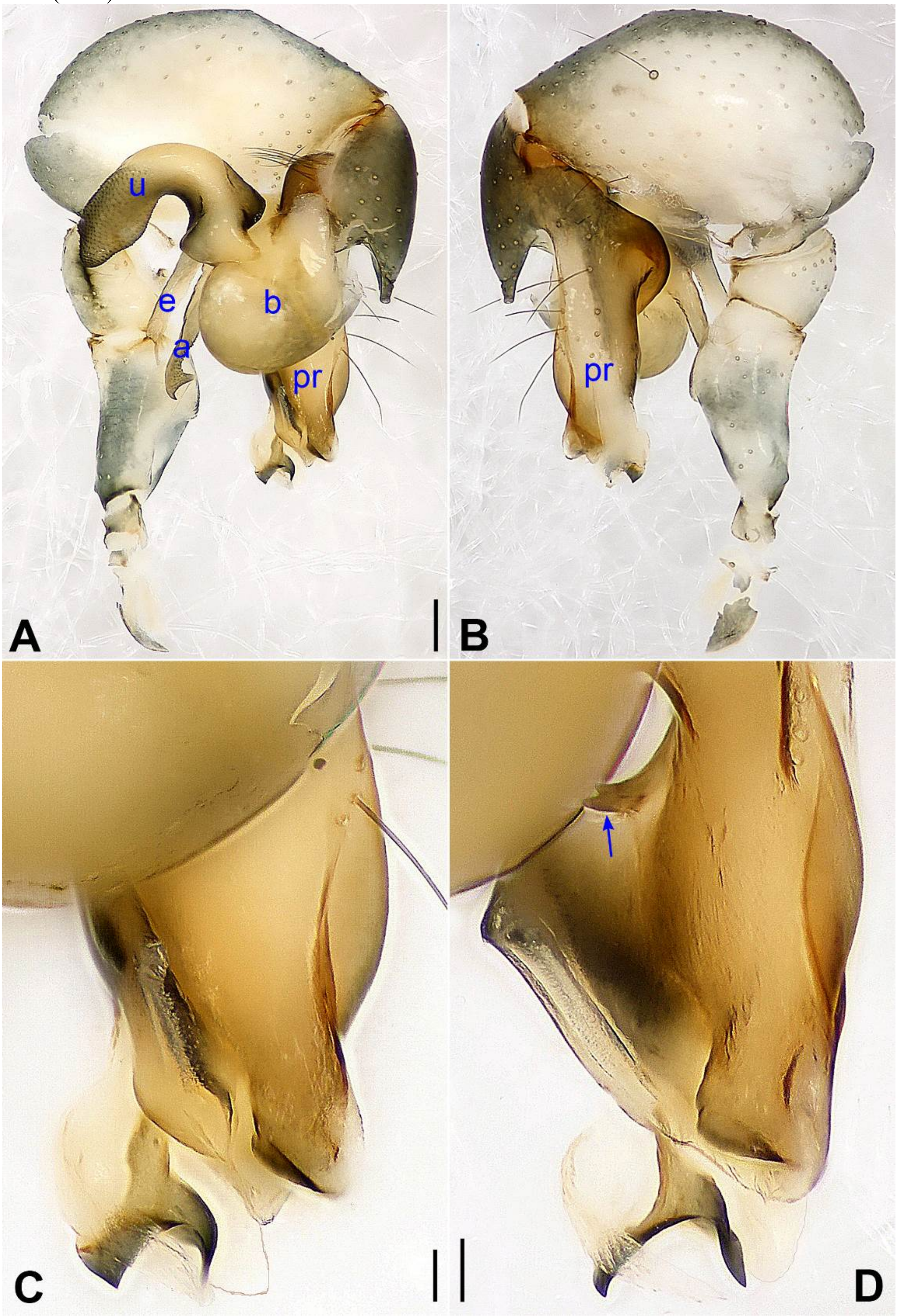

Figure S27. Illustrations of *Uthina wongpromi* Yao & Li sp. nov., holotype male. A, bulbal apophyses, prolateral view; B, habitus, dorsal view; C, chelicerae, frontal view. Scale bars: 0.10 (A, C), 0.50 (B)

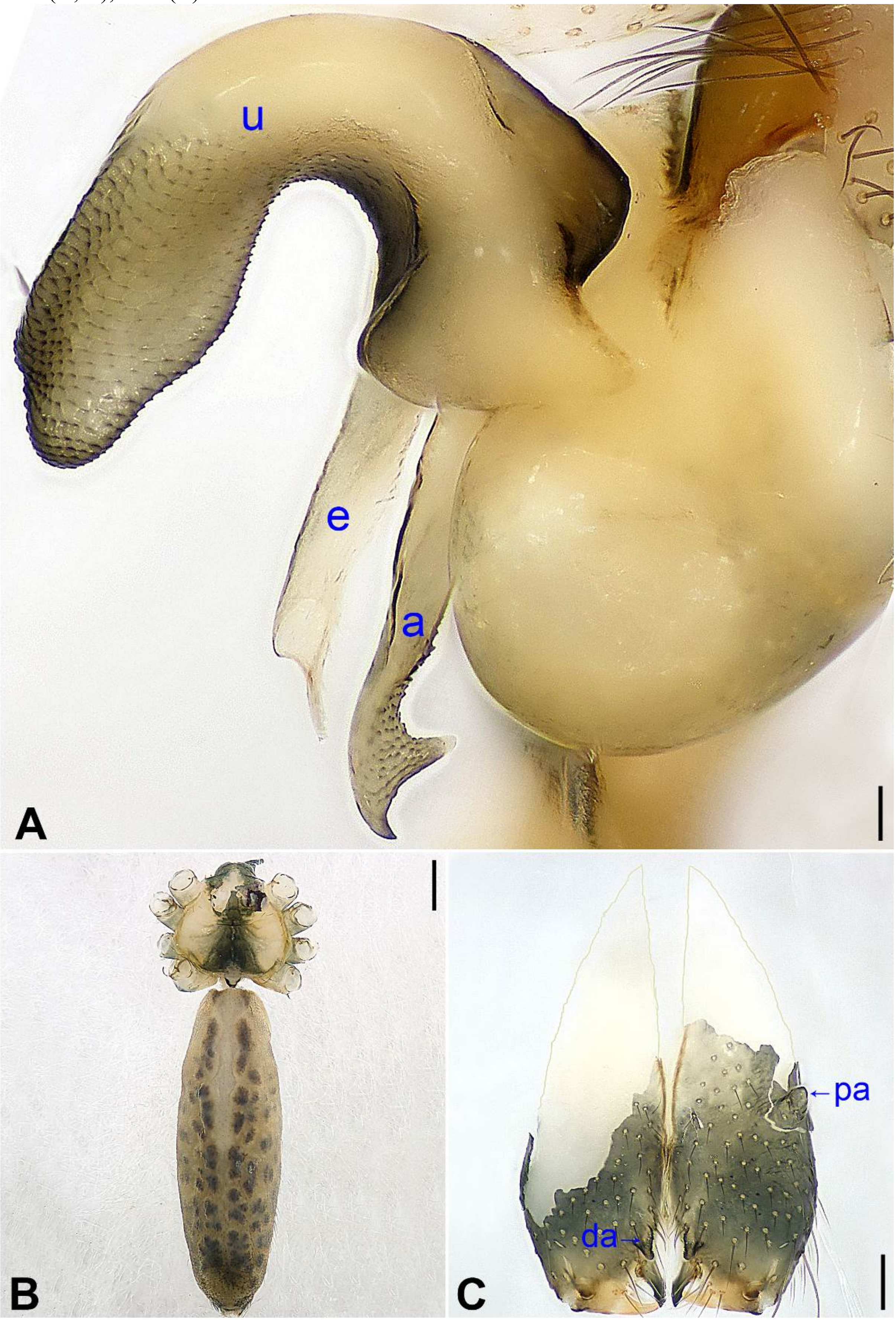

Figure S28. Illustrations of *Uthina yunchuni* Yao & Li sp. nov., holotype female. A, external genitalia, ventral view; B, vulva, dorsal view; C–E, habitus (C, dorsal view; D, ventral view; E, lateral view). Scale bars: 0.05 (A–B), 0.50 (C–E)

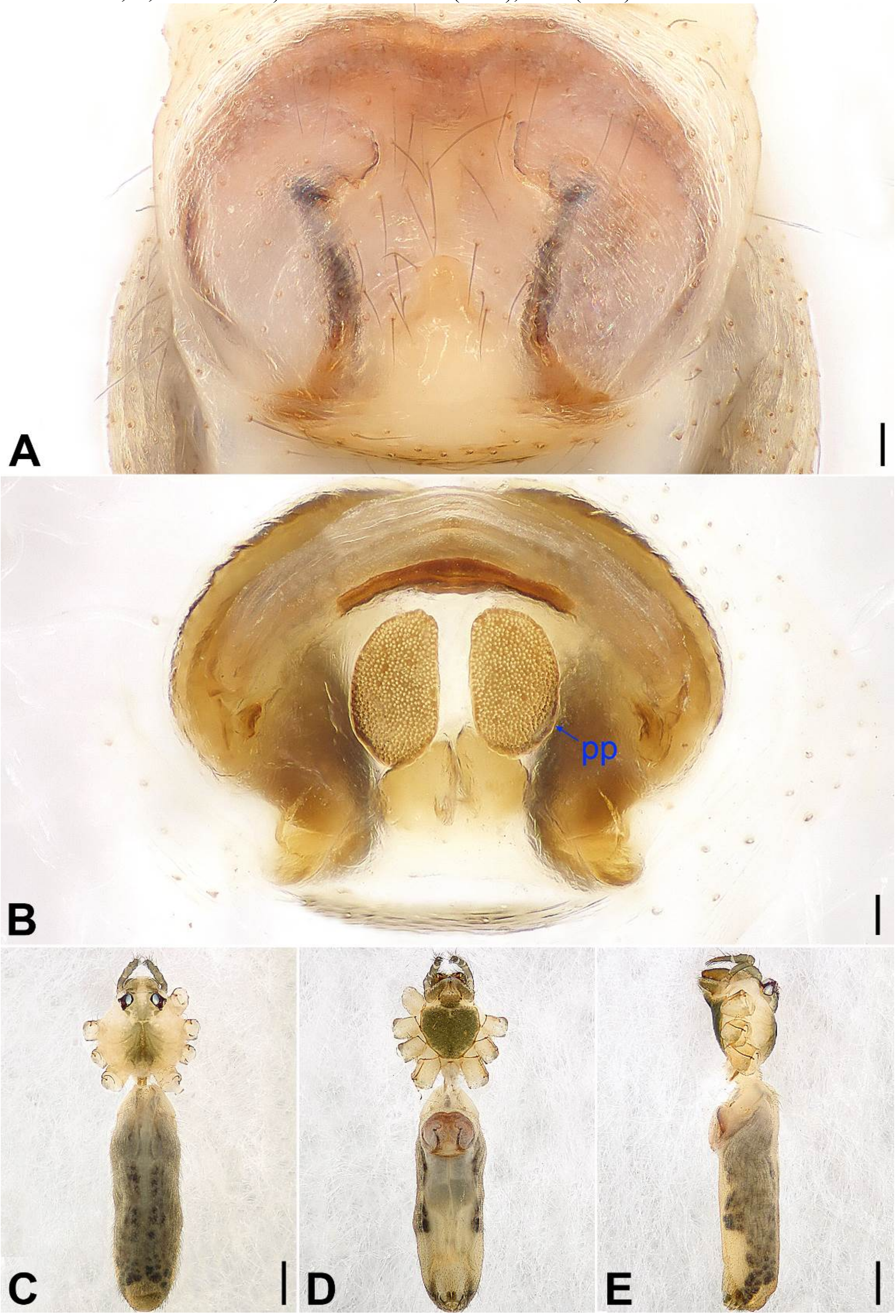

Figure S29. Illustrations of *Uthina zhigangi* Yao & Li sp. nov., holotype female. A, external genitalia, ventral view; B, vulva, dorsal view; C–E, habitus (C, dorsal view; D, ventral view; E, lateral view). Scale bars: 0.10 (A–B), 0.50 (C–E)

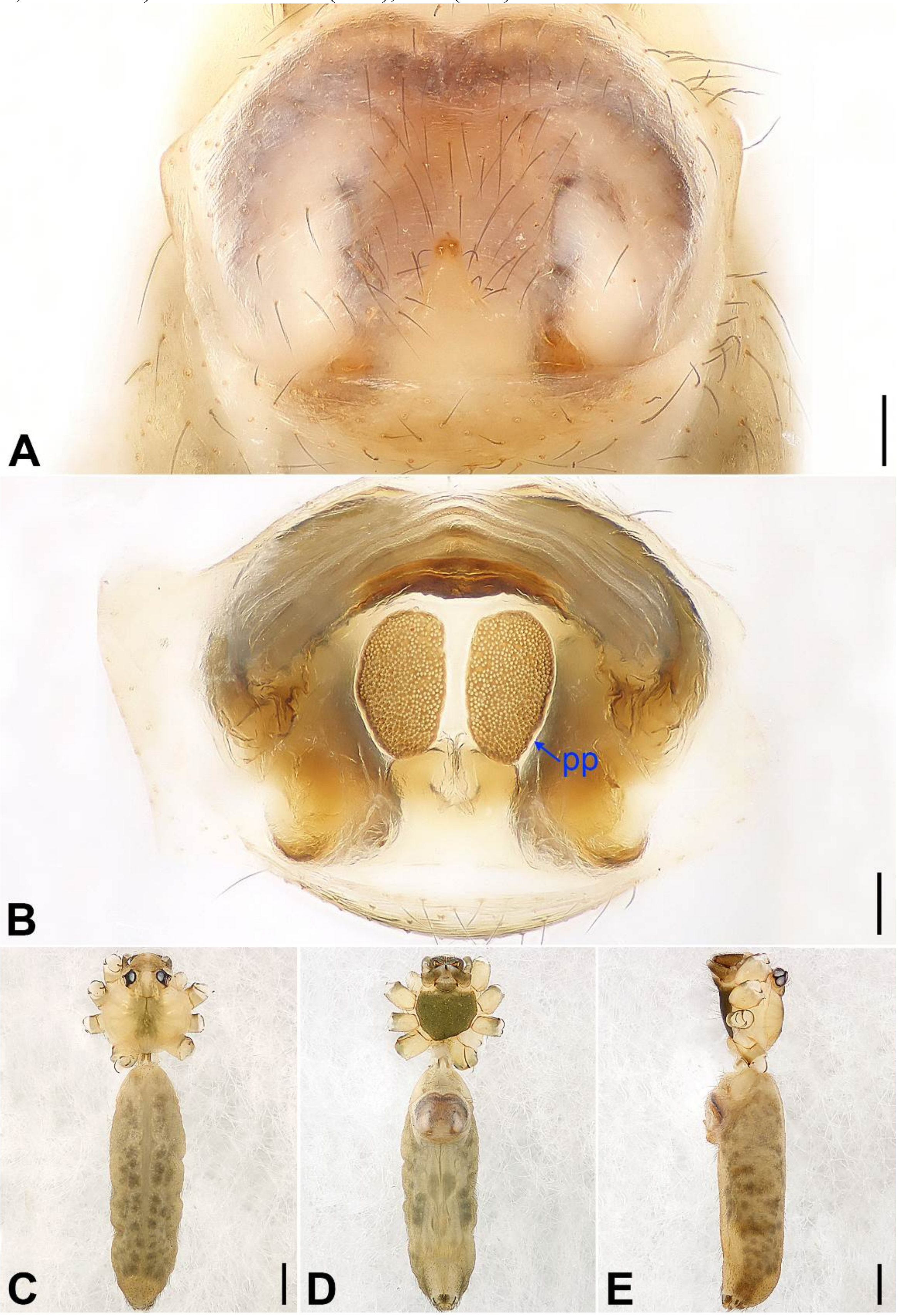

Supplement: Supplementary Information [file srep35757-s1.pdf]
